# Shape variation in the limb long bones of modern elephants reveals adaptations to body mass and habitat

**DOI:** 10.1111/joa.13827

**Published:** 2023-02-23

**Authors:** Camille Bader, Arnaud Delapré, Alexandra Houssaye

**Affiliations:** ^1^ Département Adaptations du Vivant, UMR 7179 Mécanismes adaptatifs et Évolution (MECADEV) CNRS/Muséum national d'Histoire naturelle Paris France; ^2^ UMR 7205, Institut de Systématique Evolution, Biodiversité (ISYEB), Muséum national d'Histoire naturelle, CNRS, SU, EPHE, UA Paris France

**Keywords:** 3D geometric morphometrics, bone external anatomy, elephants, functional morphology

## Abstract

During evolution, several vertebrate lineages have shown trends towards an increase in mass. Such a trend is associated with physiological and musculoskeletal changes necessary to carry and move an increasingly heavy body. Due to their prominent role in the support and movement of the body, limb long bones are highly affected by these shifts in body mass. Elephants are the heaviest living terrestrial mammals, displaying unique features allowing them to withstand their massive weight, such as the columnarity of their limbs, and as such are crucial to understand the evolution towards high body mass in land mammals. In this study, we investigate the shape variation of the six limb long bones among the modern elephants, *Elephas maximus* and *Loxodonta africana*, to understand the effect of body mass and habitat on the external anatomy of the bones. To do so, we use three‐dimensional geometric morphometrics (GMMs) and qualitative comparisons to describe the shape variation, at both the intraspecific and interspecific levels. Our results reveal that the two species share similar negative ontogenetic allometric patterns (i.e. becoming stouter with increased length) in their humerus and femur, but not in the other bones: the proximal epiphyses of the stylopod bones develop considerably during growth, while the distal epiphyses, which are involved in load distribution in the elbow and knee joints, are already massive in juveniles. We attribute this pattern to a weight‐bearing adaptation already present in young specimens. Among adults of the same species, bone robustness increases with body mass, so that heavier specimens display stouter bones allowing for a better mechanical load distribution. While this robustness variation is significant for the humerus only, all the other bones appear to follow the same pattern. This is particularly visible in the ulna and tibia, but less so in the femur, which suggests that the forelimb and hindlimb adapted differently to high body mass support. Robustness analyses, while significant for the humerus only, suggest more robust long bones in Asian elephants than in African savanna elephants. More specifically, GMMs and qualitative comparisons indicate that three bones are clearly distinct when comparing the two species: in *E. maximus* the humerus, the ulna and the tibia display enlarged areas of muscular insertions for muscles involved in joint and limb stabilization, as well as in limb rotation. These results suggest a higher limb compliance in Asian elephants, associated with a higher dexterity, which could be linked to their habitat and foraging habits.

## Graphical abstract

Elephants are the heaviest living terrestrial mammals, with unique features allowing them to withstand their massive weight. Here we investigate the shape variation of the six long bones among modern elephants to understand the effect of body mass and habitat on bone shape. Our results indicate strongly pronounced morphological variations, allowing for species distinction based on the external shape of the humerus, the ulna and the tibia. Asian elephants display more robust bones, suggesting a higher limb compliance which, associated with a higher dexterity, could be linked to their habitat and foraging habits.

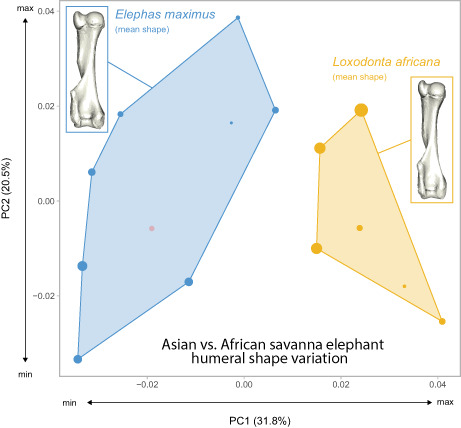

## 1 INTRODUCTION

Throughout time, several vertebrate lineages have shown trends towards an increase in mass (Baker et al., 2015; Bokma et al., 2016; Depéret, 1907; Raia et al., 2012), which comes with numerous benefits, such as an increased defence against predation and/or an extended longevity (Clauss et al., 2013; Hone & Benton, 2005), and associated trade‐offs, such as reduced athleticism (Hutchinson et al., 2003, 2006) and/or increased need for food and water (Demment & Van Soest, 1985). This evolutionary trend is associated with physiological and musculoskeletal changes necessary to accommodate an increase in size and mass (Biewener, 1989; Campione & Evans, 2012; Kleiber, 1961; Nielsen, 1997), notably to carry and move their heavy body.

If animals displaying these traits are said to be ‘graviportal’ (Hildebrand, 1974), the concept of graviportality, introduced by Gregory (1912) and Osborn (1929), remains debated (Mallet et al., 2019). Indeed, graviportality is defined by several anatomical and locomotion criteria: in addition to having a body mass of several hundreds of kilograms, graviportal taxa are supposed to display columnar limbs, associated with a relative lengthening of the stylopod and shortening of the autopod, and robust bones (i.e. larger shaft for a given length). Other criteria include large feet with enlarged adipose cushions, shorter phalanges and long strides associated with the inability to gallop (Coombs, 1978; Gregory, 1912; Osborn, 1929). However, while some modern taxa display several combinations of these criteria, few of them meet the entirety of the graviportal characteristics: rhinoceroses, while being the second heaviest land mammals after elephants and displaying specific skeletal adaptations to body weight support (Alexander & Pond, 1992; Mallet et al., 2019), are able to gallop, and do not meet the weight expectations for some of the earliest authors, so that Gregory (1912) and Osborn (1929) considered them as mediportal, although they were later considered as graviportal in several studies (Eisenmann & Guérin, 1984; Prothero & Sereno, 1982). Similarly, hippos have alternatively been considered as mediportal (Coombs, 1978; Ross, 1984) or graviportal (Alexander & Pond, 1992; Carrano, 1999; MacFadden, 2005; Stilson et al., 2016). Elephants, on the other hand, are the perfect example of the graviportal form, fulfilling all the criteria (Alexander & Pond, 1992; Coombs, 1978; Langman et al., 1995). However, despite their massive appearance and their inability to gallop, the kinematics of the elephants' running defies the traditional graviportal view of rigid limbs joints, displaying instead a surprising limb compliance (Hutchinson et al., 2008; Ren et al., 2008, 2010); thus indicating that their skeletal architecture is adapted to support a massive weight while allowing a certain flexibility.

Elephants are the only living representatives of the order Proboscidea, which conversely includes numerous extinct graviportal taxa (e.g. *Deinotherium*, *Mammut*, *Mammuthus*; see Gheerbrant and Tassy (2009)). At a larger taxonomic and evolutive scale, proboscideans were not the first to display fully graviportal bodies: sauropod dinosaurs were obligatory quadrupeds sharing a general graviportal form (Lefebvre et al., 2022; Rauhut et al., 2011; Sander et al., 2011). Their diversification towards a range of extreme gigantism was made possible by the acquisition of columnar limbs (straighter and positioned almost vertically), allowing to support a multi‐tons' body mass (Hildebrand, 1982). This specific ‘columnar’ architecture was convergently acquired in proboscideans, and can now be found in the elephant limbs only, making it a unique feature among extant vertebrates. Elephants display unique postural and locomotor adaptations which are reflected in their skeleton (Christiansen, 2007; Kokshenev & Christiansen, 2010), they are thus a particularly interesting group to analyse limb bone adaptation to heavy weight support.

Long bones provide a rigid frame on which muscles attach; as such, they play a prominent role in both the movement and the support of the body. Like all biological structures, limb anatomy results from the conjoined effects of phylogenetic, structural and functional constraints (e.g. Cubo, 2004; Gould, 2002; Seilacher, 1970). Among those, body mass is known to strongly affect limb bones and joints, so that their anatomy is highly impacted by shifts in body mass during evolution (Biewener, 1989; Hildebrand, 1982; Polly, 2007; Smuts & Bezuidenhout, 1994). Several studies on taxa that are considered graviportal have shown specific adaptations in the external anatomy of limb long bones in heavy mammalian taxa (Etienne et al., 2021; MacLaren & Nauwelaerts, 2016; MacLaren et al., 2018; Mallet et al., 2019, 2020). However, they also highlighted that limb adaptation to high body mass can differ considerably between species of similar weight, so that even among species that are considered graviportal, graviportality is not expressed in the same way: for example, hippos display stout limbs associated with the inability to trot or gallop, while rhinos possess more elongated limbs and are able of galloping (Wilson et al., 2011).

Among morphological adaptations to heavy weight, bone robustness is of particular interest. Indeed, limb bone robustness increases at a higher rate than body mass (Campione & Evans, 2012) so that heavy taxa display overall larger and stouter bones than smaller taxa, in order to withstand their increased weight. Consistently with the high body mass of proboscideans, their limb bones display a massive morphology (Christiansen, 2007).

The three extant species of elephants are geographically and taxonomically divided into the Asian genus *Elephas*, represented by a single species (*Elephas maximus*, the Asian elephant), and the African genus *Loxodonta*, represented by two species (*Loxodonta africana* and *Loxodonta cyclotis*, the African savanna and forest elephants). While the Asian elephant (*E. maximus*) can be found throughout the Indian subcontinent and Southeast Asia, the African savanna elephant (*L. africana*) occurs in Sub‐Saharan Africa in a variety of habitats, sometimes in subtropical and temperate forests but mostly desert and semi‐desert areas, whereas the African forest elephant (*L. cyclotis*) has a much more limited distribution, restricted almost exclusively to the rainforests of Cameroon, Democratic Republic of Congo and Gabon (Barnes et al., 1997; Blake et al., 2008).

Modern elephant species display a strong variation of size and body mass. Both *Loxodonta* species represent the extremes of height and mass: *Loxodonta africana* exhibits the most massive forms, reaching up to 8000 kilograms and 4 metres at the shoulder; at the opposite, *Loxodonta cyclotis* is the smallest extant elephant, with a body mass reaching up to 4000 kilograms and a shoulder height of 3 metres. *Elephas maximus* displays an intermediate weight (up to 6000 kilograms) and height (3.5 metres), although its morphology cannot be confused with the two *Loxodonta* species: the Asian elephant is easily distinguishable from the African species, with its small, rounded ears and its twin‐domed head, among other features. While it is possible to distinguish between Asian and African elephant species using the shape of their spine (*E. maximus* having a more convex back than *L. africana*) or their autopod (differing number of toenails), their limb long bones are not known to bear specific morphological features that would allow species distinction (Todd, 2010; West, 2006 and references therein). However, given the mass discrepancy between the species, we could expect to observe a shape variation between the limb bones of Asian and African savanna elephants.

While limb long bones share a function of weight support, they do not participate equally: unlike most quadrupedal mammals, the ulna plays a major role in weight bearing compared to the radius (Bertram & Biewener, 1992; Smuts & Bezuidenhout, 1993); similarly, the tibia is the main weight bearer in the hindlimb zeugopod, while the fibula is reduced (Smuts & Bezuidenhout, 1994). Due to their position closer to the trunk, bones of the stylopod bear more muscular insertions with the pectoral and pelvic girdles than do bones of the zeugopod (Shindo & Mori, 1956a, 1956b) and thus face different constraints. The six bones might then be affected differently by body mass variations, so that we could expect to observe varying degrees of shape variation linked to heavy weight support among them. Additionally, like in most quadrupedal mammals the centre of mass in elephants is closer to the forelimb than to the hindlimb, so that the forelimb elements carry more weight (60% of the body mass) than the hindlimb elements (Etienne et al., 2021; Hildebrand, 1974; Lessertisseur & Saban, 1967; Polly, 2007, Ren et al., 2010). We could thus expect to see more shape variation linked to heavy weight support in the bones of the forelimb than in bones of the hindlimbs.

While several studies have described the limb muscular (Eales, 1925; Shindo & Mori, 1956a, 1956b; Weissengruber & Forstenpointner, 2004) and skeletal anatomy (Eales, 1925; Hutchinson et al., 2008; Smuts & Bezuidenhout, 1993, 1994; Weissengruber, Egger, et al., 2006; Weissengruber, Fuss, et al., 2006) of elephants, as well as their locomotor kinematics (Hutchinson et al., 2006; Langman et al., 1995, 2012; Ren et al., 2008, 2010), to our knowledge no study has yet investigated the shape variation of the six limb long bones conjointly.

3D GMMs have been proven extremely useful to characterize shape variation on such bones. They have been used to study the influence of locomotion and body mass in small carnivorans (Fabre, Cornette, Peigné, et al., 2013; Fabre, Cornette, Argot et al., 2013; Fabre et al., 2015; Figueirido et al., 2015; Martín‐Serra et al., 2014), rodents (Álvarez et al., 2013; Wölfer et al., 2019), xenarthrans (Alfieri et al., 2022) and primates (Botton‐Divet & Nyakatura, 2021), as well as in heavier taxa like Suidae (e.g. Harbers et al., 2020). Similarly, they have been used to study the effect of high body mass in mammals (Mallet et al., 2019) and reptiles (Lefebvre et al., 2022; Pintore et al., 2022), but not on proboscideans bones.

In addition to linear measurements of the robustness of the shaft, 3D GMMs will allow a more precise quantification of the shape (variation) along the whole bone. Additionally, since the diaphyseal circumference cannot not be obtained for the radius and ulna, 3D GMMs will compensate for the absence of robustness calculations for these bones.

We link here the shape of the bones to their function of weight support in a graviportal species. While body mass data were not available, the link between size (centroid size and diaphyseal circumference) and mass has previously been established in numerous species, including elephants (Campione & Evans, 2012) and other heavy taxa such as tapirs and rhinos (MacLaren et al., 2018; Mallet et al., 2019). Thus, we chose to use the various size measurements as proxies to infer mass variation in our sample. Our study aims to determine the adaptations of the limb long bones to a heavy weight in elephant species as a whole, so that the body mass inferences from bone size will allow us to describe bone shape adaptations reflecting the generally massive weight of the species.

Here we propose to analyse the external morphology of the limb long bones in a sample of modern elephants: we quantify the intraspecific and interspecific shape and robustness variations in *Loxodonta africana* and *Elephas maximus*, and interpret them in relation to their relative body mass and habitat. In order to do so, (1) we first investigate the morphological variation of the six long bones at the intraspecific level, estimating the potential effect of ontogeny on bone shape allometry using a small sample of juvenile specimens, (2) then we explore the interspecific shape variation between the two species, taking their body proportions, habitat and locomotor behaviour into account and (3) we compare the amount of shape variation in the six bones, investigating which part(s) of the limbs are most affected by body mass and habitat.

## 2 MATERIALS AND METHODS

### 2.1 Sample

We selected a total of 97 bones from 32 elephant specimens from several European and American institutions, belonging to the three extant elephant species. While the distinction between the *Loxodonta* and *Elephas* genera in museum's collections is reliable (based on the country of origin and the shape of the skull when it is present), the distinction between the African species *L. africana* and *L. cyclotis* is generally not possible: the separation of the *Loxodonta* genus into two species is very recent (Roca et al., 2001; Rohland et al., 2010), so that most specimens originating from Africa are registered as *L. africana* in collections. Each specimen of this species is thus susceptible to have been incorrectly diagnosed as *L. africana* and to actually belong to the *L. cyclotis* species, with the exception of two specimens (MNHN‐ZM‐AC‐1907‐49 and MNHN‐ZM‐AC‐1938‐375) from which DNA samples have been obtained and analysed in an unrelated study (R. Debruyne, pers. comm.). However, since African forest elephants are assumed to be less easily and thus less often hunted (BYH: Forest elephant… c2015‐2022), we assumed that the specimens of this sample were correctly attributed, although genetic analyses or identifications using cranio‐dental characteristics might prove otherwise (Table 1). We thus kept each museum's species diagnosis, resulting in a sample containing a vast majority of specimens from the *L. africana* and *E. maximus* species, and a single official *L. cyclotis* specimen.

**TABLE 1:** Sample studied.

Taxon	Institution	Specimen number	H	R	U	Fe	T	Fi	Sex	Age	AM
*Elephas maximus*	IMNH	1486	X	X	X	X	X	X	NA	A	LS
*Elephas maximus*	MNHN	ZM‐AC‐1883‐1786				X			NA	A	CT
*Elephas maximus*	MNHN	ZM‐AC‐1896‐17	X	X	X	X	X		M	A	SS
*Elephas maximus*	MNHN	ZM‐AC‐1896‐19	X	X	X	X			M	A	SS
*Elephas maximus*	MNHN	ZM‐AC‐1907‐263	X	X					F	S	SS
*Elephas maximus*	MNHN	ZM‐AC‐1936‐280					X		M	S	CT
*Elephas maximus*	MNHN	ZM‐AC‐1983‐082	X	X	X	X	X	X	F	A	SS
*Elephas maximus*	MNHN	ZM‐AC‐1998‐6	X	X	X	X	X	X	M	A	SS
*Elephas maximus*	NHMW	2526	X			X			NA	J	P
*Elephas maximus*	NHMW	2828				X	X	X	NA	J	P
*Elephas maximus*	NHMW	4012	X			X			NA	A	P
*Elephas maximus*	ZSM	1953/153	X	X	X	X	X	X	NA	A	SS
*Elephas maximus*	ZSM	1962/340	X	X	X				NA	J	P
*Elephas maximus*	ZSM	Unnumbered	X	X	X	X	X		NA	A	P
*Elephas maximus*	NHMB	936						X	NA	A	P
*Elephas maximus*	NHMB	46,024						X	NA	A	P
*Loxodonta africana*	MNHN	ZM‐AC‐1855‐11						X	NA	A	SS
*Loxodonta africana*	MNHN	ZM‐AC‐1907‐49	X		X	X	X	X	M	A	SS
*Loxodonta africana*	MNHN	ZM‐AC‐1938‐375	X	X					NA	A	SS
*Loxodonta africana*	MNHN	ZM‐AC‐1986‐060	X						F	A	CT
*Loxodonta africana*	NHMW	Unnumbered	X		X	X	X		NA	A	P
*Loxodonta africana*	RBINS	10,858	X	X	X	X		X	NA	A	SS
*Loxodonta africana*	ZSM	1962/252		X	X	X	X		NA	A	P
*Loxodonta africana*	ZSM	1978/182	X	X	X	X	X	X	NA	A	SS
*Loxodonta cyclotis*	RBINS	12,677	X	X	X	X	X	X	NA	A	SS
NA	MNHN	ZM‐AC‐1977‐30D				X			NA	A	SS
NA	MNHN	ZM‐AC‐1977‐30 E				X			NA	A	SS
NA	MNHN	ZM‐AC‐1977‐30F				X			NA	A	SS
NA	MNHN	ZM‐AC‐1977‐30G				X			NA	A	SS
NA	MNHN	ZM‐AC‐1977‐30H				X			NA	A	SS
NA	MNHN	ZM‐AC‐1977‐30I				X			NA	A	SS
NA	MNHN	ZM‐AC‐1977‐30 J				X			NA	S	SS
NA	MNHN	ZM‐AC‐1977‐30 K				X			NA	S	SS
NA	MNHN	ZM‐AC‐1977‐30 M				X			NA	A	SS

*Note*: Age: (j), juvenile, S, subadult, (a), adult. AM, acquisition mode: P, photogrammetry; SS, surface scanner; CT, CT scan; LS, laser scanner. Institutional codes: IMNH, Idaho Museum of Natural History, Pocatello (USA); MNHN, Muséum national d'Histoire Naturelle, Paris (France); NHMW, Naturhistorisches Museum Wien, Vienna (Austria); RBINS, Royal Belgian Institute of Natural Sciences, Brussels (Belgium); ZSM, Zoologische Staatssammlung München, Munich (Germany).

Abbreviations: Fe, femur; Fi, fibula; H, humerus; NA, not available; R, radius; Sex: F, female; M, male; T, tibia; U, ulna.

Our sample was composed of 18 humeri, 14 radii, 14 ulnae, 26 femora, 13 tibiae and 12 fibulae, depending on availability (Table 1). Nine femora were not diagnosed. Those specimens were referenced in the archives of the Muséum national d'Histoire Naturelle (MNHN) as a set of undetermined femora marked with a Chinese character (translated to ‘profit’), which indicates a probable Asian origin. However, their actual determination being uncertain, analyses were performed in order to ascertain to which species they could belong, and all analyses on the femur were performed twice: once with these specimens considered as Asian elephant, and once without these specimens.

Age determination was provided by the institutions in some cases. When no data on the age of the specimens were available, we determined the ontogenetic stage (juvenile, subadult, adult) based on the level of fusion and development of the epiphyses (juvenile: unfused epiphyses, subadult: visible epiphyseal plate line, adult: fully fused epiphyses). The sex of the specimens, as well as their exact origin and captivity state, were generally unknown. As such, we could not account for these parameters in our analyses.

### 2.2 3D imaging

A large part of the sample (61 bones) was digitized using a structured‐light three‐dimensional scanner (Artec Eva) and reconstructed with Artec Studio Professional software (version 12.1.6.16, Artec 3D, 2018). Complementarily, 25 bones were digitized using photogrammetry, following Mallison & Wings (2014) and Fau et al. (2016). Pictures were taken with a digital camera (Nikon D5500, Nikon Inc., 50 mm lens) all around each bone and aligned to create a 3D model using Agisoft Photoscan software (version 1.4.0.5076, Agisoft, 2017).

Additionally, three bones were CT scanned for a later study; they were scanned using high‐resolution computed tomography at the AST‐RX platform (UMS 2700, Muséum National d'Histoire Naturelle, Paris) with reconstructions performed using X‐Act (RX‐Solutions). Voxel size varies from 86 μm to 330 μm depending on specimen size. The external surface of these bones was segmented and reconstructed in VGStudio MAX Software (2016). Each mesh was decimated to reach 250,000 vertices and 500,000 faces using MeshLab software (version 2020.07, Cignoni et al., 2008). Finally, 3D models from the specimen IMNH‐1486 were obtained from MorphoSource (six bones); they had been created using a laser scanner (Faro Edge Arm, Idaho Virtualization Lab).

Previous research on similarly sized bones has found no major differences in 3D models created using these two methods (Díez Díaz et al., 2021; Fau et al., 2016; Petti et al., 2008; Remondino et al., 2010; Soodmand et al., 2018; Waltenberger et al., 2021).

The right bones were symmetrized arbitrarily on the left side for the purpose of the analyses using Meshlab software.

### 2.3 Geometric morphometrics

#### 2.3.1 Landmark digitization

We defined the shape of the bones using anatomical landmarks, and curve and surface sliding semi‐landmarks, as described by Gunz et al. (2005), Gunz and Mitteroecker (2013), and Botton‐Divet et al. (2016). We used 14 anatomical landmarks for the humerus, 12 for the radius, 15 for the ulna, 16 for the femur, 18 for the tibia and 10 for the fibula (Figure [Link], [Link]; Tables S1–S6). Each curve is bordered by anatomical landmarks as recommended by Gunz and Mitteroecker (2013). All landmarks and curves were placed using the IDAV Landmark software (version 3.0, Wiley et al., 2005).

For some specimens, the radius and ulna could not be separated because the two bones were fused together, so that we could not access the surface of contact between them. In order to place homologous landmarks on the entire sample of radius and ulna, we placed curves around the contact zones to delimit the surface of each bone, so that semi‐landmarks could not slide out of the defined area. We placed the same curves on isolated radii and ulnae so that all surfaces considered were homologous (Figures S2 and S3). These curves were removed after the sliding landmark procedure and before performing the shape analysis, so that they are not included in our analyses, following Pintore et al. (2022).

For each bone, surface semi‐landmarks were manually placed on a template, created from a single specimen selected beforehand for its mean conformation with the ‘findMeanSpec’ function of the geomorph package (Adams & Otárola‐Castillo, 2013) of R (R Core Team, 2020, version 4.0.2), using RStudio (RStudio Team, 2020, version 1.3.959–1). Each bone template was then used to project the semi‐landmarks onto the surface of the other specimens of the dataset using the ‘placePatch’ function of the Morpho package (Schlager, 2017). Projection was followed by a relaxation step to ensure that the projected points matched the actual surface of the mesh. The curve and surface semi‐landmarks were slid using the minimizing bending energy algorithm (Bookstein, 1998). The landmarks and semi‐landmarks could therefore be treated as geometrically homologous from one bone to the next.

#### 2.3.2 Generalized Procrustes analyses

Following the sliding of all semi‐landmarks, all the specimens were superimposed using a Generalized Procrustes Analysis (GPA) (Bookstein, 1992; Rohlf & Slice, 1990) to remove the effects of position, orientation and size and to isolate the shape information (3D landmarks coordinates). Additionally, GPA produces centroid size (Cs), defined as the square root of the summed squared distances of each landmark and the centroid of the landmarks' configuration. We used PCA to visualize the specimen distribution in the morphospace.

For each bone, the error in digitizing the landmarks was assessed by a repeatability test. Ten recordings of anatomical landmarks were made on three visually similar specimens of the same species and analysed by principal component analysis (PCA). In order to maximize human variation, the landmarks were placed in two sessions of five measurements separated by several days (the landmarks were place first on one bone, followed by the second and the third, for each iteration). All repeated measurements produced three well‐separated clusters on the first two principal components (PCs), indicating that measurement error was negligible compared to the biological differentiation among the three specimens.

Patterns of shape variation were visualized using PCAs, computed on each type of bone. In order to display shape deformation along the principal axes, we computed theoretical consensus shapes of our sample and used it to calculate TPS deformation of the template meshes. We then used this newly created consensus mesh to compute theoretical shapes associated with the maximum and minimum of both axes of each PCA, as well as mean shapes of each bone for each species. To compare adult and juvenile specimens of *E. maximus*, mean shapes of adult and juveniles were computed separately. To compare adult specimens of *E. maximus* and *L. africana*, mean shapes of the adult specimens of *L. africana* were additionally computed (the mean shape of adult *E. maximus* being the same as used previously). The *L. cyclotis* specimen was included in the PCAs to assess its position within the shape variation of the whole sample; qualitative comparisons were made using the meshes of the six bones. GMM procedures were performed with the ‘geomorph’ (version 3.0.7, Adams & Otárola‐Castillo, 2013; Schlager, 2017) functions and the ‘Morpho’ (version 2.6) packages of R software (4.0.2, R Core Team). To visualize patterns of shape similarities among our sample, we performed neighbour‐joining trees on each type of bone, using the Euclidean distances between each specimen's bone shape computed from their PCA scores using the ‘ape’ package (Paradis & Schliep, 2019).

In order to assess whether femoral shape could be as good indicator of species determination, we used the k‐nearest neighbour (k‐NN) algorithm (Ripley, 2007; Venables et al., 2002) in the ‘class’ package (Venables et al., 2002). This nonparametric method consists of classifying an object into a predefined group according to its Euclidean distance with its k‐NN (k being a natural number). We tested with k ranging from 1 to n‐1, *n* being the smallest number of individuals within a group, then calculated the mean of the values obtained. The single *L. cyclotis* specimen was not included in the k‐NN analyses for obvious reasons of sample size.

### 2.4 Robustness parameters

In order to assess the robustness of the bones, we measured the circumference of the diaphyses at their thinnest part (Ci), and the maximal length (MaxL) of the bones. Bones were aligned along their longitudinal axis following Ruff (2002). Circumferences were obtained using the CloudCompare software (version 2.12.0, http://www.cloudcompare.org) for each bone except for the radius and ulna, which could not be separated in several specimens. Radius and ulna were thus excluded from analyses using circumference as a parameter. Bone maximal length was obtained virtually by placing reference points on the 3D models and measuring the distance between them using the Landmark software. Robustness (Rb) was defined as the ratio of minimal diaphyseal circumference to maximal length of the bones (Ci/MaxL). The difference in adult bone length, circumference and robustness between species was tested by performing *t* tests.

### 2.5 Statistics

Allometry can be defined as the covariation of size with shape (Gould, 1966; Klingenberg, 2016). In order to investigate the morphological variation of the six long bones at the intraspecific level, we checked for allometry among the *E. maximus* sample: we tested the ontogenetic allometry (covariation of size with shape during growth) and the static allometry (covariation of size with shape between individuals of the same age) with Procrustes analyses of variance (Procrustes ANOVAs; allowing the use of morphometric shape data) using the procD.lm function in the ‘geomorph’ library (Klingenberg, 2016). The intraspecific morphospaces of each bone were visualized using PCAs.

At the interspecific level, allometry can be studied between different species or clades (evolutionary allometry). Here we checked for shape variation and centroid size difference between *E. maximus* and *L. africana*, as well as for the eventual presence of an interspecific variation, using Procrustes ANOVAs (Klingenberg, 2016) on the adult sample. We tested the effect of size and robustness within the PCAs using linear regressions on the first two PCs with log(Cs), Ci and MaxL, respectively, as size estimates. Maximal length, minimum diaphyseal circumference and robustness differences between *E. maximus* and *L. africana* individuals were tested with ANOVAs on adult specimens. The morphospaces were visualized using PCAs, and theoretical shapes at the first two PCs minimum and maximum were computed in order to explore the morphological variations between the two species.

In the specific case of the undetermined femora, the ANOVAs on centroid size and shape variation were associated with pairwise comparisons (Collyer et al., 2015) in order to assess whether these bones could be distinguished into the two genera.

Finally, we compared the amount of shape variation in the six bones using the mean shapes visualizations of each sample (adults of each species, juveniles of *E. maximus*) and results of the aforementioned Procrustes ANOVAs and ANOVAs performed on shape and robustness data.

## 3 RESULTS

### 3.1 Intraspecific variation

#### 3.1.1 Ontogenetic allometry

Since there was no juvenile specimen of *L. africana* in our sample, all analyses of the shape variation during ontogeny were performed on the *E. maximus* specimens. To obtain an adult‐only sample, subadults specimens were grouped with the juvenile specimens in our analyses.

Procrustes ANOVAs on the shape data of the *E. maximus* sample indicated a significant variation of humeral and femoral shape with centroid size, that is, during growth (Table 2). Consistently, there was a significant difference of shape for the humerus and femur between non‐adult and adult specimens but not for the other bones.

**TABLE 2:** Results of Procrustes ANOVAs testing for (1) shape difference between adult and juvenile specimens and (2) correlations between shape data and log‐transformed centroid size among the *E. maximus* sample.

		Age (shape~age)	Allometry (shape~Cs)
Humerus	*n* = 18	***p* < 0.01, *r*** ^**2**^ **= 0.38**	***p* < 0.01, *r*** ^**2**^ **= 0.28**
Radius	*n* = 14	*p* = 0.70, *r* ^2^ = 0.21	*p* = 0.31, *r* ^2^ = 0.14
Ulna	*n* = 14	*p* = 0.09, *r* ^2^ = 0.29	*p* = 0.05, *r* ^2^ = 0.26
Femur	*n* = 17	***p* = 0.01, *r*** ^**2**^ **= 0.32**	***p* < 0.02, r** ^**2**^ **= 0.27**
Tibia	*n* = 13	*p* = 0.10, *r* ^2^ = 0.38	*p* = 0.17, *r* ^2^ = 0.18
Fibula	*n* = 10	*p* = 0.63, *r* ^2^ = 0.42	*p* = 0.06, *r* ^2^ = 0.44

*Note*: *r* ^2^, determination coefficient value. Significant results are in bold.

Abbreviations: Cs, Centroid size; *p*, *p*‐value.

**FIGURE 1:**
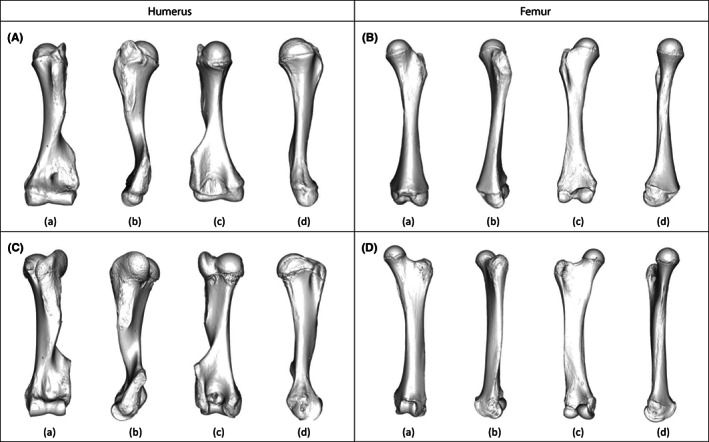
Visualizations of the mean shapes of the (A, C) humerus and (B, D) femur of (A, B) non‐adult and (C, D) adult specimens of *Elephas maximus* in (a) cranial, (b) lateral, (c) caudal, and (d) medial views.

In the Asian elephant (*Elephas maximus*), the morphological variation of the humerus during ontogeny is characterized by the development of the epiphyses from ill‐defined bulbous shapes into well‐defined structures, forming the head, the greater tubercle and the condyles. In the proximal epiphysis, the greater tubercle forms a thin crest in non‐adult specimens, then grows into a larger, wider and more rounded form (Figure 1A,C). The neck of the humerus becomes more defined, with a clear delimitation with the humeral head. Additionally, the angle formed by the humeral head and the greater tubercle widens; this is accompanied by a thickening of the humeral crest and of the deltoid tuberosity, as well as a deepening of the intertubercular groove. In the distal epiphysis, the medial and lateral condyles grow more defined with age, forming a smooth structure with clear delimitations. The olecranon fossa gets deeper, while the trochlea is also more defined on both the medial and lateral sides. The supracondylar crest appears steeper in adult specimens, forming a sharper angle with the lateral epicondyle. The proximal epiphysis grows larger during ontogeny, so that it reaches approximately the same width as the distal epiphysis during growth.

Similarly as for the humerus, the morphological variation of the femur during ontogeny is characterized by the development of both epiphyses. In the proximal epiphysis, the greater trochanter grows into a large and rounded structure under which the trochanteric fossa deepens (Figure 1B,D). The femoral neck gets proportionally thinner and longer, while the femoral head appears to retain its shape. The lesser trochanter is almost undiscernible in non‐adult specimens, and develops into a small protuberance on the medial side of the diaphysis. Additionally, the central part of the diaphysis is proportionally larger in adult specimens, closer to the width of the distal epiphysis than in non‐adult specimens. The patellar surface is not visible on the non‐adult specimens; it develops with age, forming a smooth and well‐defined articular surface on the caudal side (Figure 1B,D). On the caudal side of the distal epiphysis, both condyles are already visible in non‐adult specimens; they grow proportionally bigger and form a narrow opening on the intercondylar fossa.

#### 3.1.2 Sexual dimorphism

Sexual dimorphism could not be tested quantitatively since the sex of most specimens was unknown. In an attempt to evaluate sexual dimorphism, we computed the mean shapes of the six bones of male and female specimens of *E. maximus* when the sex groups were represented by two specimens or more, and used the unmodified 3D models of bones for which only one specimen of known sex was available. There was no adult female in the *L. africana* sample so that we could not evaluate the sexual dimorphism qualitatively. Here we describe the mean shape variation of the humerus of *E. maximus*, which was the only bone displaying a clear morphological variation pending on sex attribution.

The mean shape of the male specimens shows a massive morphology with a thick diaphysis and large epiphyses. The greater tubercle is rounded and extends as far as the humeral head proximally. The lesser tubercle is not prominent, so that the intertubercular groove forms an open angle. The trochlea is large and angled in the cranial direction, forming a marked concavity on the coronoid fossa. The mean shape of the female specimens shows a thinner shape, with narrower epiphyses. The greater tubercle is thin and extends farther than the humeral head proximally. The lesser tubercle is sharp and angled in the medial direction; the intertubercular groove forms a rounded, closed angle. The deltoid tuberosity is more prominent than in males, forming a sharper angle with the humeral crest. While the distal epiphysis is narrower than in males, the lateral condyle of the trochlea appears bigger and more elongated mediolaterally.

#### 3.1.3 Static allometry

**FIGURE 2:**
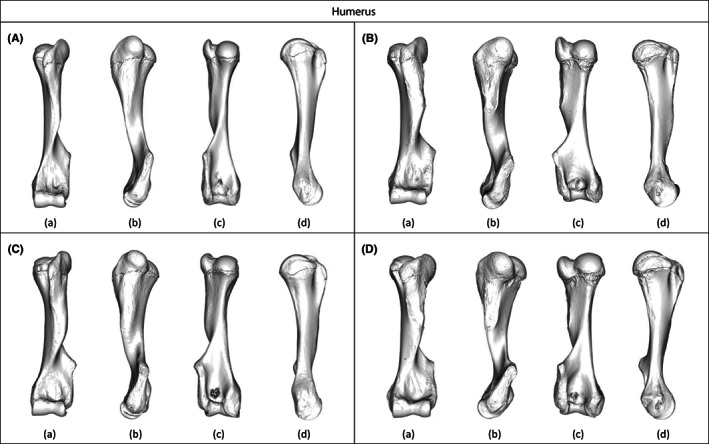
Visualizations of the humeral shapes associated with (A, B) the minimum and (C, D) the maximum of allometric regression analysis performed on adult specimens of (A, C) *Loxodonta africana* and (B, D) *Elephas maximus* in (a) cranial, (b) lateral, (c) caudal, and (d) medial views.

Results of the Procrustes ANOVA on the shape data with the centroid size as an independent variable show a significant allometry within the adult samples of *E. maximus* and *L. africana*, respectively, for the humerus only (Table 3): In the African elephant, the difference between smaller and larger adult specimens is expressed through a general thickening of the humerus in both craniocaudal and mediolateral directions, particularly visible on the deltoid tuberosity and on the supracondylar crest. In larger specimens, the greater tubercle is more rounded and extends further in the lateral direction (Figure 2). This pattern of morphological variation is similar for the humerus of the Asian elephant; with the additional difference of the supracondylar crest, which forms a larger prominence angled towards the caudal direction in larger specimens. This larger crest is associated with a deeper olecranon fossa, beginning more proximally under the supracondylar crest.

**TABLE 3:** Results of the Procrustes ANOVA testing for correlations between shape data and log‐transformed centroid size in adult specimens.

	*Elephas maximus*	*Loxodonta africana*
Humerus	***p* < 0.02, *r*** ^**2**^ **= 0.30**	***p* < 0.02, *r*** ^**2**^ **= 0.36**
Radius	*p* = 0.49, *r* ^2^ = 0.16	*p* = 0.19, *r* ^2^ = 0.54
Ulna	*p* = 0.95, *r* ^2^ = 0.10	*p* = 0.06, *r* ^2^ = 0.44
Femur	*p* = 0.25, *r* ^2^ = 0.15	*p* = 0.08, *r* ^2^ = 0.54
Tibia	*p* = 0.11, *r* ^2^ = 0.28	*p* = 0.95, *r* ^2^ = 0.24
Fibula	*p* = 0.11, *r* ^2^ = 0.15	*p* = 0.59, *r* ^2^ = 0.31

*Note*: Significant results are in bold.

Overall, larger specimens appear stouter and more robust than smaller specimens in both species, with proximal and distal epiphyses becoming similarly larger. Results of the correlation tests between the size parameters and the first two PCs of the PCAs performed on humeral shape data indicate that for both *E. maximus* and *L. africana*, the first PC is significantly correlated with size (Table 4).

**TABLE 4:** Results of the correlation tests between the size parameters and the two first principal components of the principal component analyses computed using the shape data of the adult specimens of *Elephas maximus* and *Loxodonta africana*.

		*Elephas maximus*				*Loxodonta africana*			
Bone	Component	Cs	Ci	MaxL	Rb	Cs	Ci	MaxL	Rb
Humerus	PC1	***p* = 0.017, *r*** ^**2**^ **= 0.60**	*p* = 0.44, *r* ^2^ = 0.1	***p* = 0.014, *r*** ^**2**^ **= 0.65**	*p* = 0.27, *r* ^2^ = 0.18	***p* = 0.01, *r*** ^**2**^ **= 0.8**	***p* = 0.02, *r*** ^**2**^ **= 0.71**	***p* = 0.02, *r*** ^**2**^ **= 0.78**	*p* = 0.14, *r* ^2^ = 0.46
	PC2	*p* = 0.317, *r* ^2^ = 0.18	*p* = 0.14, *r* ^2^ = 0.33	*p* = 0.35, *r* ^2^ = 0.15	*p* = 0.34, *r* ^2^ = 0.16	*p* = 0.8, *r* ^2^ = 0.01	*p* = 0.44, *r* ^2^ = 0.11	*p* = 0.7, *r* ^2^ = 0.02	*p* = 0.23, *r* ^2^ = 0.41
Radius	PC1	*p* = 0.89, *r* ^2^ < 0.01	NA	*p* = 0.78, *r* ^2^ = 0.01	NA	*p* = 0.19, *r* ^2^ = 0.94	NA	*p* = 0.23, *r* ^2^ = 92	NA
	PC2	*p* = 0.16, *r* ^2^ = 0.37	NA	*p* = 0.20, *r* ^2^ = 0.31	NA	*p* = 0.89, *r* ^2^ = 0.02	NA	*p* = 0.86, *r* ^2^ = 0.03	NA
Ulna	PC1	*p* = 0.79, *r* ^2^ = 0.01	NA	*p* = 0.84, *r* ^2^ < 0.01	NA	*p* = 0.05, *r* ^2^ = 0.68	NA	*p* = 0.09, *r* ^2^ = 0.67	NA
	PC2	*p* = 0.61, *r* ^2^ = 0.05	NA	*p* = 0.44, *r* ^2^ = 0.13	NA	*p* = 0.88, *r* ^2^ = 0.02	NA	*p* = 0.75, *r* ^2^ = 0.04	NA
Femur	PC1	*p* = 0.28, *r* ^2^ = 0.15	*p* = 0.96, *r* ^2^ < 0.001	*p* = 0.27, *r* ^2^ = 0.16	*p* = 0.15, *r* ^2^ = 0.27	*p* = 0.15, *r* ^2^ = 0.58	***p* = 0.04, *r*** ^**2**^ **= 0.75**	*p* = 0.10, *r* ^2^ = 0.65	*p* = 0.63, *r* ^2^ = 0.07
	PC2	*p* = 0.14, *r* ^2^ = 0.29	*p* = 0.30, *r* ^2^ = 0.17	*p* = 0.12, *r* ^2^ = 0.33	*p* = 0.80, *r* ^2^ < 0.01	*p* = 0.30, *r* ^2^ = 0.39	*p* = 0.38, *r* ^2^ = 0.23	*p* = 0.33, *r* ^2^ = 0.31	*p* = 0.20, *r* ^2^ = 0.43
Tibia	PC1	*p* = 0.54, *r* ^2^ = 0.09	*p* = 0.87, *r* ^2^ < 0.01	*p* = 0.53, *r* ^2^ = 0.09	*p* = 0.34, *r* ^2^ = 0.21	*p* = 0.94, *r* ^2^ < 0.001	*p* = 0.97, *r* ^2^ = 0.08	*p* = 0.94, *r* ^2^ < 0.01	*p* = 0.11, *r* ^2^ = 0.87
	PC2	***p* = 0.02, *r*** ^**2**^ **= 0.78**	*p* = 0.11, *r* ^2^ = 0.70	***p* = 0.03, *r*** ^**2**^ **= 0.70**	*p* = 0.83, *r* ^2^ < 0.01	*p* = 0.35, *r* ^2^ = 0.40	*p* = 0.43, *r* ^2^ = 0.27	*p* = 0.35, *r* ^2^ = 0.37	*p* = 0.83, *r* ^2^ = 0.20
Fibula	PC1	***p* = 0.04, *r*** ^**2**^ **= 0.66**	*p* = 0.17, *r* ^2^ = 0.38	***p* = 0.02, *r*** ^**2**^ **= 0.70**	*p* = 0.99, *r* ^2^ < 0.0001	*p* = 0.77, *r* ^2^ < 0.01	*p* = 0.54, *r* ^2^ = 0.22	*p* = 0.89, *r* ^2^ < 0.01	*p* = 0.14, *r* ^2^ = 0.68
	PC2	*p* = 0.85, *r* ^2^ < 0.01	*p* = 0.34, *r* ^2^ = 0.21	*p* = 0.93, *r* ^2^ < 0.01	*p* = 0.24, *r* ^2^ = 0.32	*p* = 0.07, *r* ^2^ = 0.96	*p* = 0.19, *r* ^2^ = 0.78	***p* = 0.02, *r*** ^**2**^ **= 0.96**	*p* = 0.53, *r* ^2^ = 0.25

*Note*: Significant results are in bold.

Abbreviations: Ci, smallest diaphyseal circumference; Cs, Centroid size; MaxL, maximum length of the bone; NA, not available since we could not measure the circumference in the radius and ulna; *p*, *p*‐value; *r* ^2^, determination coefficient value.

### 3.2 Interspecific variation

#### 3.2.1 Correlation with size and robustness variables

No correlation is detected between the different size parameters (Cs, Ci, MaxL, Rb) and the first PCs of the PCAs performed on shape data observed at the intraspecific level are not detected when using the entire adult sample (all adult *E. maximus* and *L. africana* specimens), with the exception of the minimum diaphyseal circumference along the first axis of the PCA on fibular shape data (Table 5).

**TABLE 5:** Results of the correlation tests between the size parameters and the two first principal components of the principal component analyses computed using the shape data of the entire adult sample for each bone.

Bone	Component	Cs	Ci	MaxL	Rb
Humerus	PC1	*p* = 0.60, *r* ^2^ = 0.02	*p* = 0.41, *r* ^2^ = 0.05	*p* = 0.61, *r* ^2^ = 0.01	***p* = 0.01, *r*** ^**2**^ **= 0.37**
	PC2	*p* = 0.97, *r* ^2^ < 0.01	*p* = 0.43, *r* ^2^ = 0.05	*p* = 0.96, *r* ^2^ < 0.01	*p* = 0.12, *r* ^2^ = 0.18
Radius	PC1	*p* = 0.85, *r* ^2^ < 0.01	NA	*p* = 0.96, *r* ^2^ < 0.01	NA
	PC2	*p* = 0.59, *r* ^2^ = 0.03	NA	*p* = 0.67, *r* ^2^ = 0.02	NA
Ulna	PC1	*p* = 0.05, *r* ^2^ = 0.31	NA	*p* = 0.05, *r* ^2^ = 0.31	NA
	PC2	*p* = 0.52, *r* ^2^ = 0.03	NA	*p* = 0.59, *r* ^2^ = 0.02	NA
Femur	PC1	*p* = 0.30, *r* ^2^ < 0.01	*p* = 0.37, *r* ^2^ = 0.06	*p* = 0.24, *r* ^2^ = 0.11	*p* = 0.43, *r* ^2^ = 0.05
	PC2	*p* = 0.22, *r* ^2^ = 0.11	*p* = 0.92, *r* ^2^ < 0.01	*p* = 0.37, *r* ^2^ = 0.06	*p* = 0.11, *r* ^2^ = 0.17
Tibia	PC1	*p* = 0.96, *r* ^2^ < 0.01	*p* = 0.35, *r* ^2^ = 0.11	*p* = 0.95, *r* ^2^ < 0.01	*p* = 0.33, *r* ^2^ = 0.11
	PC2	*p* = 0.89, *r* ^2^ < 0.01	*p* = 0.85, *r* ^2^ < 0.01	*p* = 0.86, *r* ^2^ < 0.01	*p* = 0.12, *r* ^2^ = 0.26
Fibula	PC1	*p* = 0.14, *r* ^2^ = 0.23	***p* = 0.04, *r*** ^**2**^ **= 0.38**	*p* = 0.11, *r* ^2^ = 0.28	*p* = 0.39, *r* ^2^ = 0.07
	PC2	*p* = 0.56, *r* ^2^ = 0.04	*p* = 0.72, *r* ^2^ = 0.02	*p* = 0.52, *r* ^2^ = 0.05	*p* = 0.91, *r* ^2^ < 0.01

*Note*: Ci, smallest diaphyseal circumference; Cs, Centroid size; MaxL, maximum length of the bone; NA, not available since we could not measure the circumference in the radius and ulna; *p*, *p*‐value; *r* ^2^, determination coefficient value. Significant results are in bold.

Although Procrustes ANOVAs testing the covariation of shape data with log‐transformed centroid size within the *E. maximus* sample detected an allometry in the humerus, no allometry was not detected when testing the entire adult sample (all adults *E. maximus* and *L. africana* specimens). All the following analyses are thus performed without checking for covariation with the centroid size.

#### 3.2.2 Size and robustness analyses

There was no significant difference in the centroid size, the circumference nor the length of the bones between *E. maximus* and *L. africana* (Table 6). Results of the *t* tests on the robustness of the bones (Rb) indicated that *E. maximus* displayed a significantly more robust humerus than *L. africana* (Table 6, Figure S7a, S8a). Although the *t* tests indicated no significant difference, qualitative comparisons of the mean shape of the humerus, ulna and tibia revealed considerably more robust bones in *E. maximus* than in *L. africana*. Scatterplots of the length on circumference ratios were consistent with the qualitative observations: *E. maximus* displayed higher Ci/MaxL ratios for each bone, that is, a higher robustness (Figure S7b–d, S8b–d). The single *L. cyclotis* was included in the scatterplots, and for each bone displayed a higher robustness than *E. maximus*.

**TABLE 6:** Results of ANOVAs testing for size and shape variation between the species.

	Cs	Ci	MaxL	Rb	Shape
Humerus	*p* = 0.25, *r* ^2^ = 0.20	*p* = 0.99, *r* ^2^ < 0.01	*p* = 0.16, *r* ^2^ = 0.16	***p* < 0.02, *r*** ^**2**^ **= 0.40**	***p* = 0.001, *r*** ^**2**^ **= 0.26**
Radius	*p* = 0.86, *r* ^*2*^ = 0.02	NA	*p* = 0.68, *r* ^*2*^ = 0.02	NA	*p* = 0.30, *r* ^*2*^ = 0.11
Ulna	*p* = 0.35, *r* ^*2*^ = 0.17	NA	*p* = 0.25, *r* ^*2*^ = 0.12	NA	***p* = 0.02, *r*** ^**2**^ **= 0.22**
Femur	*p* = 0.21, *r* ^2^ = 0.21	*p* = 0.7, *r* ^2^ = 0.07	*p* = 0.11, *r* ^2^ = 0.20	*p* = 0.15, *r* ^2^ = 0.15	*p* = 0.16, *r* ^2^ = 0.11
Tibia	*p* = 0.71, *r* ^2^ = 0.09	*p* = 0.99, *r* ^2^ < 0.01	*p* = 0.45, *r* ^2^ = 0.07	*p* = 0.09, *r* ^2^ = 0.37	***p* = 0.002, *r*** ^**2**^ **= 0.22**
Fibula	*p* = 0.40, *r* ^2^ = 0.20	*p* = 0.78, *r* ^2^ = 0.07	*p* = 0.50, *r* ^2^ = 0.16	*p* = 0.17, *r* ^2^ = 0.34	*p* = 0.63, *r* ^2^ = 0.17

*Note*: Ci, smallest diaphyseal circumference; Cs, Centroid size; MaxL, maximum length of the bone; Rb, robustness; NA, not available since we could not measure the circumference in the radius and ulna; *p*, *p*‐value; *r* ^2^, determination coefficient value. Significant results are in bold.

#### 3.2.3 Shape analyses

##### Humerus

Results of the Procrustes ANOVA on the humerus shape data (Table 6) revealed a significant difference of shape between the two species (*p* = 0.001, *r* ^2^ = 0.26). The neighbour‐joining tree computed on adult humeral shape data confirmed a clear separation between specimens of *E. maximus* and *L. africana*, with the *L. cyclotis* specimen placed in the middle of the *E. maximus* group (Figure S9a).

**FIGURE 3:**
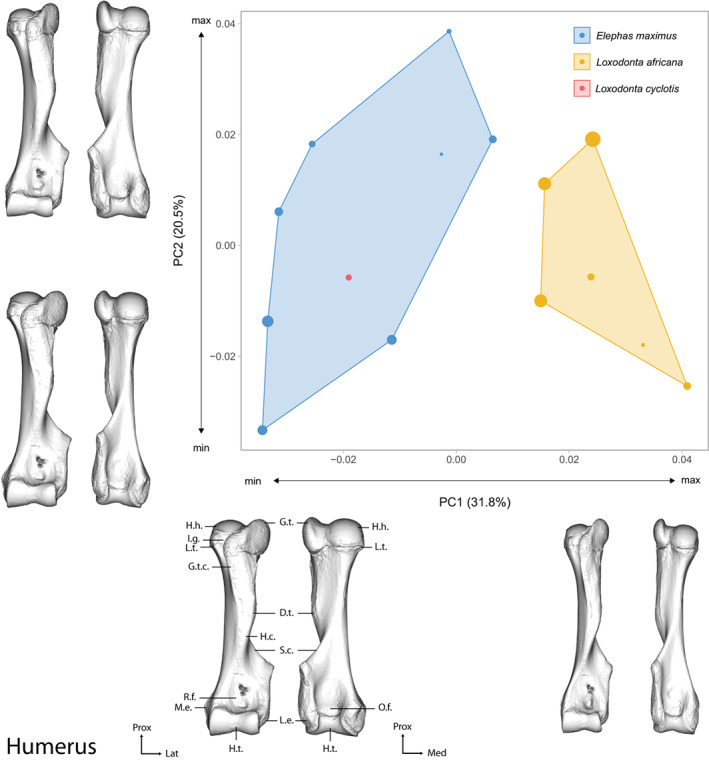
Results of the PCA performed on morphometric data of the humerus of all adult specimens along with the visualizations of the theoretical shapes at the minimum and maximum of the first two axes. The size of the points is proportional to the centroid size of the bones. D.t., deltoid tuberosity, G.t., greater trochanter, G.t.c., greater trochanter crest, H.c., humeral crest, H.h., humeral head, H.n., humeral neck, H.t., humeral trochlea, I.g., intertubercular groove, L.e., lateral epicondyle, L.t., lesser trochanter, M.e., medial epicondyle, O.f., olecranon fossa, R.f., radial fossa, S.c., supracondylar crest; Lat., lateral, Med., medial, Prox., proximal. See Figure S10 for anatomical details of the humerus.

The first two axes of the PCA performed on the humerus shape data express 52.3% of the global variance (Figure 3). The first axis (which represents 31.8% of the variance) separates the African savanna elephant on the positive part and the Asian and African forest elephants on the negative part of the graph. The theoretical shape at the PC1 minimum shows a massive and stout morphology, with wide epiphyses and a thick diaphysis, while the theoretical shape at the PC1 maximum shows a thin and elongated morphology, with epiphyses extended in the craniocaudal axis and overall less pronounced protuberances. Both *L. africana* and *E. maximus* display an important intraspecific variation along the second axis (20.5% of the variance). For *L. africana*, the intraspecific variation is expressed by the first two axes and appears to be linked to the centroid size of the specimens, with the smallest ones driving the variation towards the positive part of the first axis and the negative part of the second axis. For *E. maximus*, the biggest specimens appear to drive the variation towards the negative part of the two first axes. The specimens of *E. maximus* closest to the *L. africana* group are not the larger ones; size thus does not appear to drive the variation similarly for the two species along the first axis. The specimen of *L. cyclotis* is part of the *E. maximus* group. Detailed descriptions of the theoretical shapes at the PCs minimum and maximum are in Results S1.

On this PCA, the female of *E. maximus* is closer to the male specimen of *L. africana* than to the males of their own species. For *E. maximus*, the only fully adult female specimen is at the extreme positive part of the hull on the first axis, while the three male specimens are at the extreme negative part. The first axis displays a gradient, from male *E. maximus*, to female *E. maximus*, then male *L. africana* and finally female *L. africana*. The mean shapes of each species logically follow the gradient observed along the first axis: *L. africana* displays a thin, elongated morphology, as opposed to the stout and massive one displayed by *E. maximus*.

Taking non‐adult specimens into account, we observe a wide distribution of *E. maximus* along the second axis (PC2 = 20.22% of the variance), mainly driven by the two juvenile specimens in the negative part of the graph; the only subadult specimen of *E. maximus* is placed closer to the *L. africana* cluster along both axes (Figure S10). This distribution is confirmed by the neighbour‐joining tree on the humeral shape data of the entire sample (Figure S9b).

##### Radius

Results of the Procrustes ANOVA on the radial shape data (Table 6) revealed no significant difference of shape between *E. maximus* and *L. africana* (*p* = 0.30, *r* ^2^ = 0.11). The neighbour‐joining tree computed on radial shape data showed a clear separation between *E. maximus* and *L. africana*, with the *L. cyclotis* specimen placed closer to a *L. africana* specimen than to the rest of the sample (Figure S9c).

The first two axes of the PCA performed on the radius shape data express 50.3% of the global variance (Figure S12). *E. maximus* displays a high intraspecific variation, occupying most of the PCA graph, while all *L. africana* specimen are grouped in the middle of the first axis and in the negative part of the second axis (PC2: 21.7% of the variance). Most of the *L. africana* distribution overlaps with that of *E. maximus*, but not with *L. cyclotis*.

##### Ulna

Results of the Procrustes ANOVA on the ulna shape data (Table 6) revealed a significant difference of shape between *E. maximus* and *L. africana* (*p* = 0.02, *r* ^2^ = 0.22). The neighbour‐joining tree computed on ulnar shape data showed a slight separation between *E. maximus* and *L. africana*, with the *L. cyclotis* specimen placed closer to the *E. maximus* specimens (Figure S9d).

**FIGURE 4:**
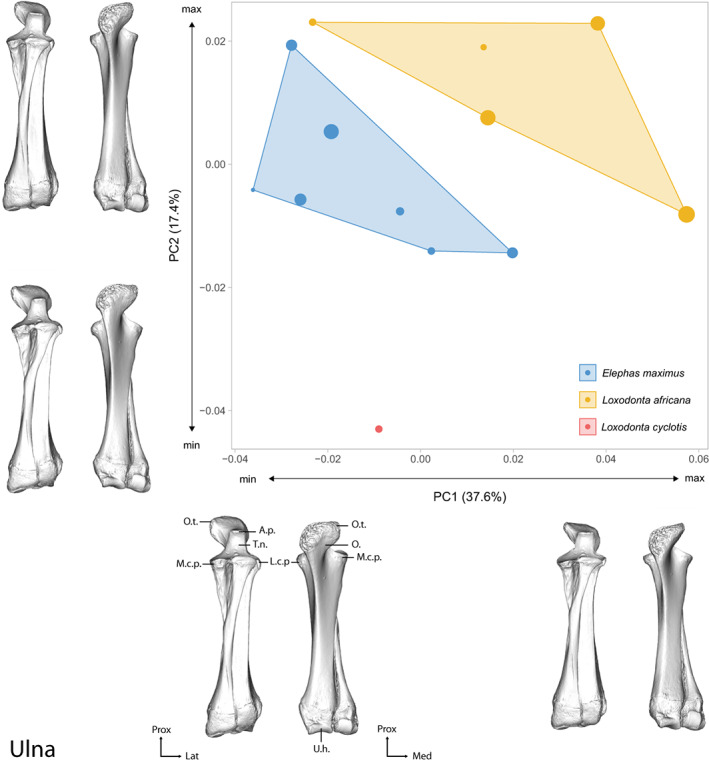
Results of the PCA performed on morphometric data of the ulna of all adult specimens along with the visualizations of the theoretical shapes at the minimum and maximum of the first two axes. The size of the points is proportional to the centroid size of the bones. A.p., anconeal process, A.s.r, articular surface for the radius, L.c.p., lateral coronoid process, M.c.p., medial coronoid process, O., olecranon, O.t., olecranon tuberosity, T.n., trochlear notch, R.no., radial notch, S.p.u., styloid process of the ulna, U.h., ulnar head; Lat., lateral, Med., medial, Prox., proximal. See Figures S12 and S13 for anatomical details of the ulna.

The first two axes of the PCA performed on the ulna shape data express 54.01% of the global variance (Figure 4). While being clearly distinct on the graph, both *E. maximus* and *L. africana* display a large intraspecific variation along the first (PC1: 37.6% of the variance) and second (PC2: 17.4% of the variance) axes. The first axis appears to be linked with the size of the specimens: larger specimens of *L. africana* are situated in the positive part of the graph, while larger specimens of *E. maximus* are in the negative part of the graph. The second axis separates *L. cyclotis*, in the negative part of the graph, from the two other species on the positive part. Detailed descriptions of the theoretical shapes at the PCs minimum and maximum are in Results S1.

##### Femur

Results of the Procrustes ANOVA on the femur shape data (Table 6) revealed no significant difference of shape between *E. maximus* and *L. africana* (*p* = 0.16, *r* ^2^ = 0.11). The neighbour‐joining trees computed on humeral shape data showed no clear separation between *E. maximus* and *L. africana*, whether considering the undetermined specimens or not (Figure S9e,f).

There was also no significant difference in shape when taking the undetermined adult specimens into account and considering them as *E. maximus* specimens (*p* = 0.13, *r* ^2^ = 0.08). The PCA performed on the shape data of this sample indicates that these specimens are closer to the *E. maximus* group, with almost no overlap, than to the *L. africana* group and the *L. cyclotis* specimen, supporting the hypothesis of this subsample belonging to the Asian elephant species (Figure S15).

However, when considering the undetermined adult specimens as a third group, we found a significant difference in shape (*p* = 0.02, *r* ^2^ = 0.20): pairwise comparisons indicate that while *E. maximus* and *L. africana* did not differ significantly in their femoral shape (*p* = 0.22), the undetermined group did differ significantly from both *E. maximus* (*p* = 0.02) and *L. africana* (*p* = 0.04). Details of the femur anatomy are in Figure S16.

The k‐NN algorithm reached 66.7% of correct classification when predicting the three groups (*E. maximus*, *L. africana*, undetermined specimens), and up to 81% when considering the undetermined specimens as *E. maximus*.

##### Tibia

Results of the Procrustes ANOVA on the tibial shape data (Table 6) revealed a significant difference of shape between *E. maximus* and *L. africana* (*p* < 0.01, *r* ^2^ = 0.22). The neighbour‐joining tree computed on tibial shape data showed a clear separation between *E. maximus* and *L. africana*, with the *L. cyclotis* specimen placed closer to *E. maximus* (Figure S9g).

**FIGURE 5:**
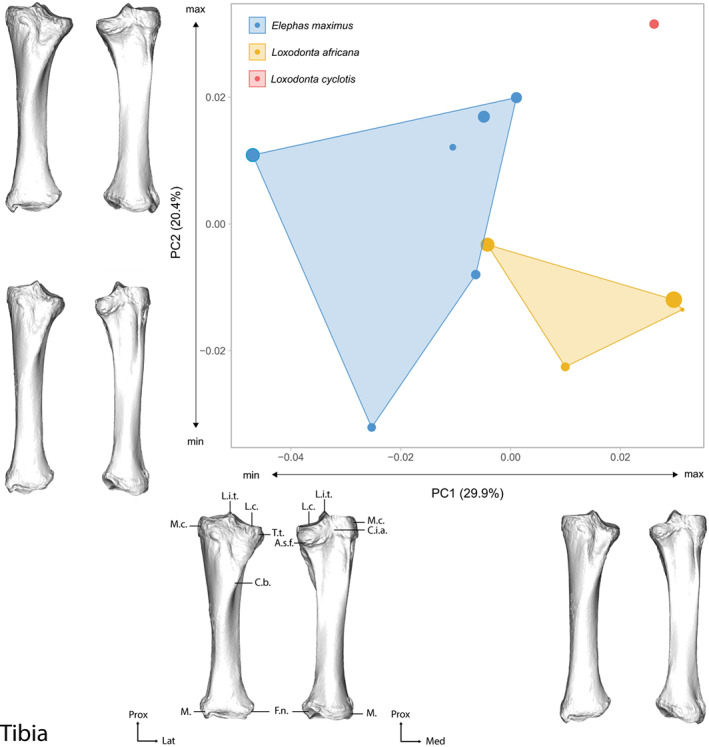
Results of the PCA performed on morphometric data of the tibia of all adult specimens along with the visualizations of the theoretical shapes at the minimum and maximum of the first two axes. The size of the points is proportional to the centroid size of the bones. (a) A.s.f., articular surface for the fibula, (c) C.b., cranial border, C.i.a., caudal intercondular area, Fi.n., fibular notch, M., malleolus, M.c., medial condyle, L.c., lateral condyle, L.i.t., lateral intercondylar tubercle, (t) T.cr., tibial crest, T.t., tibial tuberosity; Lat., lateral, Med., medial, Prox., proximal. See Figure S16 for anatomical details of the tibia.

The first two axes of the PCA performed on the tibia shape data express 50.30% of the global variance (Figure 5). The first axis (which represents 29.9% of the variance) separates both *Loxodonta* species in the positive part of the graph, and *E. maximus* in the negative part. The theoretical shape at the PC1 minimum shows a massive form, with wide epiphyses and a thick diaphysis. At the opposite, the theoretical shape at the PC1 maximum shows a more delicate morphology, with a thinner diaphysis in both mediolateral and craniocaudal axes. The *L. cyclotis* specimen is clearly separated from the *L. africana* group on the second axis (PC2: 20.4% of the variance). This axis appears to be linked with the centroid size of the specimens: for both *E. maximus* and *L. africana*, the larger specimens are closer to the positive part of the graph than the smaller ones. The theoretical shape at the PC2 minimum shows a thin and elongated morphology, the diaphysis and the epiphyses being reduced in both the craniocaudal and the lateromedial axes; The theoretical shape at the PC2 maximum shows a more massive morphology, with wide epiphyses and a large diaphysis.

The mean shapes of each species roughly correspond to the differences observed along the first axis: *L. africana* displays a thinner, elongated morphology, as opposed to the stout and massive one displayed by *E. maximus*. Detailed descriptions of the theoretical shapes at the PCs minimum and maximum are in Results S1.

##### Fibula

Results of the Procrustes ANOVA on the fibula shape data (Table 6) revealed no significant difference of shape between *E. maximus* and *L. africana* (*p* = 0.63, *r* ^2^ = 0.17). The neighbour‐joining tree computed on fibular shape data showed no clear separation between the two species, with the *L. cyclotis* specimen placed in the middle of the NJ tree (Figure S9h). Details of the fibula anatomy are in Figure S18.

### 3.3 Integrative overview

At the intraspecific level (*Elephas maximus*), only the stylopod bones showed an ontogenetic allometry. In both the humerus and femur, this allometry was characterized by a development of the proximal extremity of the bone, while the distal extremity stayed relatively similar in shape. ANOVAs testing for shape difference between male and female specimens of *E. maximus* yielded no significant difference for any of the six bones; however, qualitative comparisons of the mean shapes of male and female specimens suggested morphological variations in the humeral epiphyses. ANOVAs testing for static allometry among the adult samples detected a significant allometry for the humerus only: in both species, the humerus grows more massive and robust with increased centroid size. Robustness analyses also revealed a significant difference for the humerus only: *E. maximus* displays more robust humeri than does *L. africana*. While there was no significant difference for the other bones, qualitative comparisons of the mean shapes indicated globally more robust bones in *E. maximus* than in *L. africana*. ANOVAs testing for shape difference at the interspecific level revealed significant differences for the humerus, the ulna and the tibia. For each of the three bones, the morphological variation was noticeable enough to allow for species distinction based on qualitative analysis alone. Overall, the humerus is the bone showing the most variation of shape between specimens, whether at the intraspecific or interspecific level, followed by the ulna and tibia displaying clear morphological differences between the two species, whereas the femur and fibula display almost no morphological variation.

## 4 DISCUSSION

### 4.1 Morphological variation at the intraspecific level

#### 4.1.1 Shape variation during ontogeny

Since long bones play a prominent role in the support and movement of the body, their external morphology is expected to reflect the biomechanical demands they face (Iwaniuk et al., 1999, 2000); among those, body mass in particular is a major parameter (Biewener, 1989; Hildebrand, 1982). During ontogeny, bones are thus subjected to increasing stresses, although to varying degrees depending on the considered taxa. Gracility can be defined as the inverse of robustness, that is, as the ratio of the entire bone length over the diaphyseal circumference. The gracility of bones increases during growth (considered here as positive allometry) in most taxa, with the notable exception of proboscideans (Carrier, 1996). More specifically, cursorial taxa and taxa under 20 kg display a positive allometry, while graviportal taxa (including rhinos and hippos) display a negative allometry during ontogeny (Carrano, 1999; Christiansen, 2002; Kilbourne & Makovicky, 2012). We found a negative allometry in the variation of the stylopod bones during growth in *E. maximus*, partly supporting the results of Kokshenev and Christiansen (2010) stating that both *Elephas maximus* and *Loxodonta africana* share similar negative allometric patterns in their six long bones during ontogeny, growing more robust with increasing size. Here, a clear shape difference is thus observed between adult and non‐adult specimens in the humerus and femur, but not for the radius, ulna, tibia and fibula, which indicates an isometric growth pattern for these bones. Thus, our results are also partly consistent with those of Kilbourne and Makovicky (2012), who studied the tibia, femur and humerus, and found an isometric growth pattern for these bones: our results are consistent with theirs regarding the isometric growth of the tibia, but not for the femur and the humerus.

Most interestingly, while the limb long bones of elephants do not share the allometric trend observed in most quadrupeds, bones of their autopods do: most bones of the manus and pes display an isometry or a positive allometric pattern in Asian elephants (Main & Biewener, 2004; Miller et al., 2008). This variation in allometric patterns highlights how differences in functional constraints (e.g. in relation to the position within the limb, proximity to the footpad) between limb long bones and bones of the autopod might affect how they respond to mass increase during growth.

The visible effect of ontogeny on the stylopod but not on the zeugopod might be linked to the anatomical position of the bones within the limb, and thus the different structural strains they face: the zeugopod bones are ‘more columnar’ (i.e. positioned more orthogonally to the ground) than the stylopod bones (Larramendi, 2015), so we can hypothesize that they are more parallel to the weight and ground reaction forces. Their shape would thus be primarily adapted to these forces, and thus need to be stay stable during ontogeny. In the stylopod bones, the proximal epiphysis develops proportionally more than the distal epiphysis during growth, roughly doubling its width. This difference in shape variation along the proximo‐distal axis is consistent with the idea of a distal part of the limb more adapted to weight bearing in an orthogonal position to the ground: we found that the distal epiphyses, part of the elbow and knee joints respectively, display a more stable shape through ontogeny. In elephants, these joints play a specific role in supporting the body mass by distributing the load on the entirety of the articular surface (Weissengruber, Egger, et al., 2006; Weissengruber, Fuss, et al., 2006). Even the youngest elephants display this pattern; since elephant calves weight around 90 to 100 kg at birth, we can propose that it allows them to withstand a high body mass from the earlier stages of life. The shape variation of the proximal epiphyses during growth suggests that the proximal part of the stylopods is not similarly adapted to weight bearing in young individuals. Kilbourne and Makovicky (2012) suggested that a larger sample size might reveal isometric growth pattern in bones of the stylopod. However, the general trend here indicates a clear allometric growth pattern in both the femur and the humerus, consistent with the results of Kokshenev and Christiansen (2010); as such, an isometric growth pattern in the stylopod bones of elephants would be very surprising.

#### 4.1.2 Static allometry and sexual dimorphism

We observed a global pattern of increased robustness (i.e. ratio of circumference to bone length) at the intraspecific level in both *E. maximus* and *L. africana*, with larger specimens being more robust than smaller specimens. This is consistent with what is generally observed in heavier mammalian clades, in which an increase in body size and mass is generally associated with a global broadening of the limb long bones, with an enlargement of both the diaphysis and epiphyses (Bertram & Biewener, 1990, 1992; Christiansen, 1999; Kilbourne & Makovicky, 2012; Mallet et al., 2019).

Despite the qualitative observations of increased robustness in all the bones, the shape variation within the adult sample of each species indicated a significant negative allometry in the humerus only: larger specimens exhibited stout and robust humeri while their smaller counterparts exhibited more gracile and elongated ones; this is consistent with the fact that the humerus is the bone displaying the most shape variation linked to mass increase in our analyses. In quadruped mammals, the centre of mass is typically closer to the forelimb than to the hindlimb, so that forelimb elements bear more weight than hindlimb ones (Etienne et al., 2021; Hildebrand, 1974; Lessertisseur & Saban, 1967; Polly, 2007). Elephants are no exception to this rule (Ren et al., 2010), their forelimb supporting around 60% of the total weight (Henderson, 2006). The ground reaction forces as well as the weight‐bearing forces are thus higher in the forelimb than in the hindlimb, increasing the mechanical load on the bones and on their associated shape variation; this would explain the presence of a negative allometry in the forelimb only. The negative allometry observed in the humerus only is consistent with previous works stating that the effect of a high body mass would be more pronounced on the stylopod than on the zeugopod (Biewener, 1989; Campione & Evans, 2012; Mallet et al., 2019).

In elephants, body size and mass can vary considerably depending on the sex of the animal, so that we might expect to observe more robust bones in the larger, heavier male specimens. Among the specimens for which the sex was known, we observed more robust humeri in male specimens, which displayed wider epiphyses than females. This shape variation might be directly linked to differences in body mass and body mass distribution between the sexes: since weight is expected to scale with linear dimensions cubed, even a small increase in body height results in a large variation in mass. On average, male African savanna elephants display 3.2 m height at the shoulder, which is about half a metre more than their female counterparts; as a result, they can weight more than twice their body mass (Wilson et al., 2011). These size and mass differences between males and females are similar in Asian elephants (Wilson et al., 2011). Additionally, males can grow tusks in both species. Female Asian elephants do not grow tusks (Sukumar, 1989), and although both sexes can have tusks in *L. africana*, they are generally bigger in males (Elder, 1970; Smith & Fisher, 2013). As a result, there might be a big difference in weight distribution between male and female specimens (albeit more pronounced in *E. maximus* than in *L. africana*). Since we observed this robustness variation between males and females in the humerus only, we could propose that the humerus plays a role in the accommodation of the increased weight of the head linked to the presence of heavier tusks and a generally higher body mass supported by the forelimb. However, the subsample of specimens for which the sex was known is too small to state on the sexual dimorphism in bone robustness, and a larger sample might reveal shape and robustness difference in limb bones other than the humerus, or conversely, show that there is not consistent variation linked to sex in the external morphology of the bones.

### 4.2 Morphological variation at the interspecific level

#### 4.2.1 Adaptation to weight bearing

While still gigantic compared to the majority of quadrupedal mammals, the Asian elephant is smaller in height and body mass than its African savanna counterpart (Wilson et al., 2011). However, at equal shoulder height the two species do not differ in weight (Larramendi, 2015). In our sample, we found no evidence for a statistically significant size difference in the length of the limb long bones between the two species. This could arguably be due to a small sample size, or the missing information regarding the sex of our specimens, leading to a biased sample and an overlapping of male Asian elephants and female African elephants, closer in size and mass. In any case, the absence of species‐specific size difference in our sample, as well as the similar variance in the size distribution of the two species indicate that in our sample all the adult specimens of both *L. africana* and *E. maximus* share the same approximate height, so that we can assume that our sample is composed of specimens sharing globally similar body masses. This enables us to investigate how the two species adapt to a similar body weight.

We observed a clear morphological variation in the long bones of the two species: *L. africana* typically has long and gracile bones, when *E. maximus* exhibits stouter, more robust ones. While this global shape difference is qualitatively considerable for all bones with the exception of the fibula, we found that only three bones displayed a significant shape difference: the humerus, ulna and tibia; this is consistent which our qualitative observations in which they displayed a greater variation than the other bones. The humerus of the Asian elephant was overall enlarged in both craniocaudal and mediolateral directions, with larger epiphyses. In the African savanna elephant, both the shaft and the epiphyses appeared narrower, with a greater tubercle considerably elongated in the proximal direction. The greater tubercle bears the insertions of the supraspinatus and infraspinatus muscles, which play a prominent role in the shoulder joint stabilization, as well as a role in humeral abduction. This might indicate different adaptations to weight support in the two species, with the Asian elephant relying on enlarged elbow and shoulder joints to distribute the mechanical load. This may be linked to differences in skull size and relative position between Asian and African elephants: the skull of *E. maximus* is indeed relatively larger and positioned higher than in African species (Larramendi, 2015; Marchant & Shoshani, 2007), resulting in a stronger mechanical load on the forelimb. However, male *E. maximus* specimens display smaller (and thus lighter) tusks than do *L. africana* specimens, and female *E. maximus* specimens do not have tusks, so that the supposedly relative increased weight of the head in *E. maximus* might be counterbalanced by the reduced/absent tusks, compared to *L. africana*. The relative mass of the head compared to the body is thus difficult to ascertain. Despite this possible balance between the two species (heavier tusks vs. higher and heavier skull), it is worth noting that Marchant and Shoshani (2007) described an additional muscle in the neck of *E. maximus* (m. *splenius superficialis*), interpreted as an additional muscular support of the weight of the head. Larramendi (2015) hypothesized that this muscle was heavily involved in the support of the head and was present in all *Elephas* species as well as in several other extinct proboscideans. The secondary loss of this muscle in *L. africana* may be linked to the reduced size of the cranial dome, on which it inserts, although it is uncertain which led to the other. This difference in the neck musculature between *Elephas* and *Loxodonta* indicates that the two species adapted in a different manner to support the weight of the head; it is thus logical to expect other accompanying anatomical differences, as observed here in the humerus. However, quantitative biomechanical comparisons of the weight distribution between the two species, taking tusks weight into consideration, are needed to better understand how head weight and head position relative to the body may influence the shape of limb long bones.

In our shape analyses of the humerus, both species were clearly separated, and we observed a distribution linked with size. However, the size distribution was almost opposite for the two species, indicating different growth patterns (Figure 3). While we found a significant static allometry in both species, the interspecific variation was stronger, again indicating different adaptations of the humeral shape to size increase. Female Asian elephants, more gracile, were closer to the males of the African elephant than to the males of their own species in our shape analysis; the two species were separated in the morphospace, with male and female specimens positioned at the extremities, indicating a possible gradient ranging from the more robust bones (male *E. maximus* specimens) to the more gracile ones (female *L. africana* specimens). However, this morphological distribution linked to sex stays hypothetical, as the sex of most specimens was unknown.

The role of the radius in weight support has been highlighted among a wide sample of quadrupedal mammals (Bertram & Biewener, 1992), including heavy taxa such as rhinoceroses (Etienne et al., 2021; Mallet et al., 2019) and hippopotamuses (Fisher et al., 2007). Proboscideans are an exception to this pattern: in elephants, the ulna plays a more important role in the support of the body mass than the radius, which is reduced in size (Smuts & Bezuidenhout, 1993). The restricted role of the radius in body mass support could explain the absence of a morphological variation between Asian and African savanna elephants.

In elephants, the columnarity of the forelimb is partly achieved by the reorientation of the trochlear notch in the dorsal direction (Christiansen, 1999). The ulna is parallel to the weight and ground reaction forces during static weight bearing, and as such allows an efficient support and distribution of the mechanical load to the humerus. The main extensor of the forearm is the triceps brachii, which inserts on the extremity of the olecranon (Fisher et al., 2007; Barone, 2020). In heavier taxa, the olecranon is wider and longer, especially in the anteroposterior direction, which corresponds with the increased strain exerted by this muscle to maintain an erect posture (Etienne et al., 2021). Etienne et al. (2021) stated that a longer olecranon relative to the length of the ulna, as well as a more posterior position of the olecranon, would allow a more open angle when the elbow is in extension, as well as a longer lever arm. Here, we found that the olecranon was thin and elongated in the craniocaudal direction in the African savanna elephant, whereas it was rounder and wider in the mediolateral direction in the Asian elephant. This might indicate a higher stress exerted by the long head of the triceps brachii (the most powerful part of the muscle) in the African elephant, opposed to a higher strain exerted by the lateral and medial heads (accessories to the long head, inserting on the medial and lateral sides of the humerus) in the Asian elephant. These muscular insertions could play a role proximally in the global thickening of the diaphysis we observed in the humerus of the Asian elephant. We observed no clear difference in the relative length of the olecranon between the Asian and African savanna elephant species, indicating that they do not differ in elbow position nor in lever arm efficiency.

We found no significant variation of shape between the femur of the *E. maximus* and *L. africana*. In quadrupedal mammals, the forelimb and the hindlimb ensure different function in locomotion: the forelimb plays an additional role in braking during locomotion, while the hindlimb plays a prominent role in the propulsion of the body (Dutto et al., 2006). As a result, we expect them to react differently to increases in body mass, as it was shown in rhinos (Etienne et al., 2021; Mallet et al., 2019). But, while the functional distribution of weight bearing is similar in elephants (Panagiotopoulou et al., 2012; Schmidt‐Burbach & Eulenberger, 2008), it is not the case for the locomotor functions: Ren et al. (2010) compared the elephant forelimb and hindlimb to a four wheeled vehicle, in which the propulsion and braking roles are equally shared by the limbs. Since the forelimb bears more weight than the hindlimb, we infer that the bones of the hindlimb are subjected to less stress than bones of the forelimb, so that they would be less prone to morphological variation; however, the considerable shape variations observed in the tibia suggest that weight constraints vary greatly between the stylopod and the zeugopod. Since we found no difference between the shape of the femur between the two species, we were not able to diagnose the undetermined femora. They appeared closer to the Asian species than to the African ones in the shape analyses, although there was no clear differentiation. These bones might belong to the African forest elephant; conversely, their reduced size might indicate that they all belong to female specimens, in which case their distribution (slight separation on the NJ tree) might indicate a sexual dimorphism in the femur of *E. maximus*.

The tibia is the main weight bearer in the hindlimb zeugopod, due to its large surface of articulation with the bones of the autopod, and its orthogonal position to the ground. We found that the hyperverticality of the hindlimb was reflected in the shape of the tibia that in elephants is distinctly different from that of other quadrupedal mammals, even when compared with heavier taxa (Barone, 2020; Etienne et al., 2021; Smuts & Bezuidenhout, 1994). A particular feature is the markedly concave articular surface of the tibia, corresponding with the femoral condyles. This translates to a higher congruence of the knee joint, allowing the weight to be distributed more efficiently onto the femur (Weissengruber, Egger, et al., 2006; Weissengruber, Fuss, et al., 2006). Bertram and Biewener (1992) noted a decrease in the tibial curvature associated with an increase in body mass among terrestrial mammals. This is particularly visible in our sample since the shaft is straight in all specimens, although it is more pronounced in *L. africana* specimens. While they both share a morphology adapted to the near‐columnarity of the limb, we observed a clear difference in the tibial global shape of the two species.

In *E. maximus*, the tibia was stouter and more massive than in *L. africana*, with thicker condyles in the proximal epiphysis. The lateral condyle in particular was wider along the dorsoventral axis, and was elongated in the caudal direction. This condyle bears the insertion of muscles involved in the extension of the hip and of the knee, as well as the abduction and the external rotation of the ankle. These enlarged areas for muscles involved in joint flexion and rotation suggest a higher compliance in *E. maximus*, which is consistent with the higher limb compliance in the elbow and knee of the Asian elephant as compared to the African savanna elephant described by Kokshenev and Christiansen (2010) based on limb bone scaling. In elephants, the large, prominent tibial crest bears the insertion of the *biceps femoris* muscle, a powerful extensor of the hip and knee. This muscle originates on the ischium, preventing its elevation under the effect of body weight, and contributes to keeping the pelvis upright (Barone, 2020; Shindo & Mori, 1956b), so that its enlarged area of attachment suggests a high muscular strain, which is consistent with the need to counterbalance the massive weight of the animal. In both *Loxodonta* an*d Elephas*, the tibial crest is prominent and placed more medially than in most taxa, delimitating a wide, concave surface on the cranial side, and ends distally in a rough area for muscular attachment (Smuts & Bezuidenhout, 1994). This cranial, concave area provide a wide zone of insertions for patellar ligaments, which are the continuation of the various heads of the *quadriceps femoris* muscle, a powerful knee extensor, allowing the stabilizing of the knee. Additionally, one of the heads of the *quadriceps femoris* muscle inserts via a separate tendon onto the tibial tuberosity instead of stopping on the patella (Weissengruber, Egger, et al., 2006; Weissengruber, Fuss, et al.,&nbsp;^2006^). This is not the case in other heavy taxa such as rhinos (Etienne et al., 2021) and hippos (Fisher et al., 2010); we conclude that this tibial crest development is linked to a high muscular strain, and thus that this adaptation is specific to weight support in elephants by increasing the knee‐joint stability.

*E. maximus* displayed wider epiphyses than *L. africana*, with an enlarged tibial crest. This mediolateral widening was associated with a larger concave area on the cranial side. This area forms a triangle, delimited proximally by the condyles, laterally by the tibial crest and medially by a rough ridge connecting the distal limit of the tibial crest and the most cranial part of the medial condyle. Several muscles and ligaments involved in hip adduction and ankle flexion insert in this area. Under those, several digital flexors insert directly on the concave area (Shindo & Mori, 1956b). In *E. maximus*, this area reached more distally on the shaft of the tibia, indicating relatively larger areas of muscular attachment. This might indicate that the Asian elephant relies more than the African savanna elephant on the stabilizing power of the hindlimb muscles to maintain an erect posture.

#### 4.2.2 Changes in robustness

Several studies have investigated bone robustness in proboscidean limb bones, with sometimes contradicting results: based on linear measurements, Christiansen (2007) originally stated that there was no difference in robustness between the stylopod bones of *E. maximus* and *L. africana*, but in a later study (also based on linear measurements) Kokshenev & Christiansen, 2010 found that the six bones were significantly more robust in Asian elephants than in African savanna elephants. Our results are consistent with the latter: although the interspecific robustness difference was significant for the humerus only, all the other bones displayed a similar trend that might prove significant based on a greater sample.

Various trends of robustness can be observed among terrestrial quadrupeds, with equally various explanations as to their biomechanical consequences. Numerous attempts to formulate generally applicable allometric laws using body mass, bone length and bone circumference have been proposed, and have been subject to several decades of debates (Alexander, 1977; Alexander et al., 1979; Biewener, 1983, 2005; Bertram & Biewener, 1990; Christiansen, 1999, 2002, 2007, Kokshenev et al., 2003; Kokshenev, 2007; McMahon, 1973, 1975a, 1975b). Using three of the most common allometric models, Kokshenev and Christiansen (2010) concluded that the bones of the Asian elephant, more robust, were more adapted to resist the bending and torsion forces exerted by the muscles (bending‐torsion model), while the bones of the African savanna elephant, more gracile, were optimized to resist gravitational forces (buckling model). However, this raises the question of the underlying causes behind this species‐specific adaptation.

This morphological divergence between *E. maximus* and *L. africana* could be linked with human activity: Asian elephants have a long history of being used by humans for various tasks, ranging from field work and military use to modern day tourism and circus shows. However, despite this extensive human exploitation, elephants were never domesticated, so that elephant breeding was never fully controlled by humans. We can thus exclude the possibility of an anthropic selection towards more robust individuals, or of a by‐product of domestication. Another explanation could be a difference in locomotor mode between the two species: there is a widely spread belief that African savanna elephant can reach higher running speed than Asian elephants. Several studies reported high speeds in African savanna elephants (Andrews, 1937; Garland, 1983; Iriarte‐Díaz, 2002; Rue, 1994), that were then attributed to high error rates in measurements taken from automobile speedometers, as well as human error due the excitement of witnessing a charging wild elephant (Hutchinson et al., 2006). As such, they consider these values to be exaggerations, and based on their own measurements and predictive models state that both elephant species can reach the same maximal speed.

In addition to sharing the same running speed, both species share a similar walking gait (Langman et al., 2012; Ren et al., 2010), so that we conclude that the robustness difference does not result from a variation in speed nor gait. Asian and African savanna elephants share similar locomotor mechanics (Hutchinson et al., 2006; Ren et al., 2008), and the differences in their foot anatomy are minor (Miller et al., 2008); additionally, the ground reaction forces are distributed similarly in the footpad of both species (Panagiotopoulou et al., 2016). These results suggest that there is no postural difference that could explain the robustness variation.

However, *E. maximus* and *L. africana* occur in different types of habitat, so that they walk on different types of substrates and terrains. Asian elephants mostly walk on soft and yielding surfaces in humid forests and jungles, while African savanna elephants roams on savanna grasslands and sandy plains, as well as the hard, dry surfaces of semiarid deserts (Roocroft & Oosterhuis, 2000; Wilson et al., 2011). For obvious practical reasons, all studies performed on elephant gait, running speed and weight distribution have been conducted on hard and artificial surfaces; we could hypothesize that while they found no difference in force distribution nor in locomotion patterns, the results might have been different if the analyses had been conducted in the natural habitat of the animals. Asian elephants might require more stabilizing while walking on uneven and soft ground, as well as greater dexterity when navigating in densely forested areas. Furthermore, *E. maximus* and *L. africana* have evolved different foraging habits: African savanna elephants are browsers, using their trunk rather than their feet when foraging. Asian elephants, however, use their forefeet to scrape and dig deep into the soil (Roocroft & Oosterhuis, 2000), and have been reported to use their forelimbs to secure fallen trees and tear away at the tree bark and root system, or to strike down bamboo and tall grass (Buckley, 2000). This larger range of limb movement observed in *E. maximus* may also result in higher muscular strains and thus explain the stouter morphology we observed in the humerus.

#### 4.2.3 What about the African forest elephant?

African forest elephants are the smallest of the three living species. Our sample comprised the six bones of a single specimen, presenting no sign of ageing or pathology; we included them in our shape analyses in order to see how it compared to the other species. The results are intriguing, as this specimen was clearly separated from the other species when looking at the ulna and tibia, but was included in the *E. maximus* cluster for the humerus and radius, and in the *L. africana* cluster for the femur. The fibula was also clearly separated from those of the two other species; however, this result is to be considered with caution since this bone yielded minimal taxonomic signal in our study. Qualitative observations of the six bones of the *L. cyclotis* specimen are consistent with these results: overall *L. cyclotis* displayed a stouter morphology than *L. africana*, with bones of the forelimb displaying a shape closer to those of the Asian elephant, while bones of the hindlimb displayed more ‘in‐between’ shapes. The specimens to which *L. cyclotis* is closest for the bones of the forelimb are not the same than for bones of the hindlimb; since bones from the same ‘closest’ specimens were represented in analyses on both the forelimb and the hindlimb, the shape similarity of *L. cyclotis* with one species or the other depending on the bone is not due to a change in sample. Consistently, analyses on a juvenile specimen of *L. cyclotis* showed a similar pattern of morphological similarity (C.B., pers. obs.): the humerus and ulna were closest to those of the juvenile Asian elephants, while the tibia was separated from those of both *E. maximus* and *L. africana*. The surprising pattern of shape similarity between bones of the adult *L. cyclotis* specimens and those of *E. maximus* and *L. africana* might thus be representative of this species.

Most interestingly, the interspecific morphological variation we describe here in modern elephants differs from other extant graviportal mammals such as rhinos. Body mass and habitat vary greatly across rhinoceros's species (Wilson et al., 2011), the various limb bones are differently affected. In rhinoceroses, the shape variation of the humerus and the femur is mostly driven by the phylogenetic signal, while it is the radius and ulna that are mainly affected by body mass (Mallet et al., 2019). Conversely, we find here a pattern of shape variation linked to the type of limb (forelimb vs. hindlimb) rather than to the limb segment (stylopod vs. zeugopod). The entire forelimb of *L. cyclotis* is morphologically closer to that of *E. maximus*, suggesting, if this specimen is indeed representative of this species, that in elephants, the forelimb bones' morphology could be more influenced by body mass (smaller in *L. cyclotis* and *E. maximus* than in *L. africana*) and the environment (forest vs. open plains) than by the phylogenetic proximity, while it is the opposite for the hindlimb. This suggests that the adaptation of the limb bones to a high body mass does not happen in the same manner across the various ‘graviportal’ taxa.

## 5 CONCLUSION

In both species, we observed an ontogenetic allometry in the stylopod bones due to a large growth in size of the proximal epiphyses as compared to the distal ones; this suggests that the elbow and knee joints are adapted to withstand massive weight from the earliest ontogenetic stage. The other bones follow an isometric growth pattern, indicating that the bones of the zeugopod react differently to an increase in mass. We also observed an allometry among adult specimens: bigger (and thus heavier) specimens displayed stouter, more robust bones. While this allometry was significant for the humerus only, the same trend was observed in the other bones. Limb long bones robustness thus increases with weight. While these intraspecific variations are clearly defined, their signal is masked by the more pronounced differences between the two species: our shape analyses revealed significant differences in the external morphology of the humerus, ulna and tibia between *E. maximus* and *L. africana*: the humerus is stouter in the Asian elephant, presenting enlarged area for the attachment of muscles involved in shoulder joint stabilization and humeral abduction, indicating different adaptations to weight support in both species. The ulna, which plays an important role in the support of the body mass, displays a difference in shape and orientation of the olecranon, allowing for a wider angle of limb extension and a more efficient lever arm in *E. maximus* than in *L. africana*. The tibia displays a morphology adapted to the limb hyperverticality in both species; however, the condyle bearing muscles involved in hip and knee extension, as well as in ankle abduction and rotation, were elongated in *E. maximus*, indicating a higher limb compliance in the knee of Asian elephants. Additionally, the tibia displays enlarged muscular insertion zones for muscles involved in knee and hip stabilization, suggesting that the Asian elephant relies more than the African savanna elephant on the stabilizing power of the hindlimb muscles to maintain an erect posture. These morphological variations are strongly pronounced, allowing for species distinction based on the external shape of the humerus, the ulna and the tibia. While the difference in robustness was significant in the humerus only, our qualitative comparisons indicated an overall higher robustness in *E. maximus* than in *L. africana*. Since both species share similar walking speed and gait, these parameters do not explain this variation. However, Asian and African savanna elephants live in highly different habitats, so that the robustness difference might be linked to their walking substrate (hard and dry vs. soft, humid soil) and direct environment (open plains vs. closed forest), since navigating through the humid forests would require more stabilizing and dexterity than walking in the savanna. We also suggest that the overall robustness variation between *E. maximus* and *L. africana* is linked to their locomotor and foraging habits, since the two species also exhibit different foraging behaviours, Asian elephants being able to make raking motions with their feet, displaying a higher forelimb dexterity than African savanna elephants, which do not use their forelimbs to feed.

## AUTHOR CONTRIBUTIONS

A.H. and C.B. designed the study. A.D., A.H. and C.B. did the bone data acquisition. C.B. conducted the analyses and drafted the manuscript, A.H. and C.B. contributed to the final manuscript, and all authors read it and approved it.

## CONFLICT OF INTEREST STATEMENT

The authors declare no conflict of interest.

## Supporting information


Figure S1:

Figure S2:

Figure S3:

Figure S4:

Figure S5:

Figure S6:

Figure S7:

Figure S8:

Figure S9:

Figure S10:

Figure S11:

Figure S12:

Figure S13:

Figure S14:

Figure S15:

Figure S16:

Figure S17:

Figure S18:

Data S1.

Tables S1–S6.

## Data Availability

The R scripts used to perform the analyses will be made available on Github. 3D models provided by the Idaho Museum of Natural History can be found on www.MorphoSource.org with the following links: ark:/87602/m4/M168768 (IMNH 1486, humerus); ark:/87602/m4/M168870 (IMNH 1486, radius & ulna); ark:/87602/m4/M168758 (IMNH 1486, femur); ark:/87602/m4/M168938 (IMNH 1486, tibia); ark:/87602/m4/M168762 (IMNH 1486, fibula). 3D models obtained from MNHN specimens will be made available on www.3dtheque.mnhn.fr. Most of the remaining models used in this study will be made available in their respective museum repositories and/or by curators, and unless otherwise decided, deposited on MorphoSource.
