# Supplementary figures and images for: Shape variation in the limb long bones of modern elephants reveals adaptations to body mass and habitat
Source: J Anat. 2023 Feb 23;242(5):806–30. doi: 10.1111/joa.13827 (PMC10093169; doi:10.1111/joa.13827)

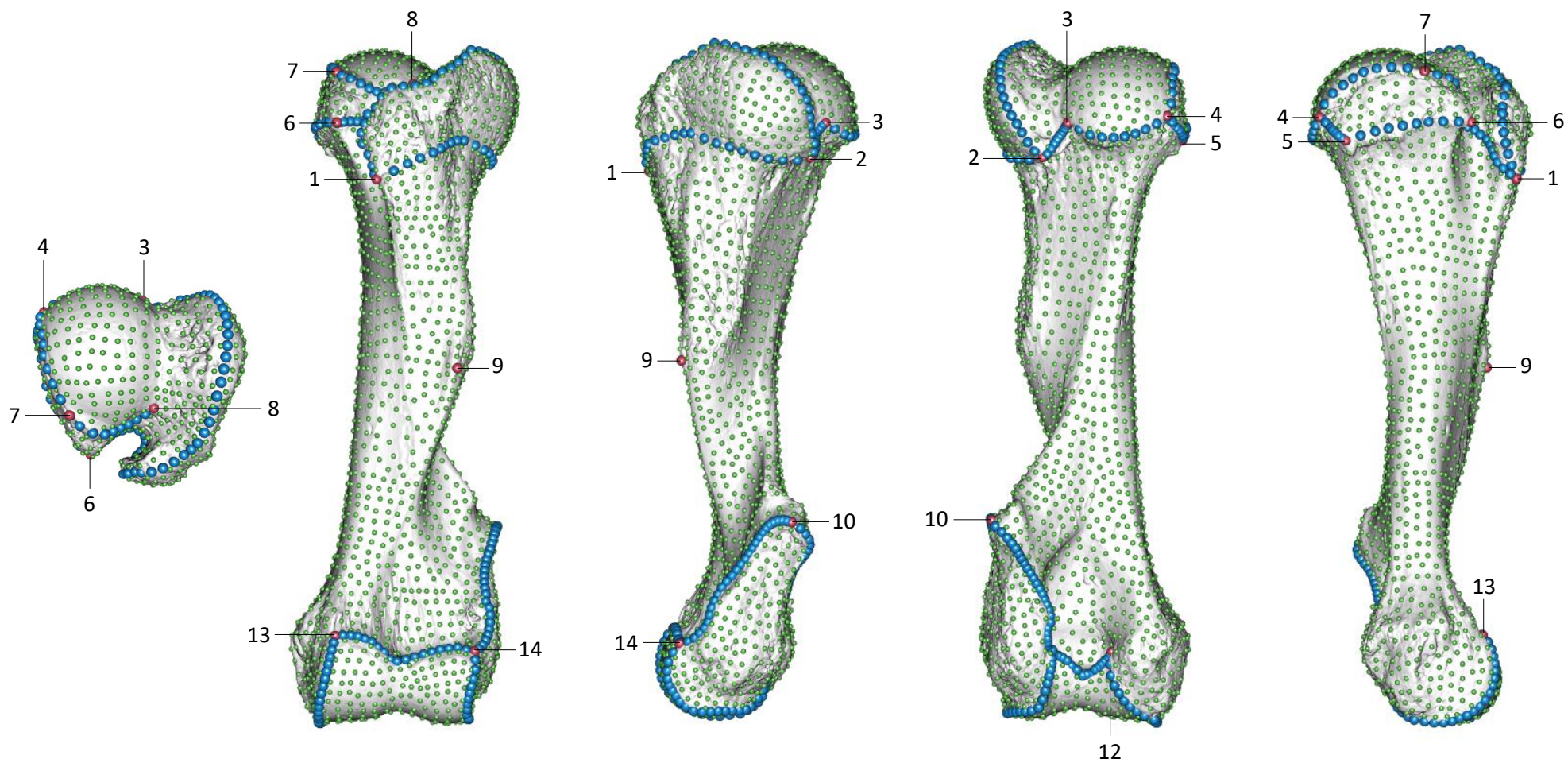

Supplement: Supplementary file 1 — Figure S1: [file JOA-242-806-s020.pdf]

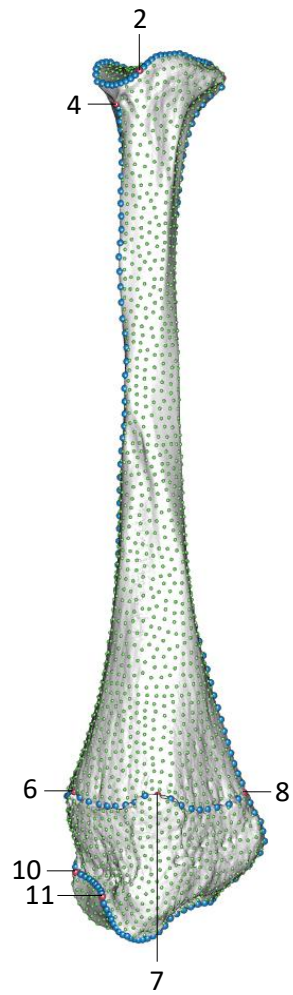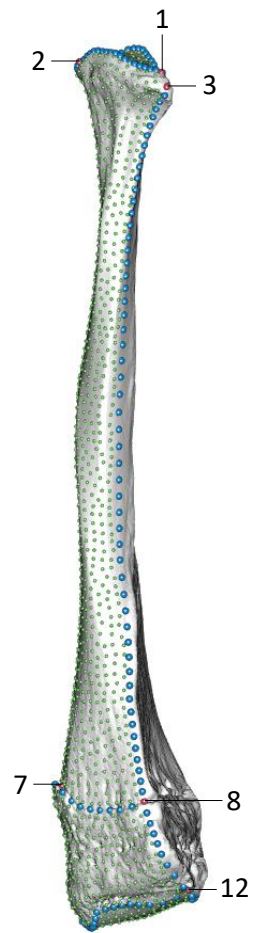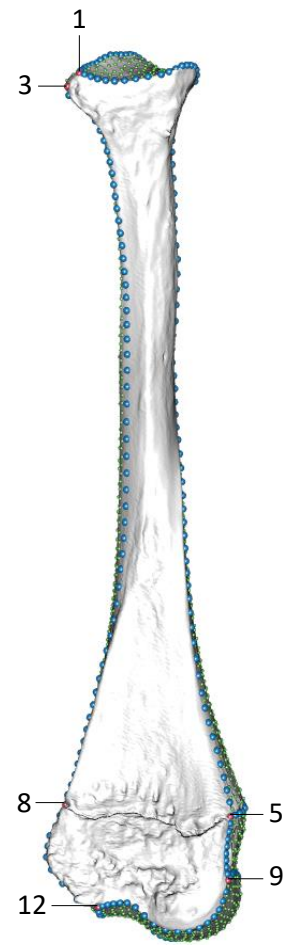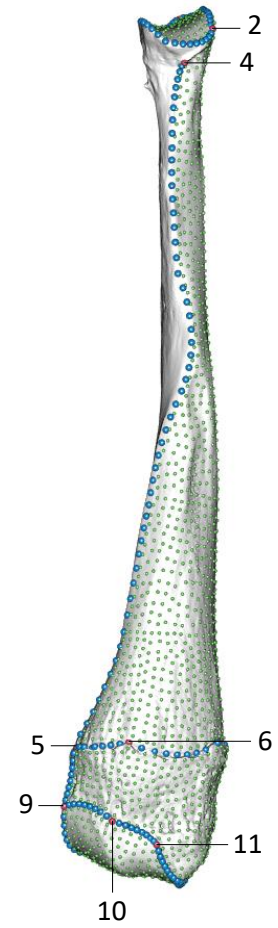

Supplement: Supplementary file 2 — Figure S2: [file JOA-242-806-s015.pdf]

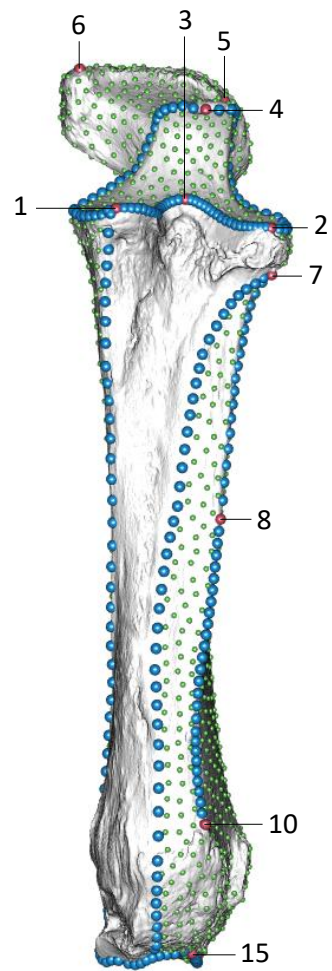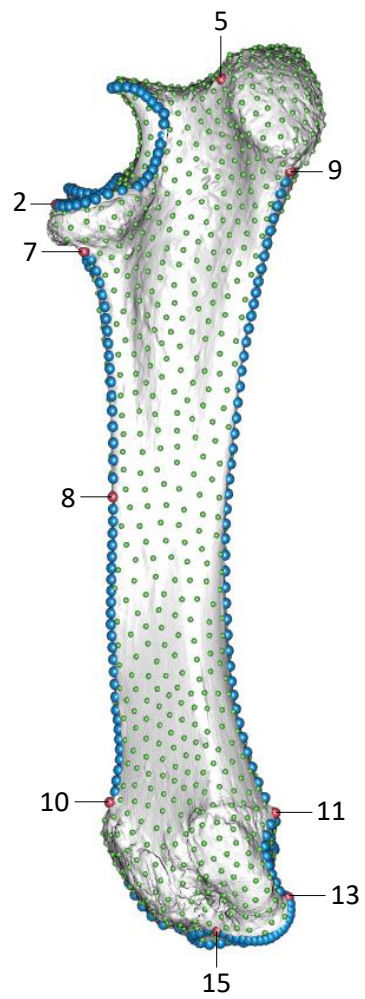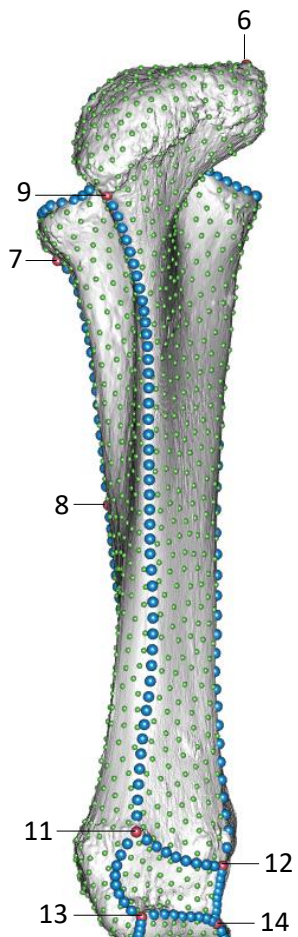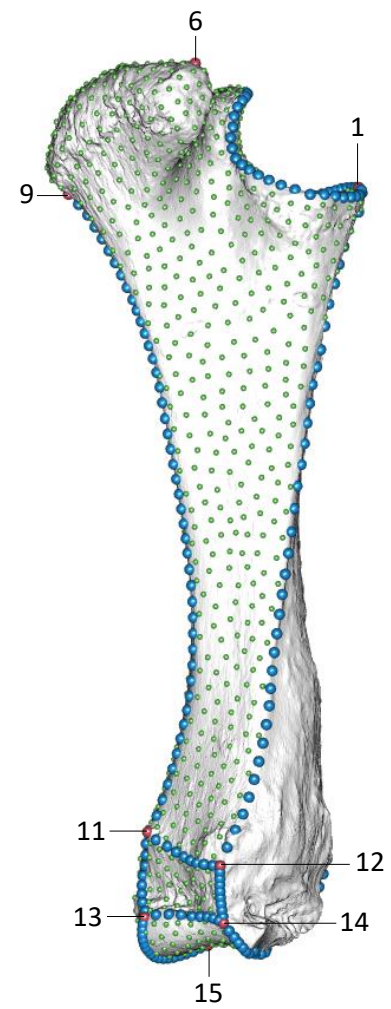

Supplement: Supplementary file 3 — Figure S3: [file JOA-242-806-s012.pdf]

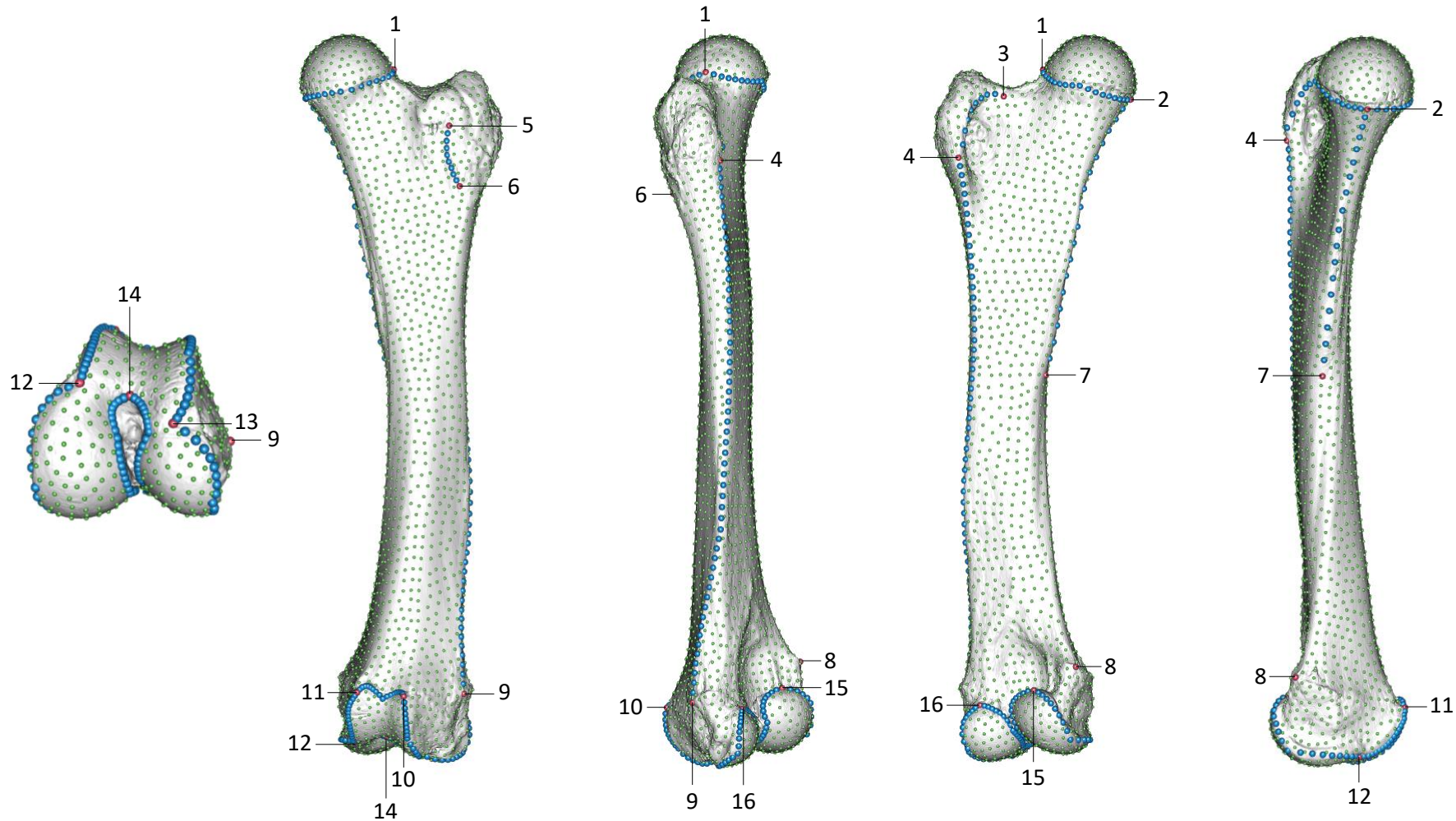

Supplement: Supplementary file 4 — Figure S4: [file JOA-242-806-s010.pdf]

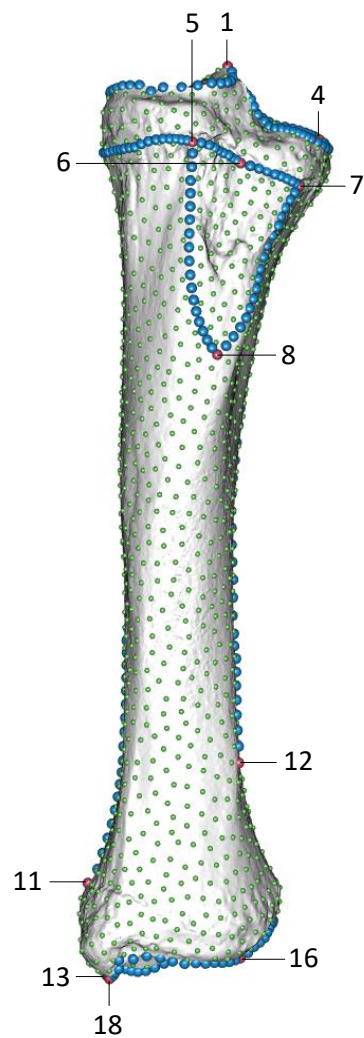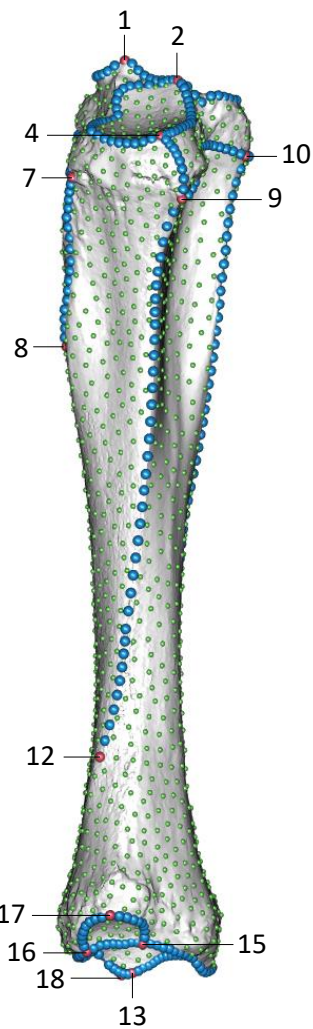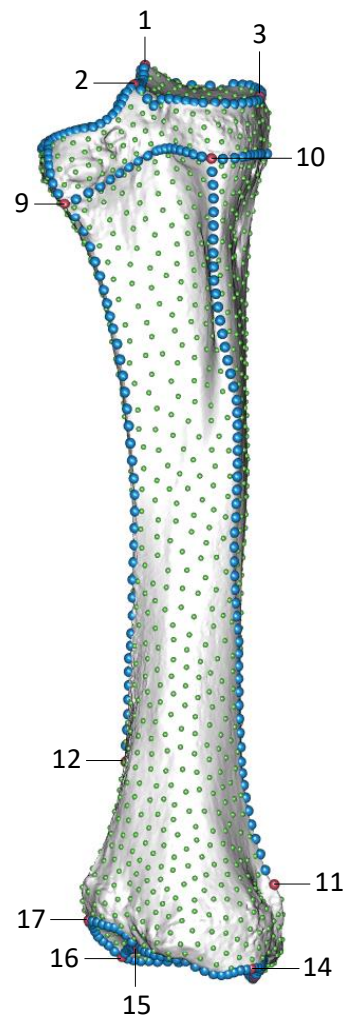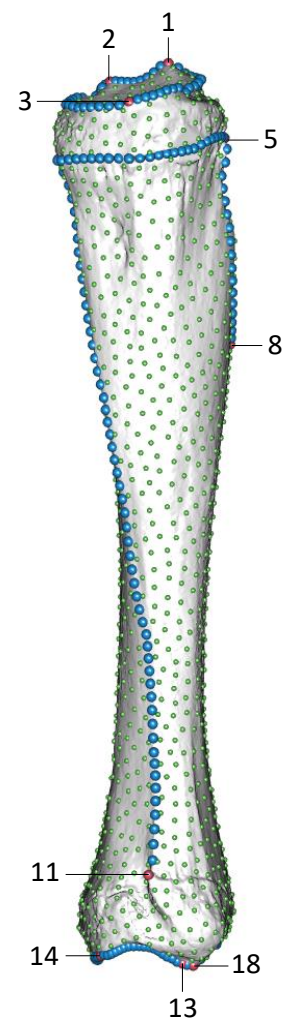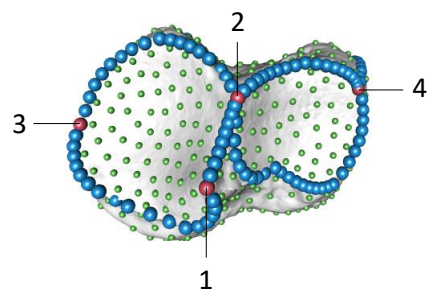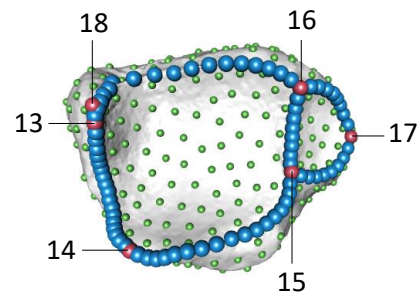

Supplement: Supplementary file 5 — Figure S5: [file JOA-242-806-s004.pdf]

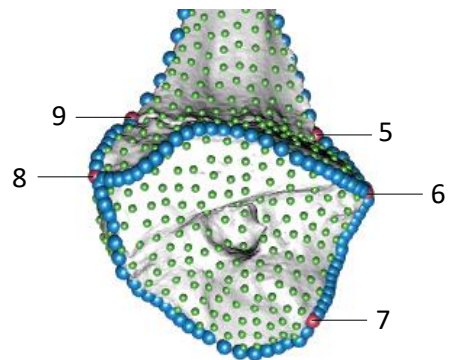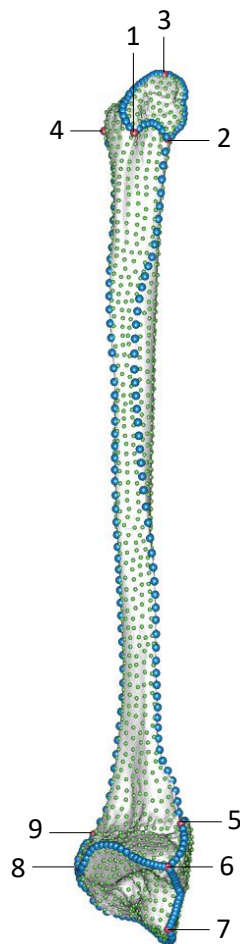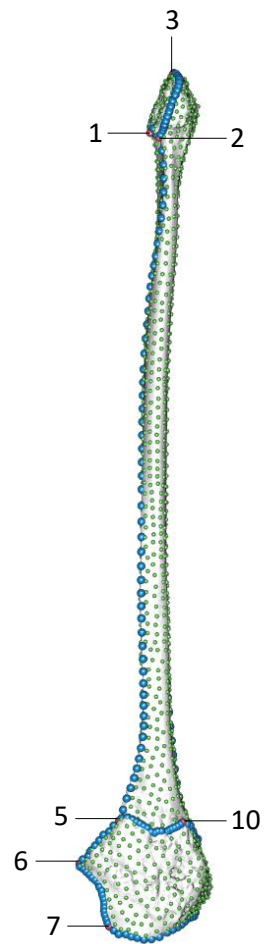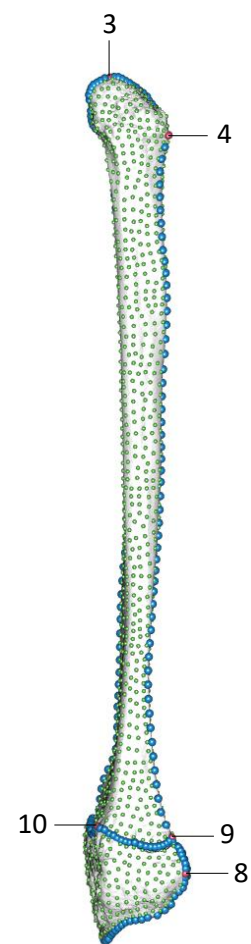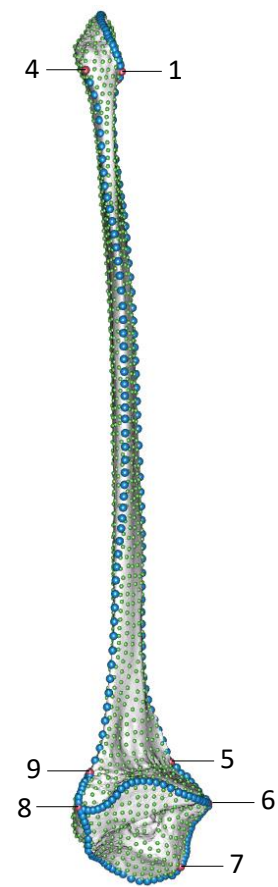

Supplement: Supplementary file 6 — Figure S6: [file JOA-242-806-s018.pdf]

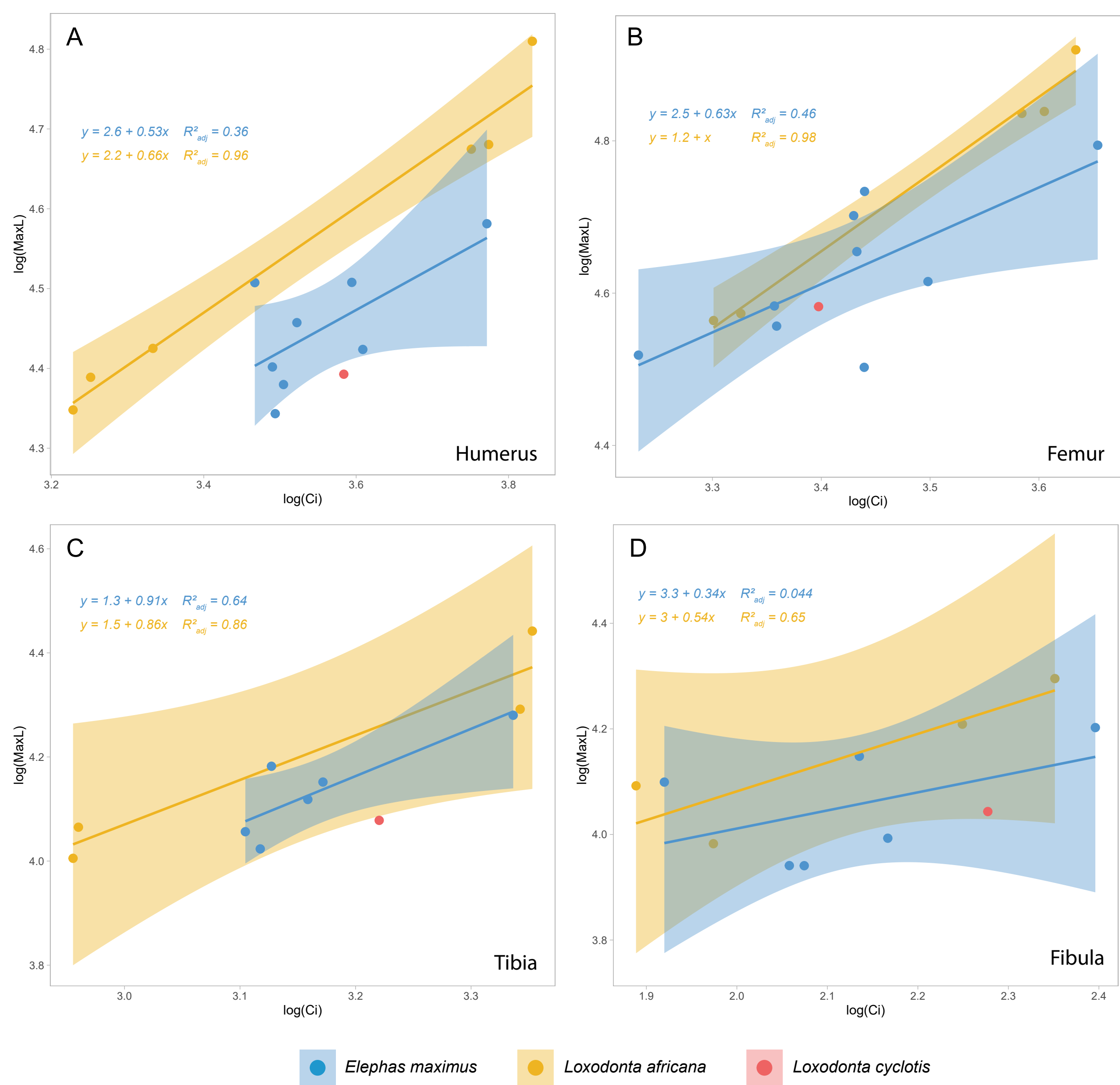

Supplement: Supplementary file 7 — Figure S7: [file JOA-242-806-s008.pdf]

Humerus

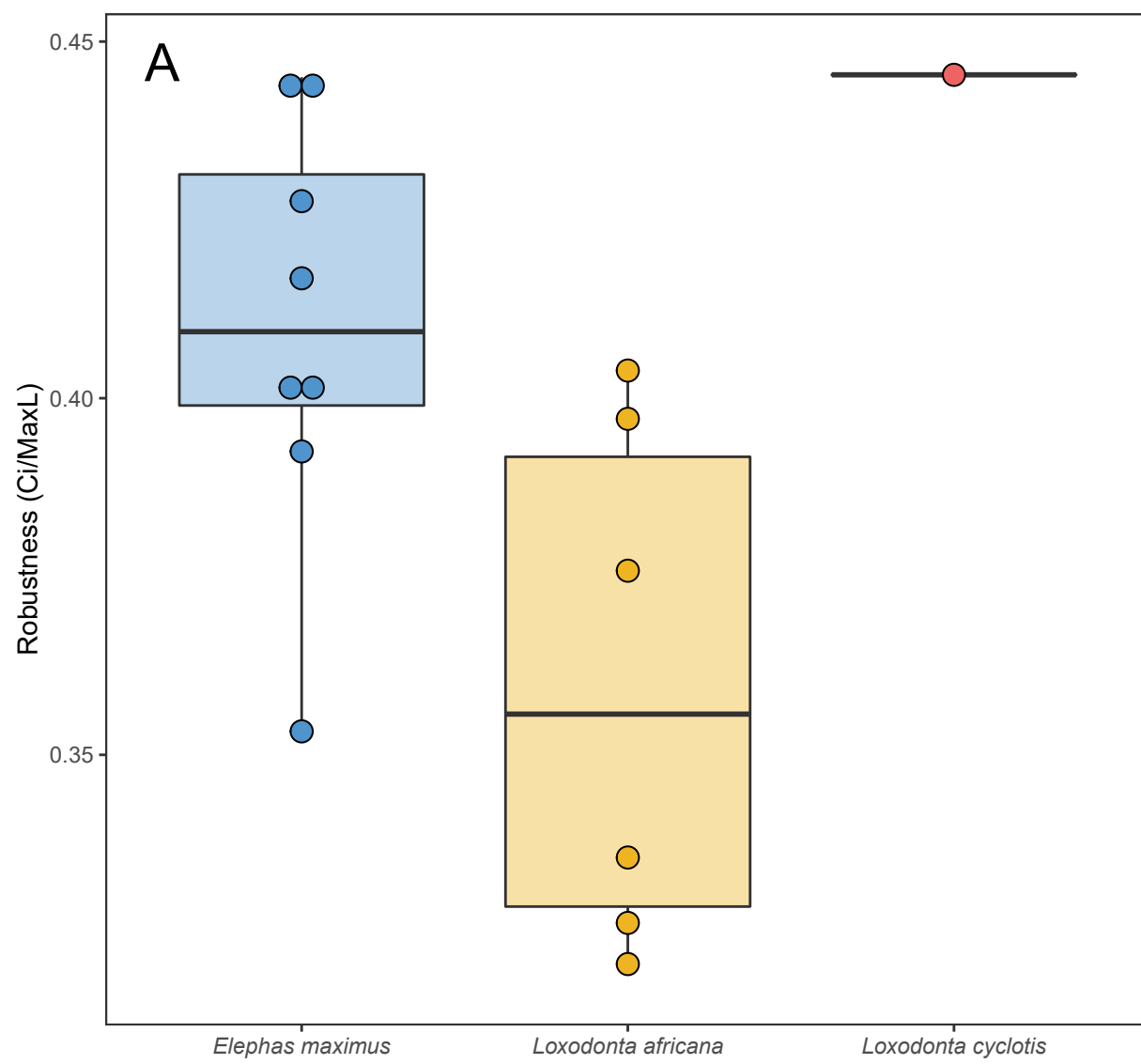

Femur

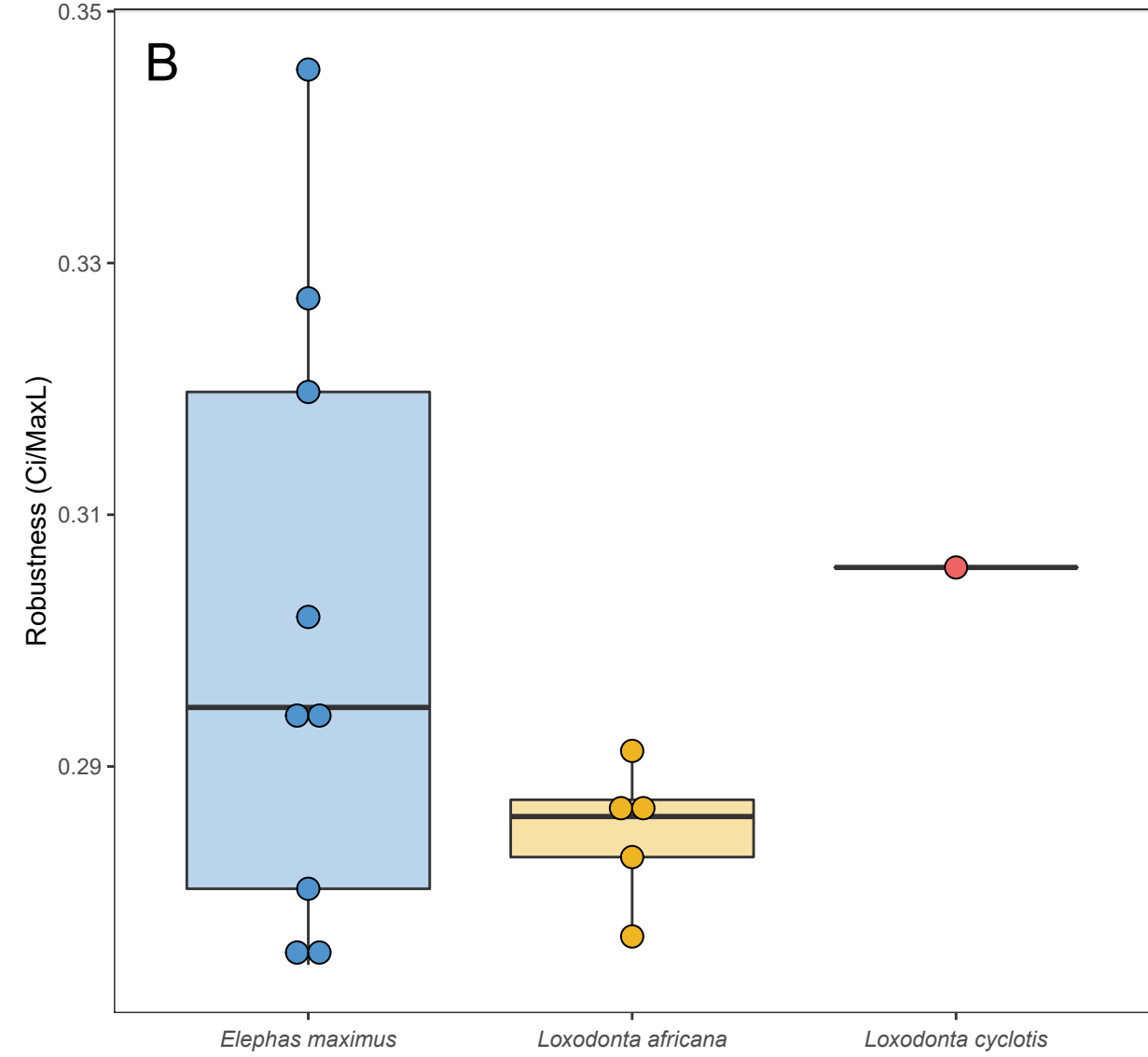

Tibia

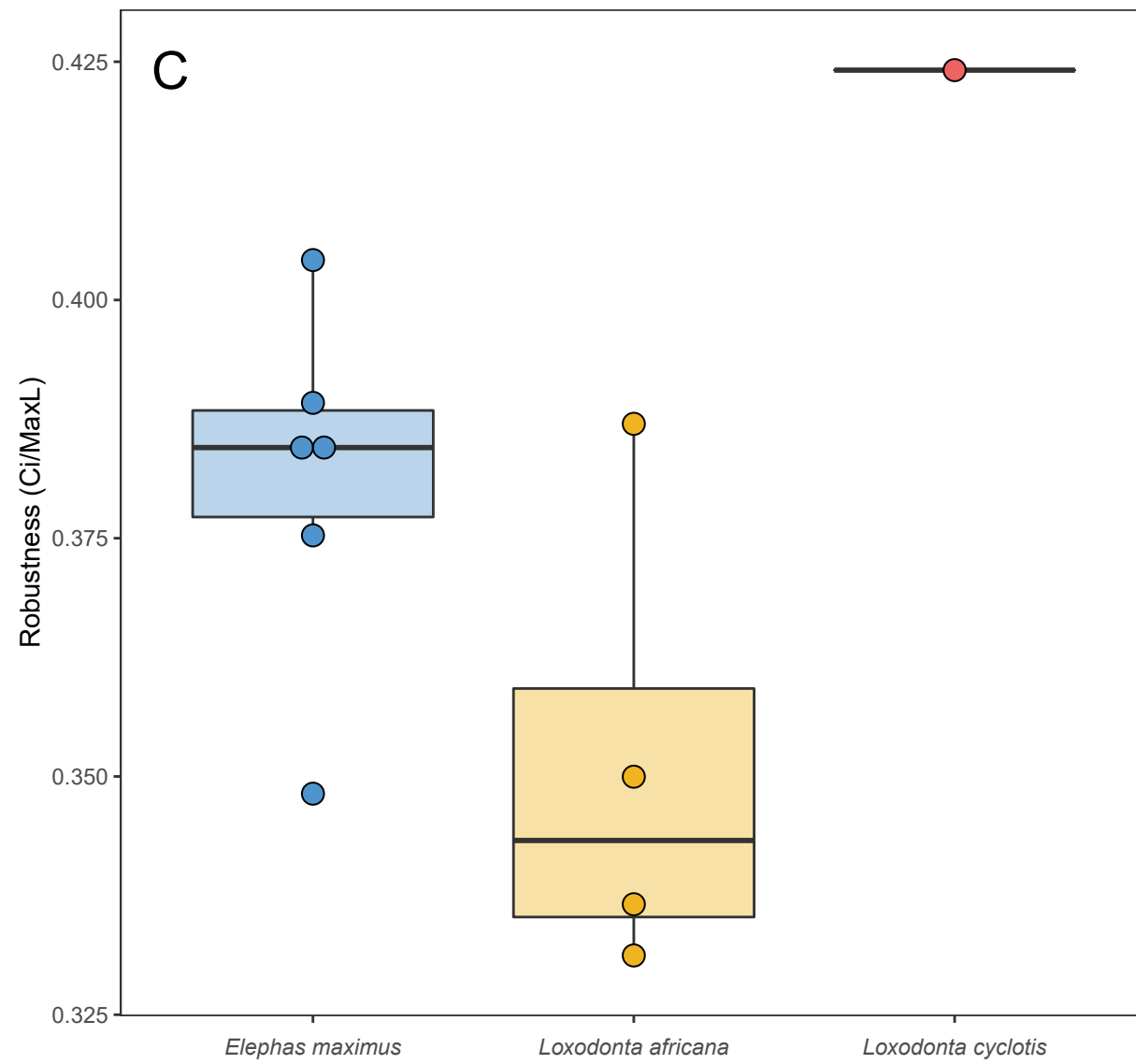

Fibula

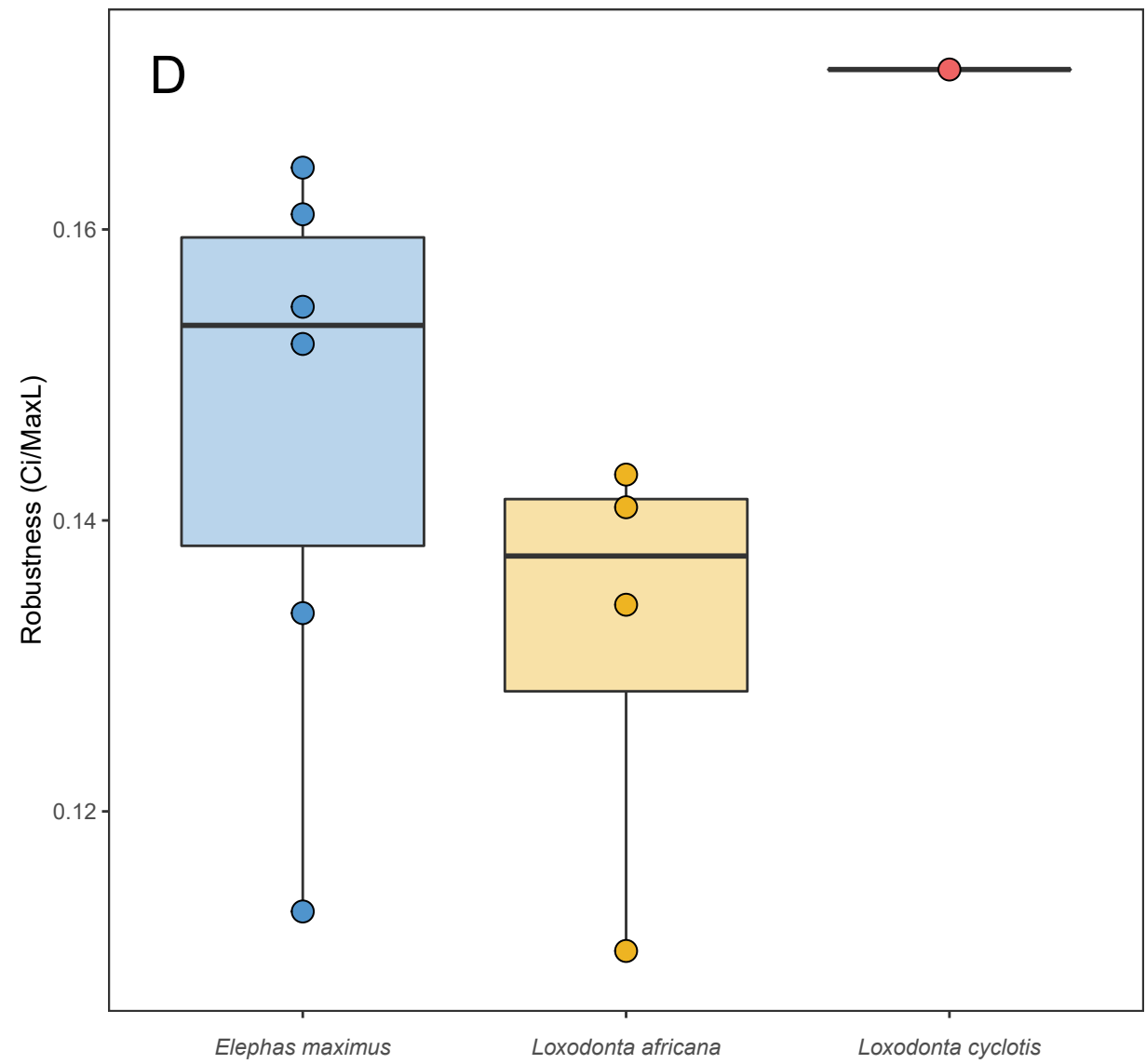

Supplement: Supplementary file 8 — Figure S8: [file JOA-242-806-s014.pdf]

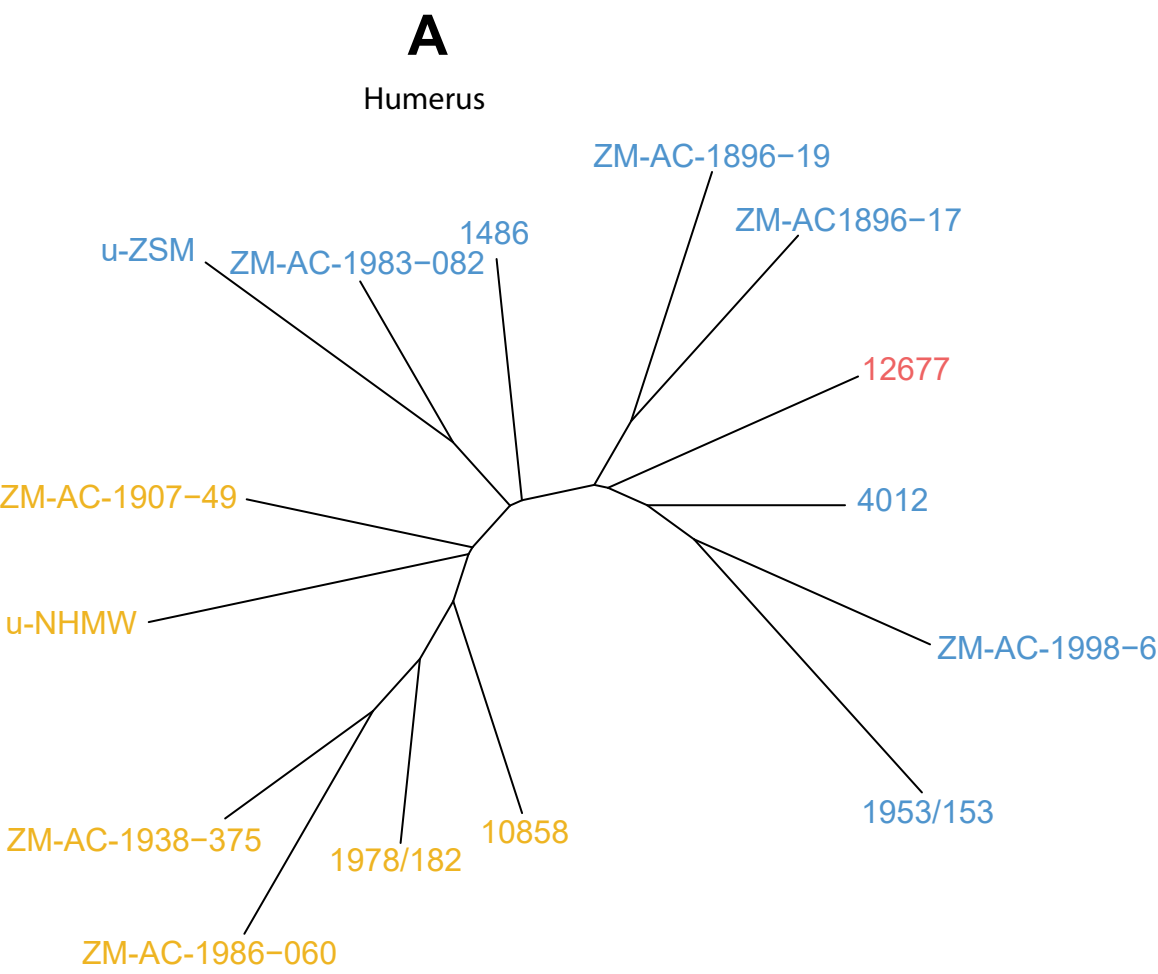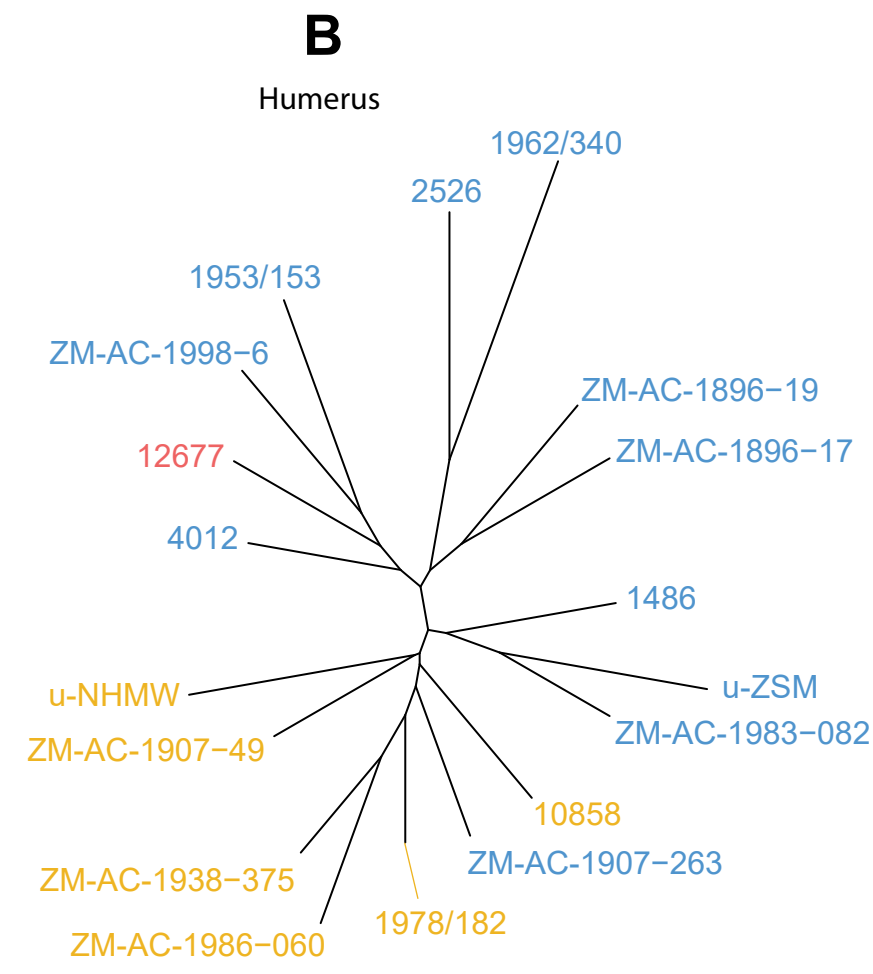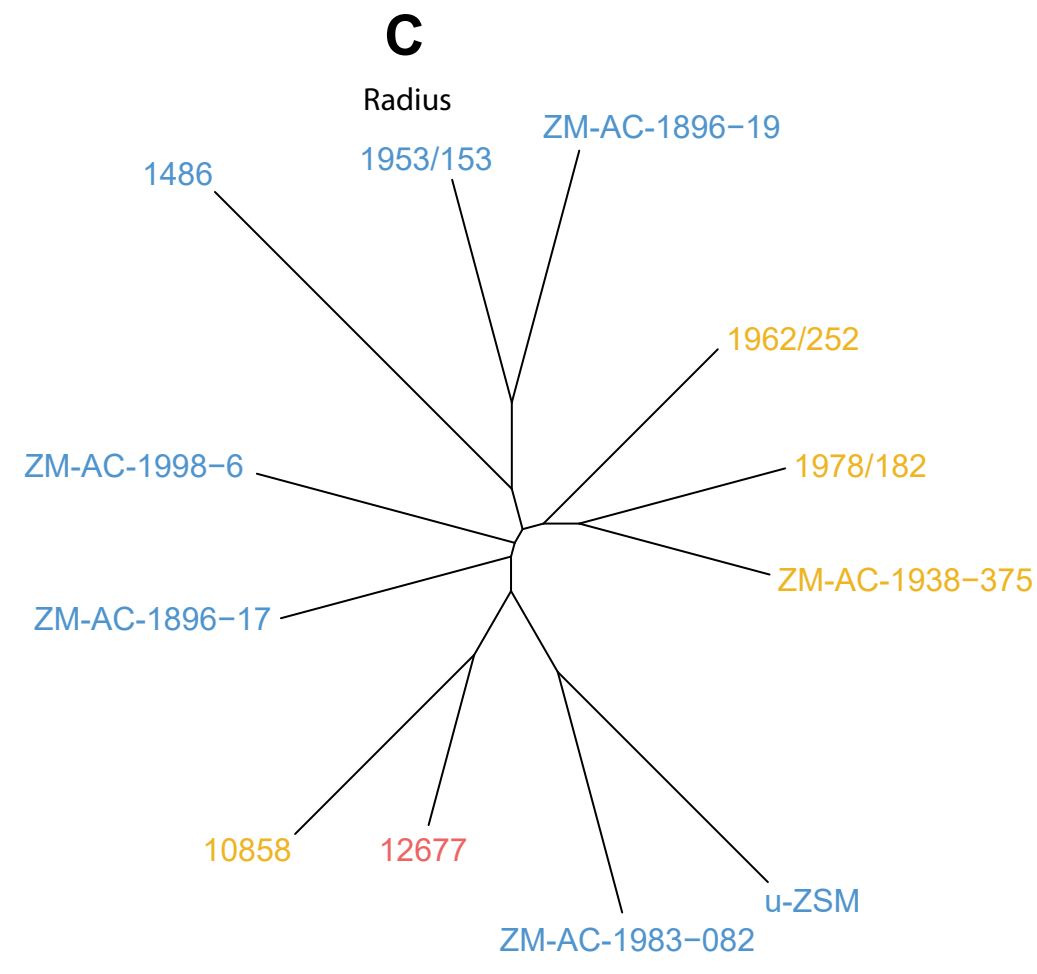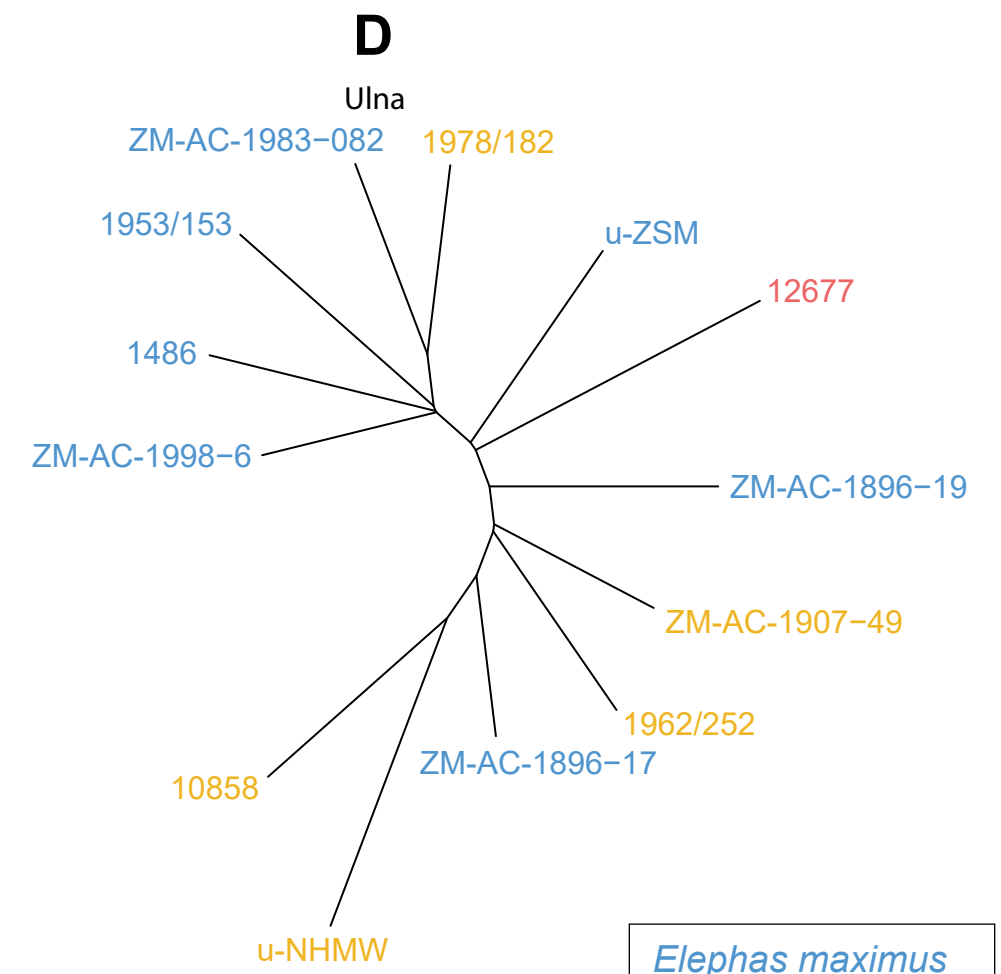

*Elephas maximus*  
*Loxodonta africana*  
*Loxodonta cyclotis*  
NA

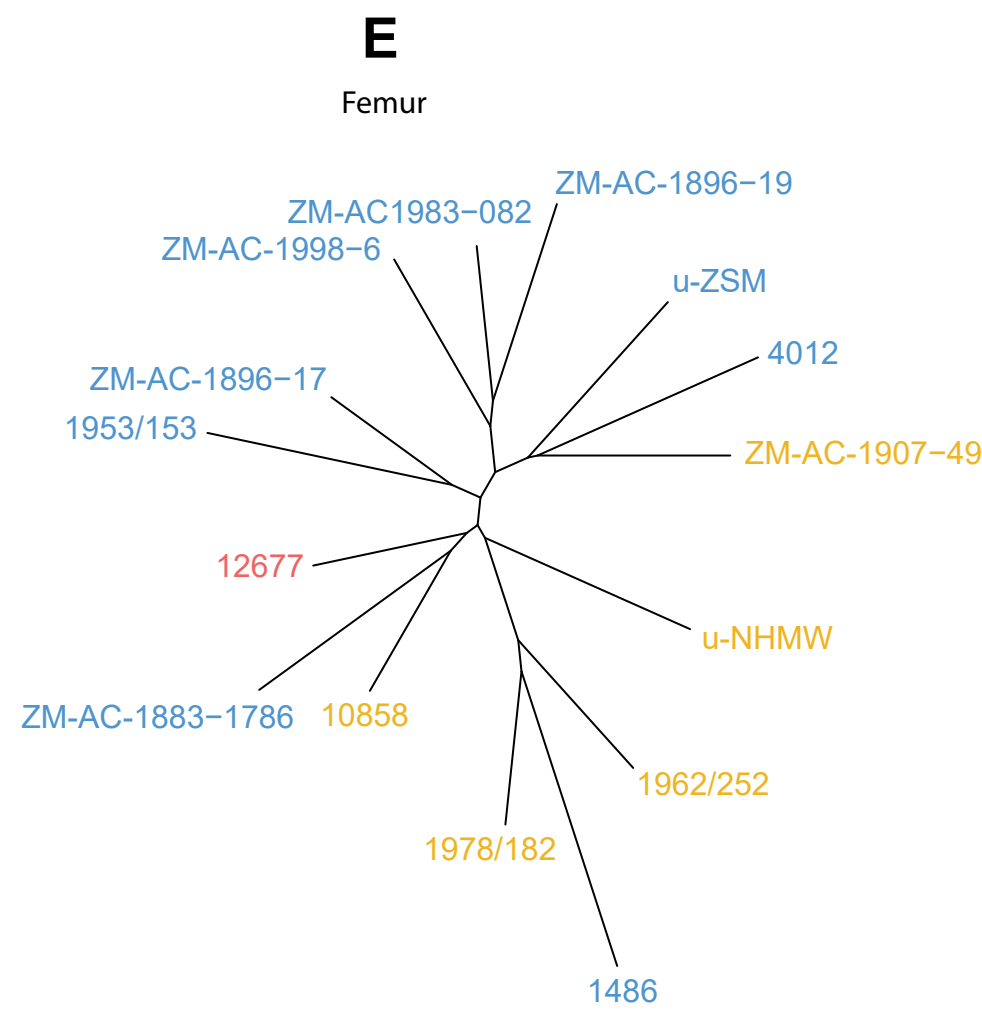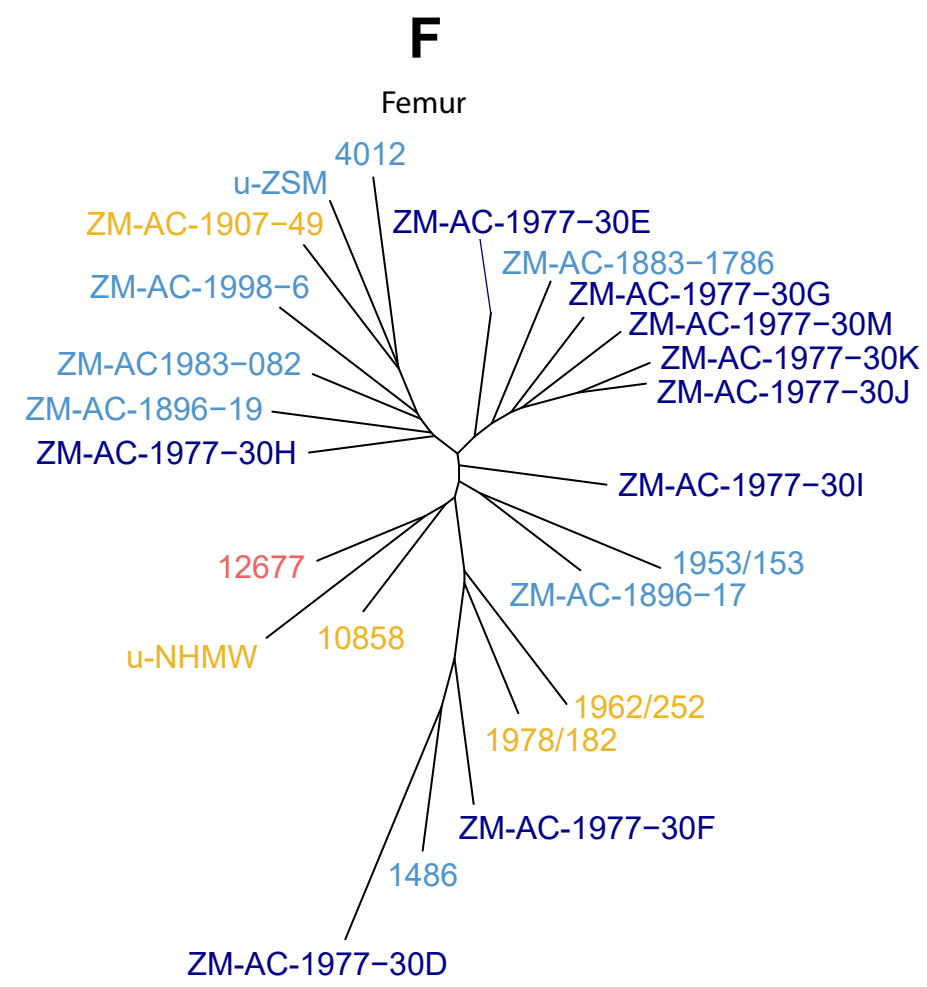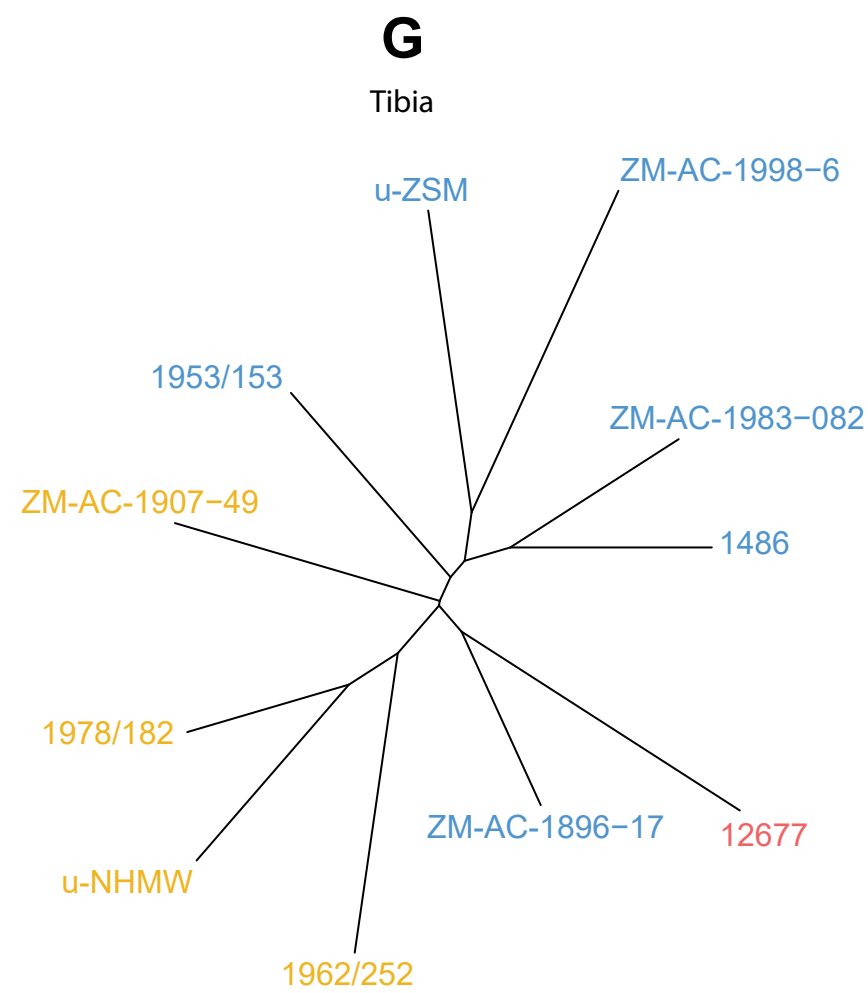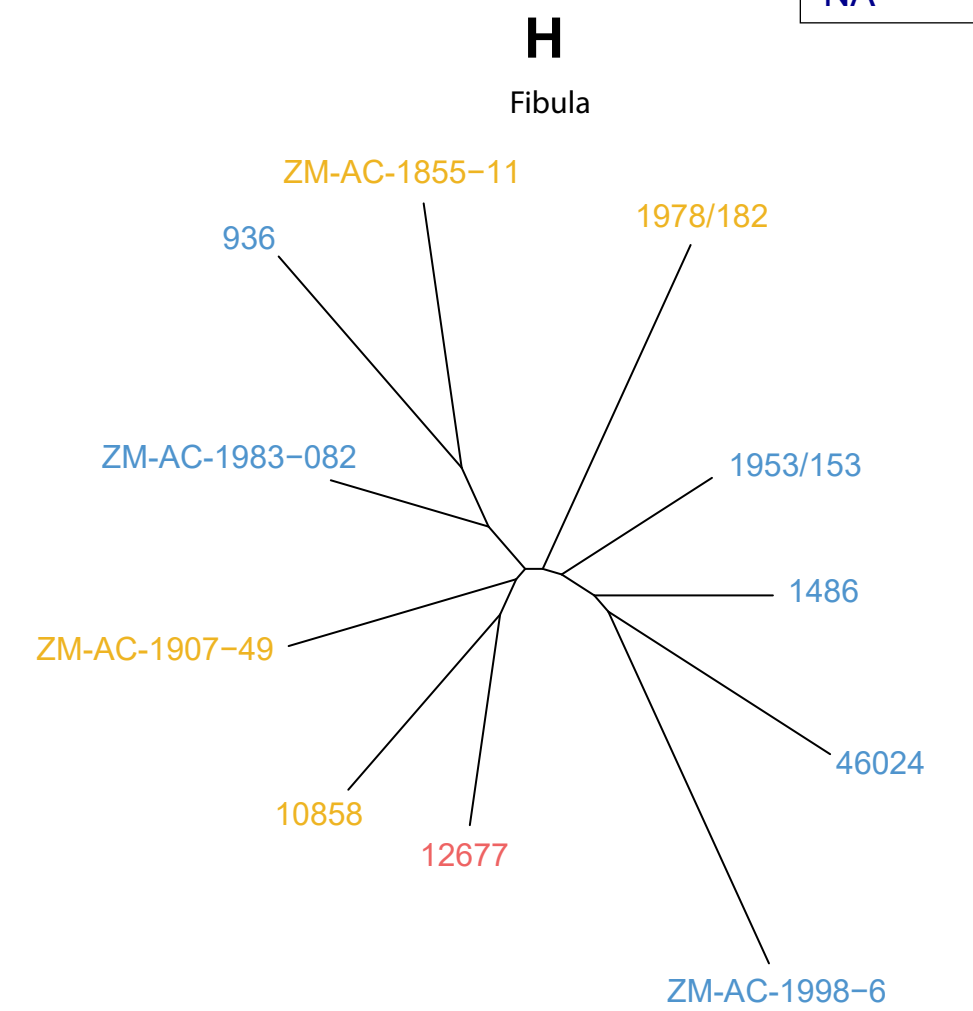

Supplement: Supplementary file 9 — Figure S9: [file JOA-242-806-s017.pdf]

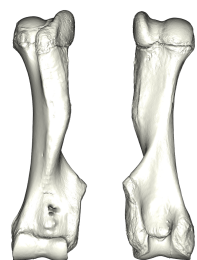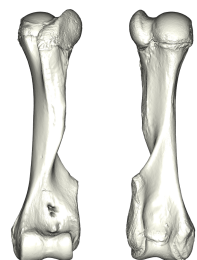

max  
↑  
PC2 (20.2%)  
↓  
min

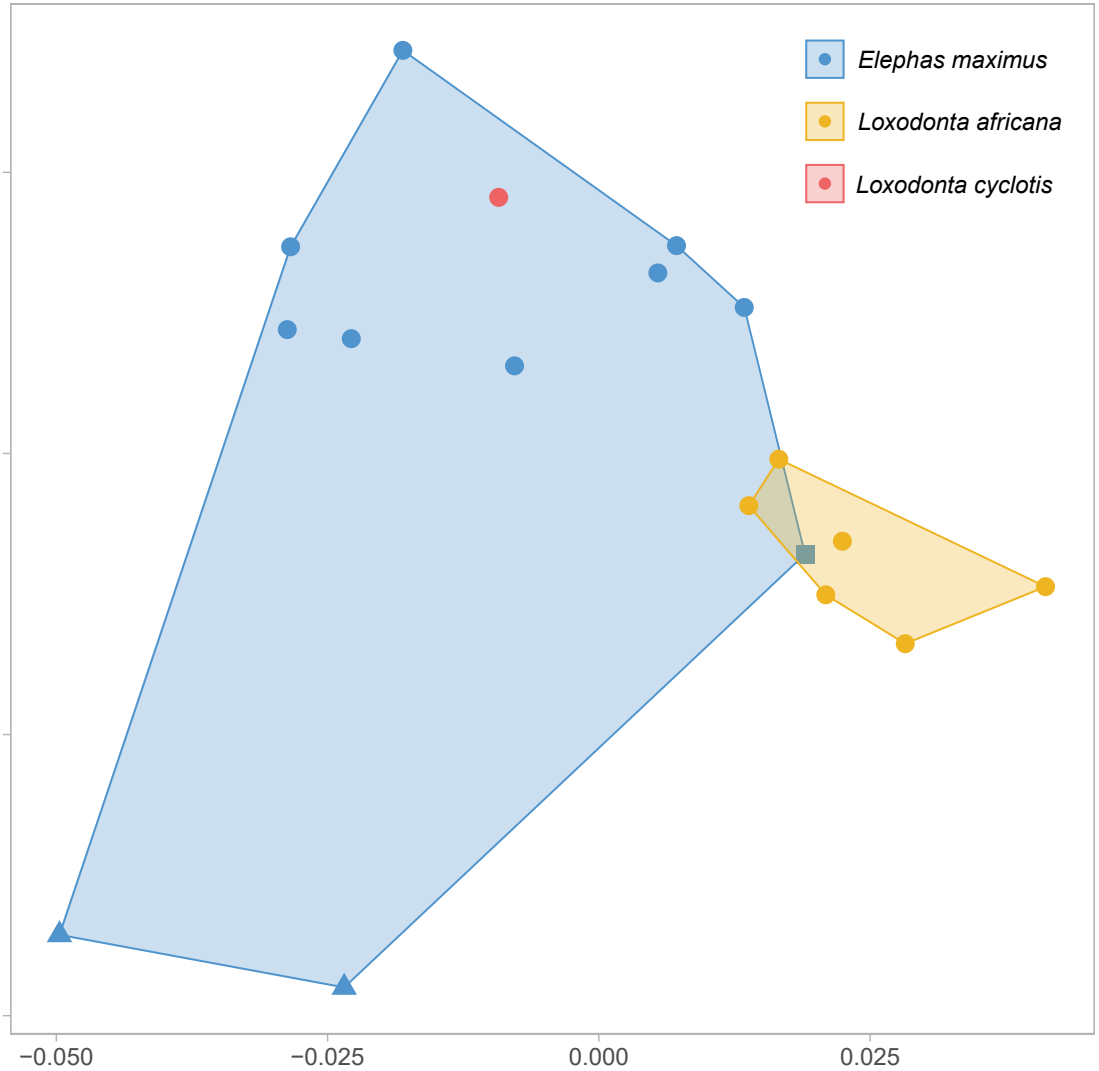

min ← PC1 (25.6%) → max

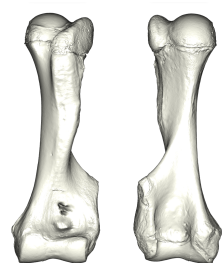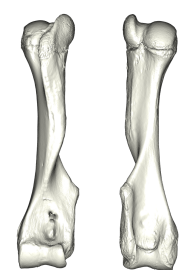

Humerus

Supplement: Supplementary file 10 — Figure S10: [file JOA-242-806-s016.pdf]

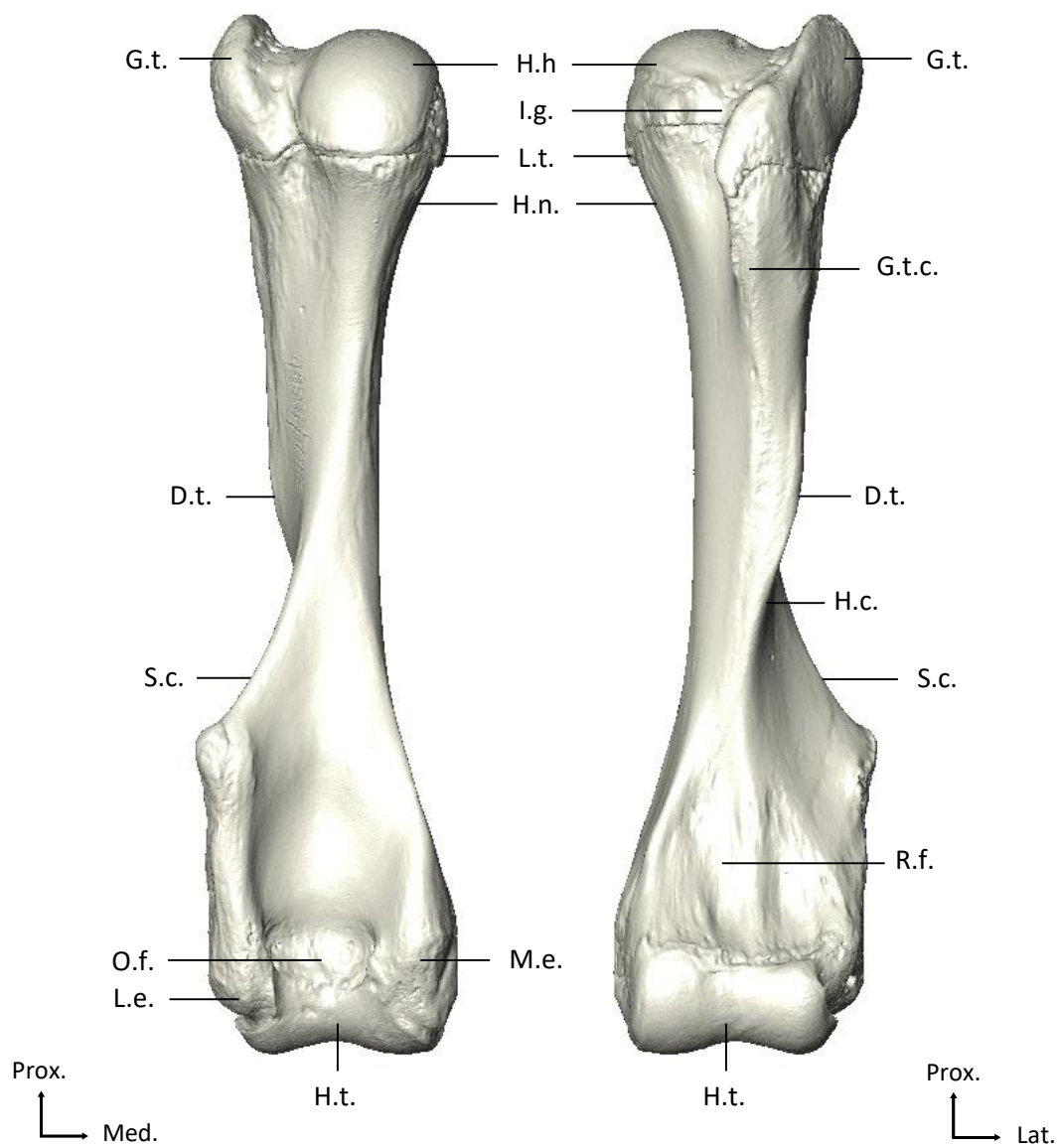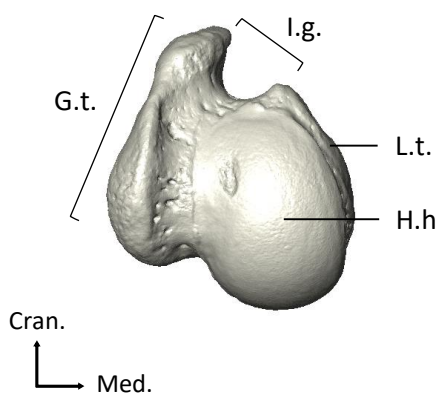

10 cm

Supplement: Supplementary file 11 — Figure S11: [file JOA-242-806-s011.pdf]

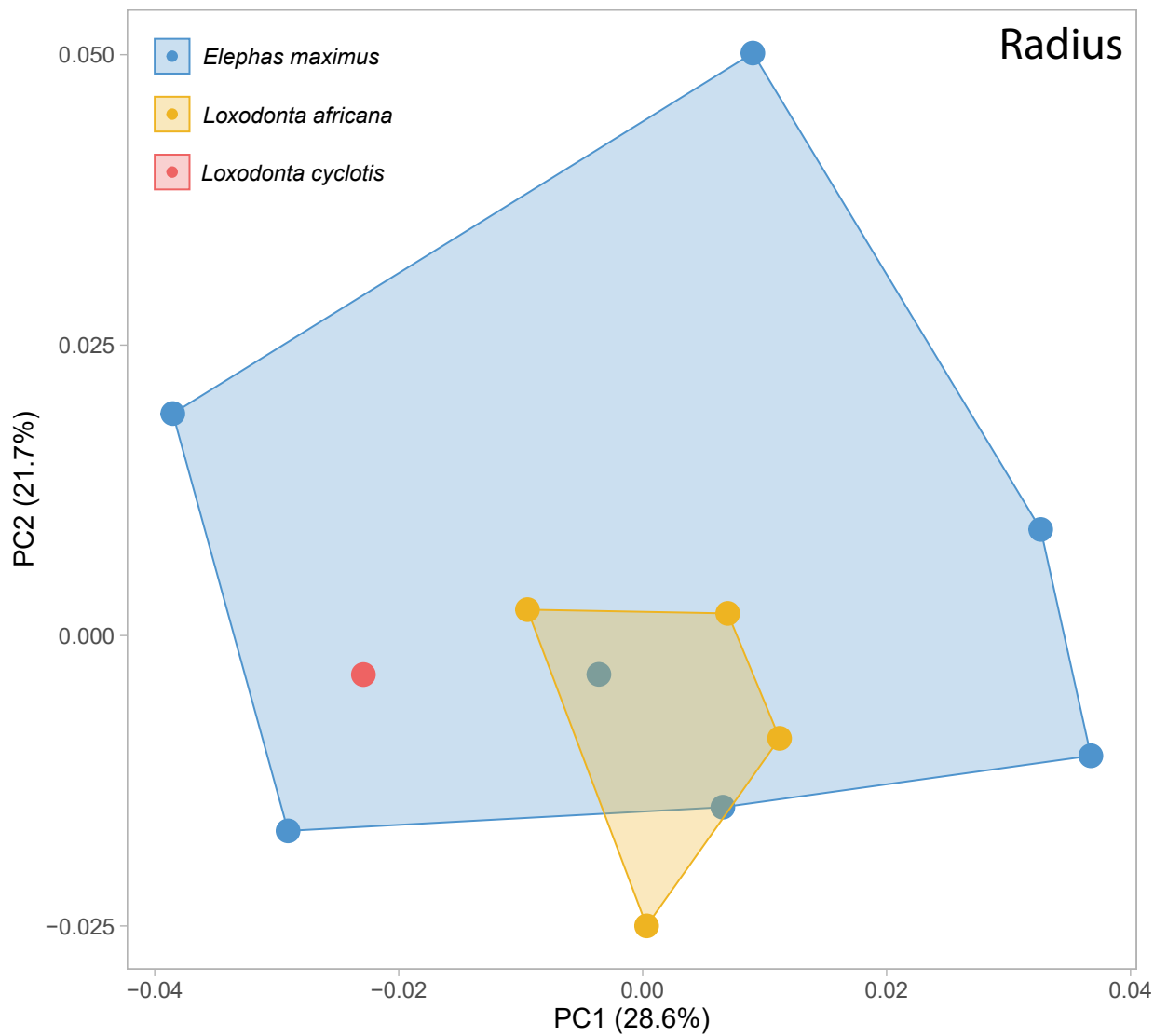

Supplement: Supplementary file 12 — Figure S12: [file JOA-242-806-s005.pdf]

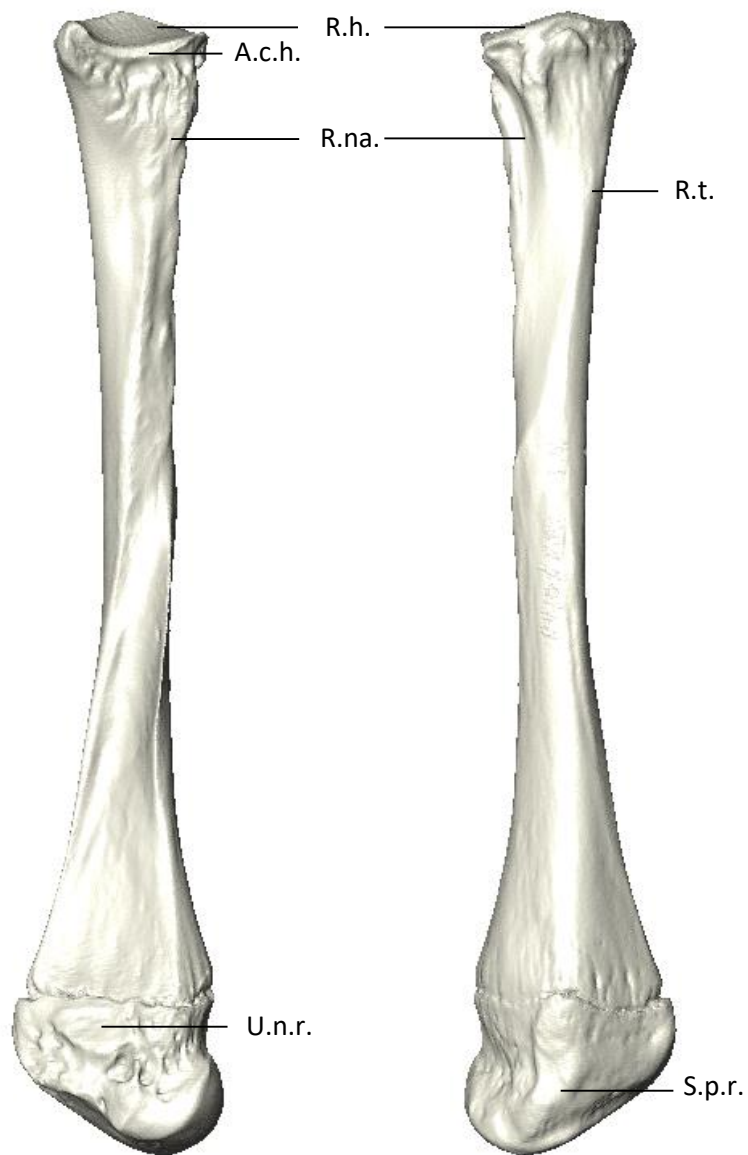

Prox.  
 Med.

Prox.  
 Lat.

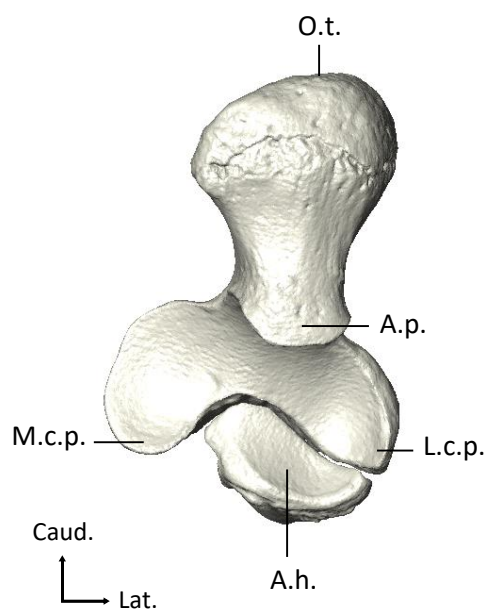

Caud.  
 Lat.

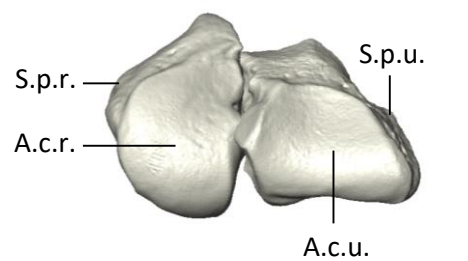

Cran.  
 Lat.

10 cm

Supplement: Supplementary file 13 — Figure S13: [file JOA-242-806-s007.pdf]

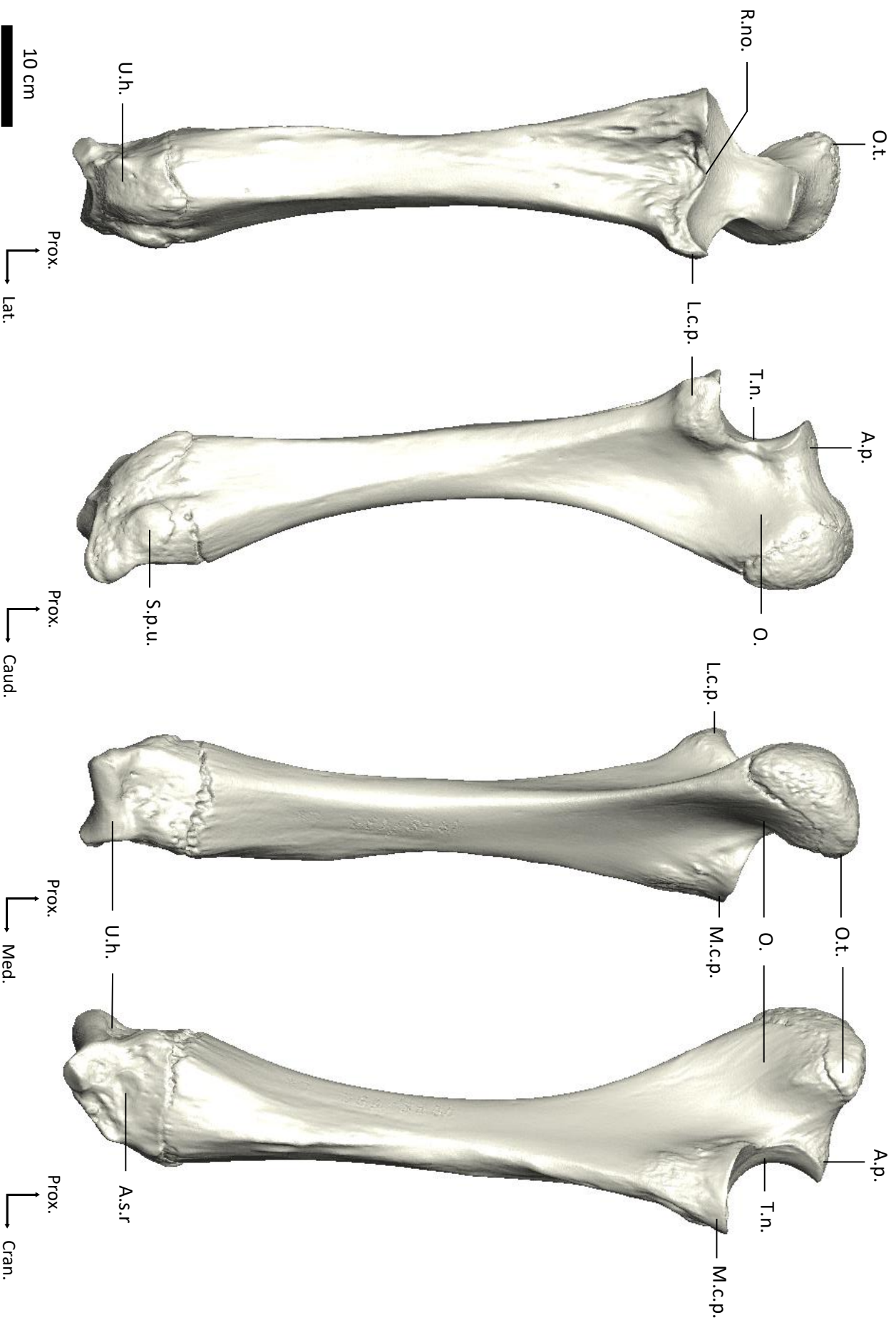

Supplement: Supplementary file 14 — Figure S14: [file JOA-242-806-s009.pdf]

# Femur

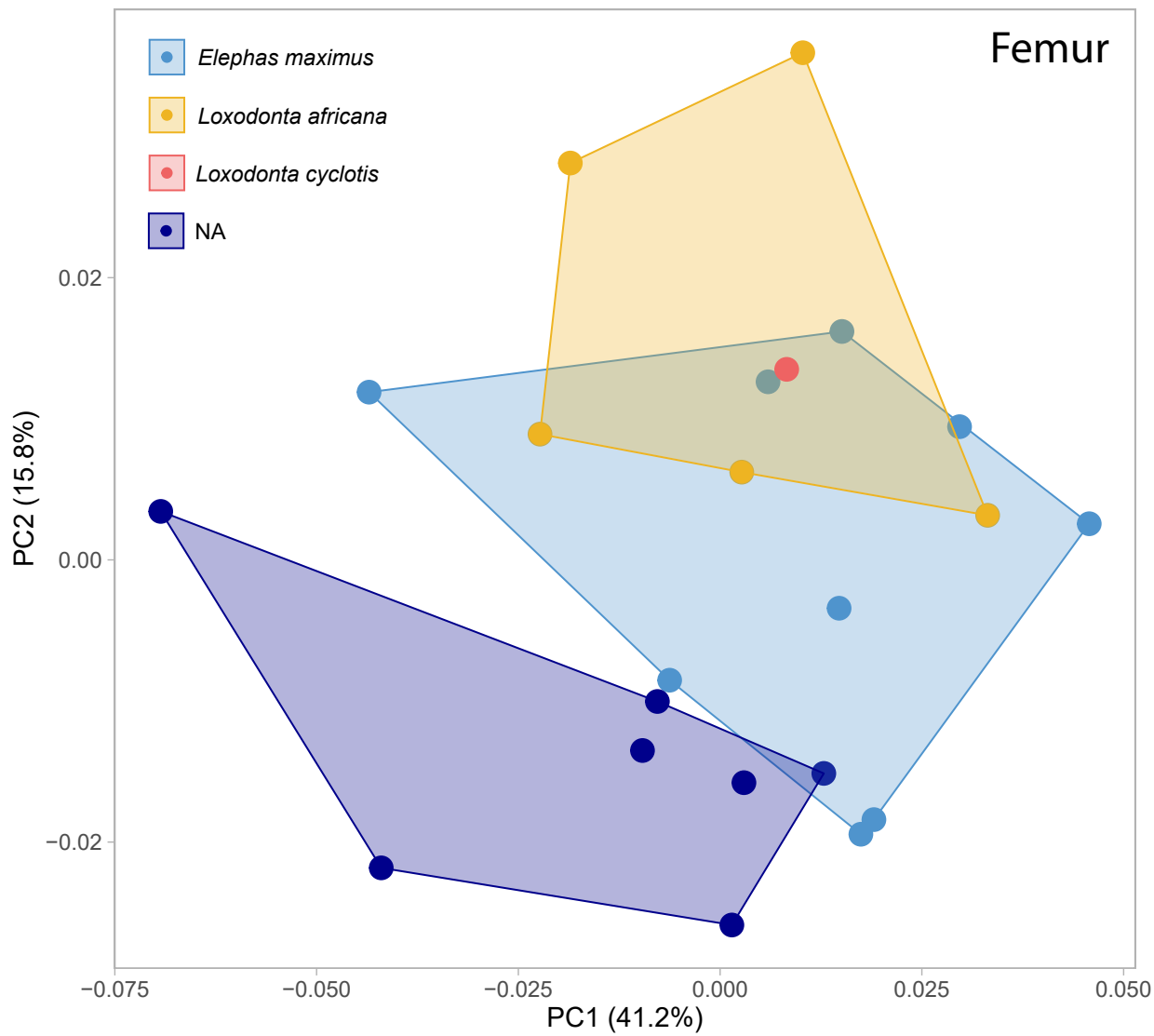

Supplement: Supplementary file 15 — Figure S15: [file JOA-242-806-s001.pdf]

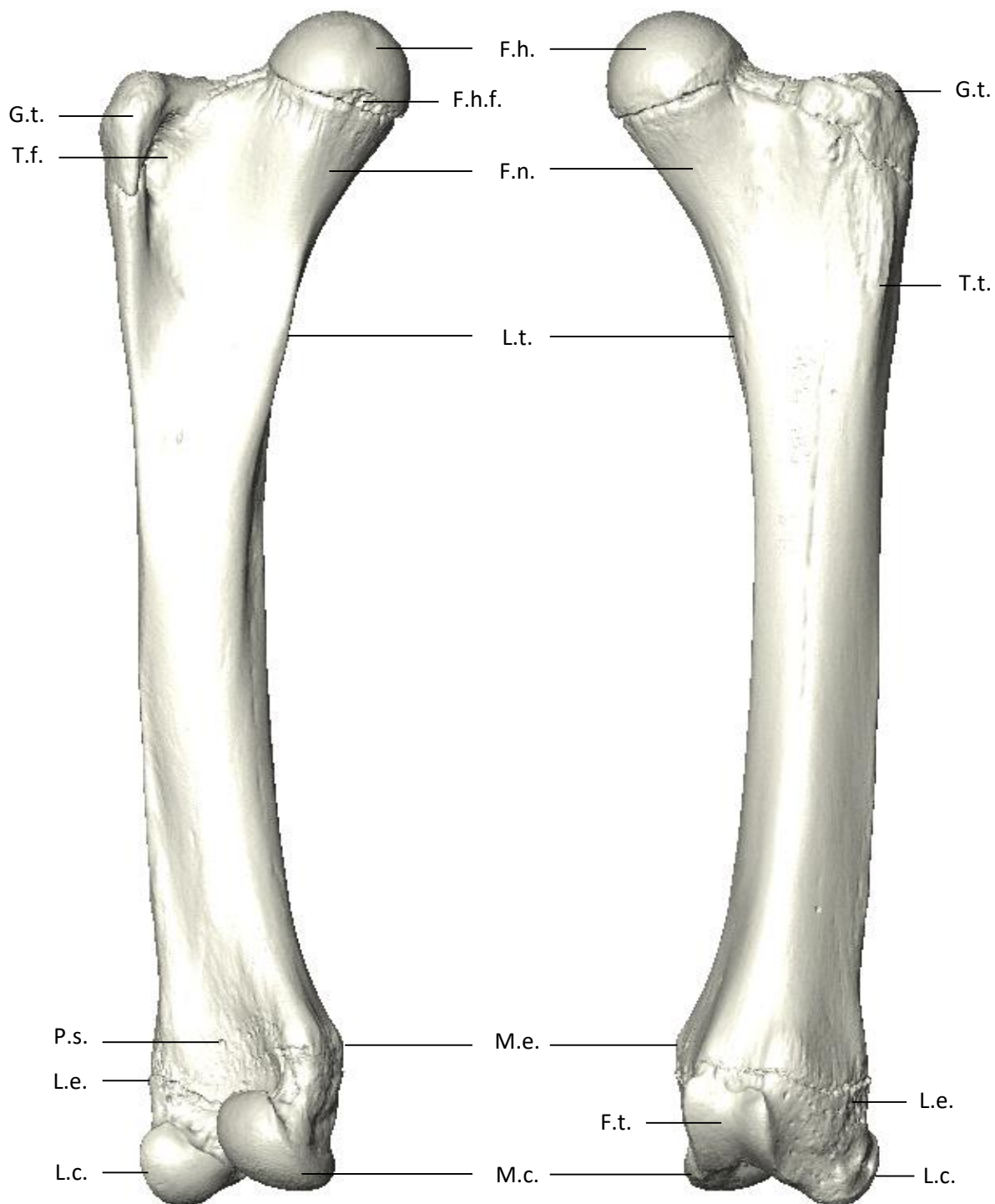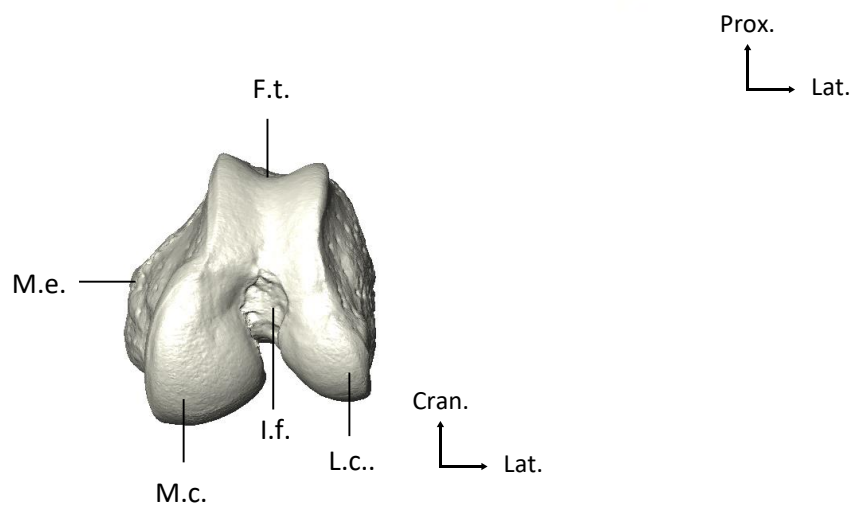

Supplement: Supplementary file 16 — Figure S16: [file JOA-242-806-s013.pdf]

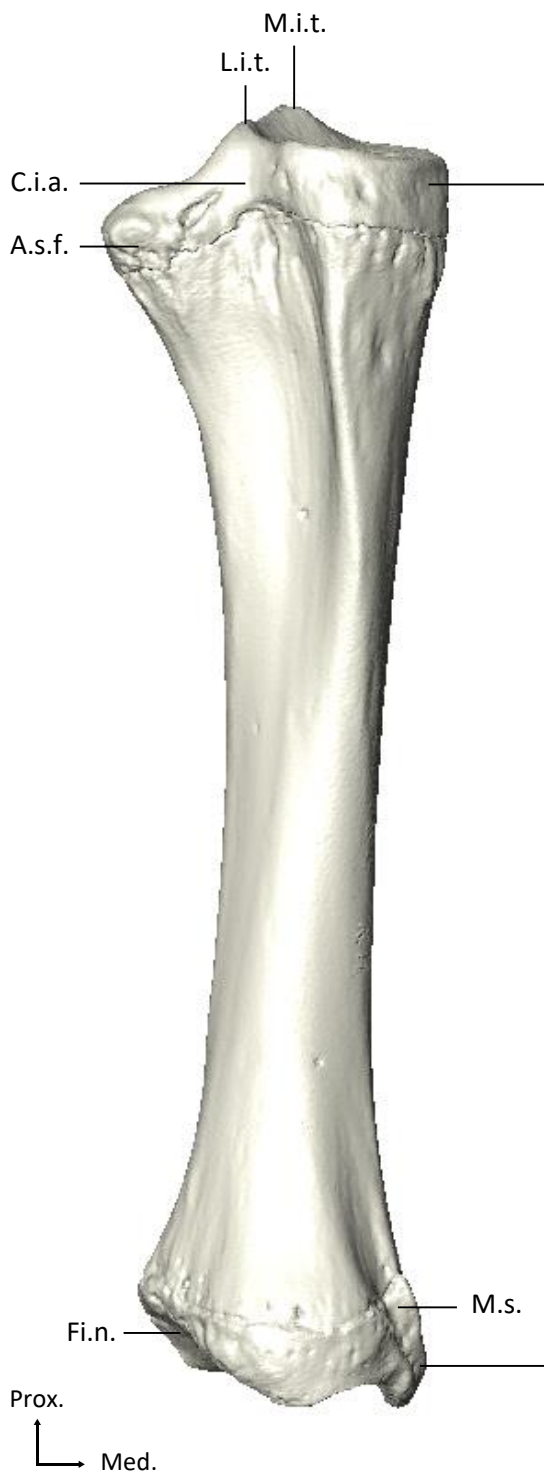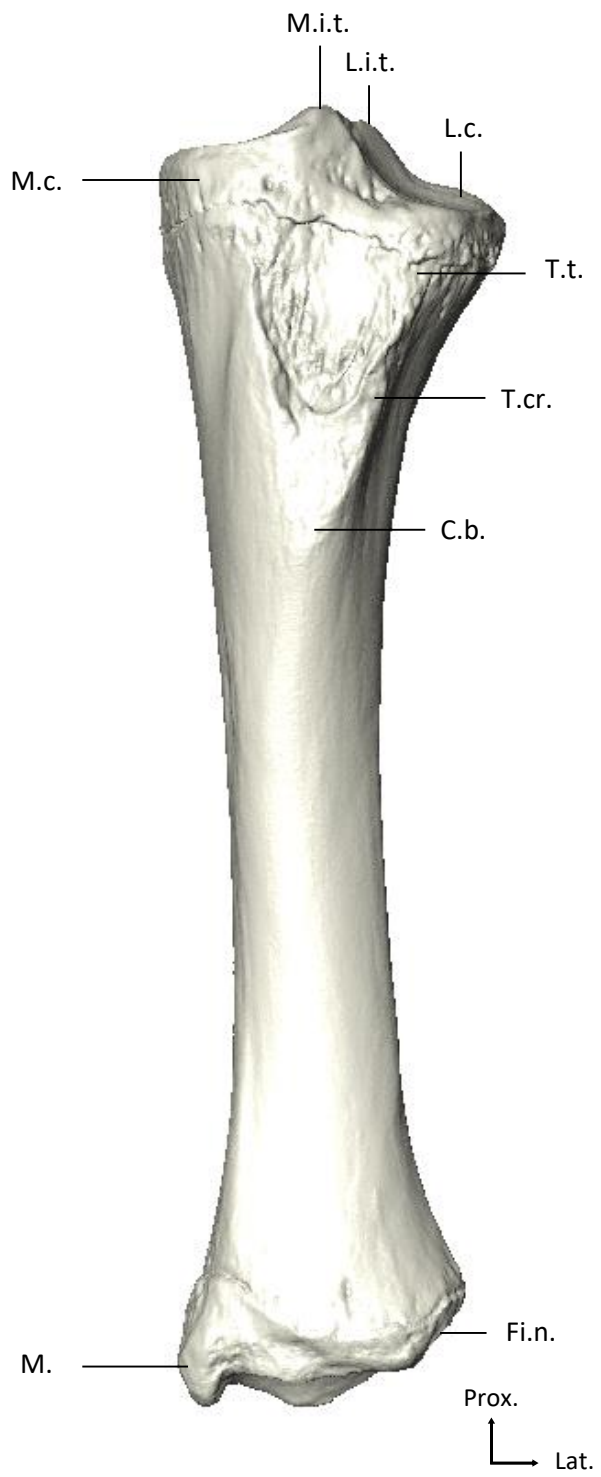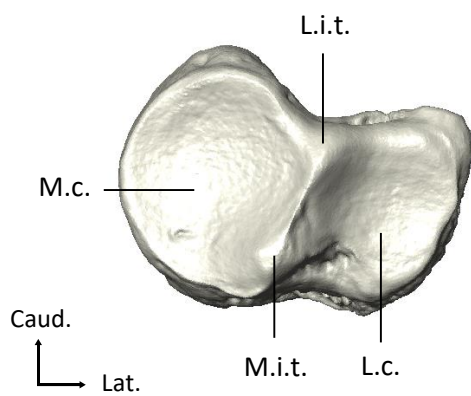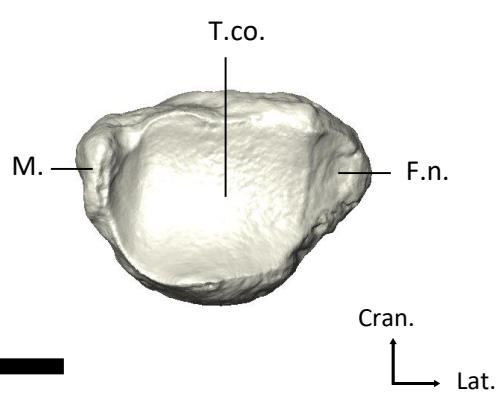

10 cm

Supplement: Supplementary file 17 — Figure S17: [file JOA-242-806-s019.pdf]

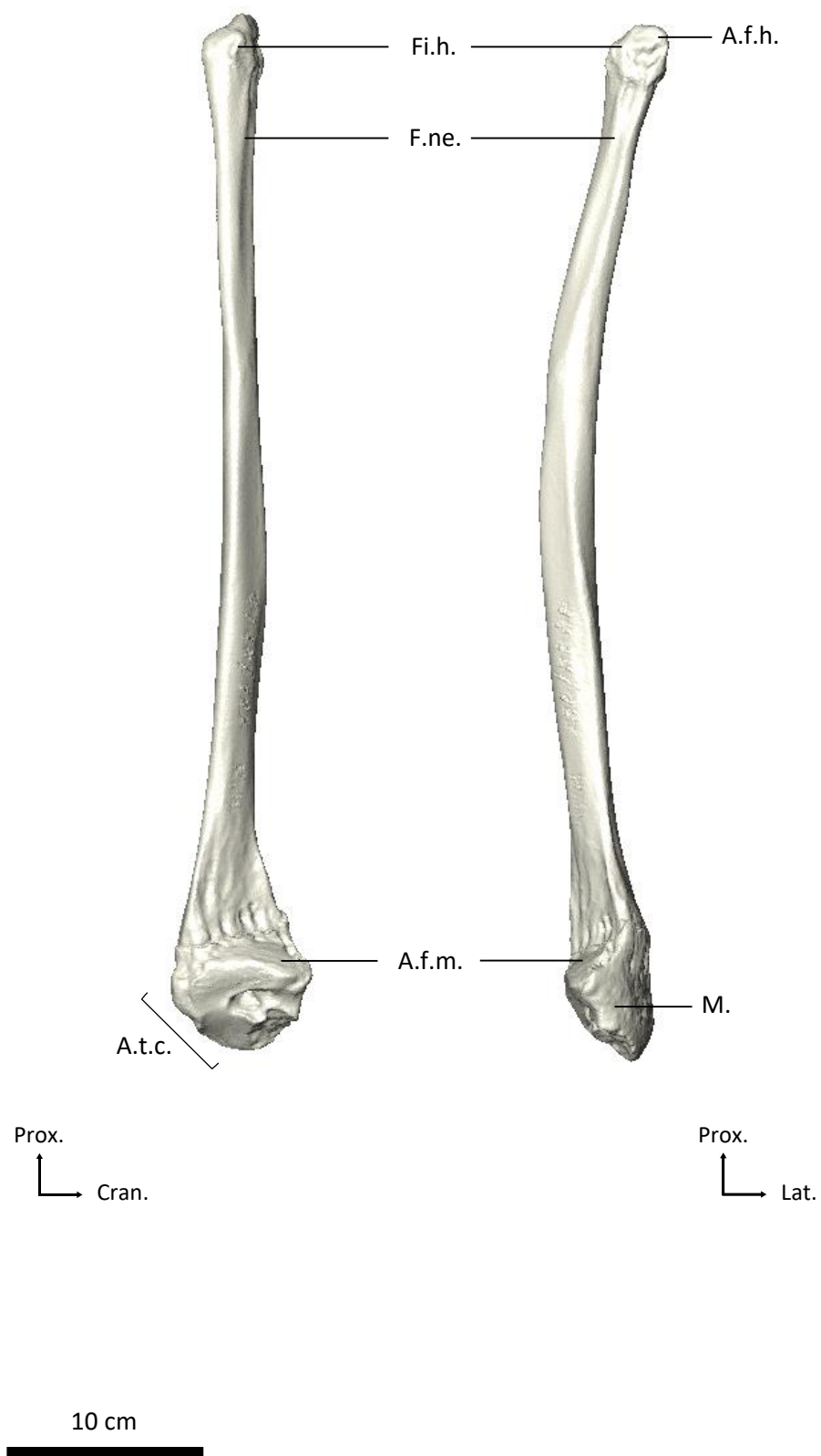

Supplement: Supplementary file 18 — Figure S18: [file JOA-242-806-s002.pdf]
