# Supplementary material for: Shape variation in the limb long bones of modern elephants reveals adaptations to body mass and habitat
Source: J Anat. 2023 Feb 23;242(5):806–30. doi: 10.1111/joa.13827 (PMC10093169; doi:10.1111/joa.13827)
Supplement: Supplementary file 19 — Data S1. [file JOA-242-806-s003.pdf]

Supplementary Results S1: Descriptions of the theoretical shapes at the first two PCs minimum and maximum.

Humerus

The theoretical shape at the PC1 minimum shows a massive and stout morphology, with wide epiphyses and a thick diaphysis; a large humeral head and a greater trochanter rounded and angled toward the lateral side, with its caudal delimitation placed more distally than the head; a wide deltoid tuberosity extending toward the medial side, forming a rounded and close-angled intertubercular groove and a deep depression on the cranial side of the bone, under the humeral head; a supracondylar crest with an angle of approximatively 45 degrees; a wide and deep olecranon fossa with large medial and lateral epicondyles; a medial epicondyle particularly developed and forming a protuberance on the caudal side; a central part of the trochlea markedly reduced relatively to the condyles. The theoretical shape at the PC1 maximum shows a thin and elongated morphology, with epiphyses extended in the cranio-caudal axis and overall less pronounced protuberances; a greater trochanter thinly shaped and extending farther than the humeral head proximally and forming a narrow angle on the dorsal side; the greater trochanter appears stretched in the dorso-ventral and cranio-caudal axes, forming an open angle with the most lateral part of the humeral head; the diaphysis is in the continuity of the greater trochanter on the lateral side, extending in a flat angle until/up to the deltoid tuberosity; the supracondylar extends laterally and distally with an angle of approximately 60 degrees, and slightly toward the caudal side; both epicondyles are flattened, with no visible protuberances; the width of the trochlea is reduced but stays close to that of the condyles.

The theoretical shape at the PC2 minimum shows a rounded greater trochanter, extended farther than the humeral head distally; the extremity of the supracondylar crest does not extend beyond the lateral limit of the greater trochanter; the medial epicondyle is small and poorly developed; the reduced width of the trochlea compared to the condyles is very pronounced; the entirety of the articular surface in contact with the ulna is large and well-developed. At the opposite, the theoretical shape at the PC2 maximum displays a flattened greater trochanter, extending farther than the humeral head in the caudal direction; the supracondylar crest forms a larger angle with the central part of the diaphysis and extends farther than the greater trochanter in the lateral direction; its lateral extremity is angled slightly toward the caudal and cranial directions; the medial epicondyle is large, forming a protuberance on the caudal side; the trochlea and the condyles appear small and cranio-caudally flattened.

Ulna

The theoretical shape at the PC1 minimum shows a wide and rounded olecranon tuberosity; a narrow anconeal process; the lateral part of the trochlear notch is thin and forms a sharp angle; the mediodistal part of the trochlear notch is angled in the dorsal direction; the distal epiphysis is thin and elongated in the dorsoventral axis; the medial articular surface for the carpal bones is narrow. The theoretical shape at the PC1 maximum shows a thinner olecranon tuberosity, almost flat on the medial and dorsal sides, with its medial extremity extending in the dorsomedial direction; the anconeal process is wide; the laterodistal part of the trochlear notch process is rounded; the medial part is angled slightly in the ventral direction; the angle between the proximal epiphysis and the diaphysis on the caudal side is more pronounced; the distal epiphysis appears roughly flat, with a larger medial articular surface for the carpal bones, and a wider, slightly concave lateral articular surface for the carpal bones.
The theoretical shape at the PC2 minimum displays a thick olecranon tuberosity reaching largely farther than the anconeal process in the dorsal direction, in the continuity of the diaphysis; the proximal extremity of the anconeal process is narrow, with both the medial and lateral borders extending in the caudal direction; both mediodistal and laterodistal parts of the trochlear notch are angled toward the distal part of the bone; the distal epiphysis displays a large lateral articular surface for the carpal bones, forming a concave border with the medial articular surface for the carpal bones. The theoretical shape at the PC2 maximum shows a shorter and slightly thinner olecranon tuberosity, angled in the caudal direction and forming a curve with the diaphysis; the proximal extremity of the olecranon process is wide; both medial and lateral parts of the trochlear notch are angled in the dorsal direction, forming rounded limits to the articular surface; the distal epiphysis shows a narrow lateral articular surface for the carpal bones, and correspondingly a wide medial articular surface for the carpal bones.

The mean shapes of each species differ in the proximal epiphysis: *E. maximus* displays a more massive olecranon, enlarged along the proximo-distal axis, than *L. africana*. *E. maximus* also displays a trochlear notch with extended articular surfaces, visible on both the medial and lateral sides, unlike in *L. africana*; finally, the olecranon tuberosity is wider in *L. africana*, and as a result the trochlear notch appears larger.

Tibia

The theoretical shape at the PC1 minimum shows a massive form, with wide epiphyses and a thick diaphysis; the lateral condyle is more rounded and smaller than the medial condyle, and is placed more distally; the tibial tuberosity extends in the caudal direction, forming a protuberance with the lateral condyle, visible in caudal view; on the caudal side, the tuberosity groove forms a large, roughly triangular surface delimited by the tibial tuberosity, the cranial border of the lateral articular surface and the distal extremity of the tibial crest; this surface is slightly depressed and extends on almost the entirety of the cranial face of the proximal epiphysis; the tibial crest is massive, reaching to approximately a third of the total bone length; on the distal epiphysis, the tibial cochlea forms a shallow and concave articular surface, shaped in a trapezoid with a wider border on the cranial side than on the caudal one; the distal articular surface for the fibula is narrow and obliquely angled, and the medial malleolus is slightly curved over the tibial cochlea, forming a concave surface. At the opposite, the theoretical shape at the PC1 maximum shows a more delicate morphology, with a thinner diaphysis in both mediolateral and craniocaudal axes. The lateral condyle is less rounded and slightly elongated in the craniocaudal direction, while the lateral condyle is more rounded on the cranial side; the lateral condyle is placed farther distally; the tuberosity groove is deeper and narrower; the tibial tuberosity is thinner and flatter, forming a small protuberance with the lateral condyle; the caudal intercondylar area is placed more proximally, following the caudal edge of the proximal epiphysis which is reduced in width in the dorsoventral axis; on the distal epiphysis, the medial malleolus is elongated distally and less curved over the tibial cochlea; the distal articular surface for the fibula is wider and less obliquely angled, occupying more space on the cranial side; the tibial cochlea appears more square with cranial and caudal edges more similar in width.

The theoretical shape at the PC2 minimum shows a thin and elongated morphology, the diaphysis and the epiphyses being reduced in both the craniocaudal and the lateromedial axes; the lateral condyle is angled obliquely; the medial intercondylar tubercle is sharp and elongated proximally; the medial condyle displays a deep concavity; the tibial crest is short and thin, “limited” to the cranial side while the medial condyle forms a thick protuberance on the laterocaudal side; the tuberosity groove is shallow and the proximal edge of the triangle that it forms is reduced in width; on the distal epiphysis, the tibial cochlea displays a deep concavity, separated by a sharp ridge from the distal articular surface for the fibula on the lateral side, and a rounded medial malleolus. The theoretical shape at the PC2 maximum shows a more massive morphology, with wide epiphyses and a large diaphysis. The medial intercondylar tubercle is placed more medially, breaking the rounded shape of the medial condyle; the lateral condyle forms a right angle with the central intercondylar area; the tibial crest is large and joins the lateral condyle in the caudal direction, where it forms a thin protuberance; the tuberosity groove

forms a deep and wide concavity, angled slightly to the lateral side, following the tibial crest; on the distal epiphysis, the tibial cochlea appears almost square, with a reduced articular surface for the fibula on the lateral side, and a large and straight medial malleolus on the medial side.
