# Supplementary material for: Shape variation in the limb long bones of modern elephants reveals adaptations to body mass and habitat
Source: J Anat. 2023 Feb 23;242(5):806–30. doi: 10.1111/joa.13827 (PMC10093169; doi:10.1111/joa.13827)
Supplement: Supplementary file 20 — Tables S1–S6. [file JOA-242-806-s006.docx]

# SUPPLEMENTARY MATERIAL

**Table S1:** Designation of anatomical landmarks on the humerus.

| LM | Designation |
| --- | --- |
| 1 | Most disto-medial point of the greater trochanter |
| 2 | Most disto-caudal point of the greater trochanter |
| 3 | Most disto-lateral point of the border of the head |
| 4 | Most disto-medial point of the border of the head |
| 5 | Most medial point of the lesser tubercle |
| 6 | Most caudo-medial point of the intertubercular groove |
| 7 | Most caudo-medial point of the border of the head |
| 8 | Most caudo-lateral point of the border of the head |
| 9 | Most cranial point of the deltoid tuberosity |
| 10 | Most lateral point of the supracondylar crest |
| 11 | Most caudo-lateral point of the trochlea |
| 12 | Most caudo-medial point of the trochlea |
| 13 | Most cranio-medial point of the trochlea |
| 14 | Most cranio-lateral point of the trochlea |

**Table S2:** Designation of anatomical landmarks on the radius.

| LM | Designation |
| --- | --- |
| 1 | Most lateral point of the articular surface of the head |
| 2 | Most cranio-medial point of the articular surface of the head |
| 3 | Most lateral point of the head |
| 4 | Most disto-medial point of the head |
| 5 | Most caudal point of the epiphyseal line |
| 6 | Most medial point of the epiphyseal line |
| 7 | Most cranial point of the epiphyseal line |
| 8 | Most lateral point of the epiphyseal line |
| 9 | Most caudal point of the border of the articular surface for the carpal bones |
| 10 | Most medial point of the border of the articular surface for the carpal bones |
| 11 | Most cranio-medial point of the border of the articular surface for the carpal bones |
| 12 | Most cranial point of the border of the articular surface for the carpal bones |

**Table S3:** Designation of anatomical landmarks on the ulna.

| LM | Designation |
| --- | --- |
| 1 | Most cranial point of the medial condyle |
| 2 | Most cranial point of the lateral condyle |
| 3 | Maximum concavity point of the distal border of the trochlear notch articular surface |
| 4 | Most cranial point of the anconeal process |
| 5 | Most cranio-lateral point of the olecranon tuberosity |
| 6 | Most proximal point of the olecranon tuberosity |
| 7 | Most disto-cranial point of the lateral condyle |
| 8 | Most distal point of the lateral crest |
| 9 | Most disto-caudal point of the olecranon tuberosity |
| 10 | Most cranial point of the epiphyseal line |
| 11 | Most caudal point of the epiphyseal line |
| 12 | Most medial point of the epiphyseal line |
| 13 | Most proximo-caudal point of the articular surface for the carpal bones |
| 14 | Most proximo-medial point of the articular surface for the carpal bones |
| 15 | Most cranio-lateral point of the articular surface for the carpal bones |

**Table S4:** Designation of anatomical landmarks on the femur.

| LM | Designation |
| --- | --- |
| 1 | Most lateral point of the border of the head |
| 2 | Most medial point of the border of the head |
| 3 | Most proximo-medial part of the trochanteric fossa |
| 4 | Most disto-caudal point of the greater trochanter |
| 5 | Most proximo-medial point of the third trochanter |
| 6 | Most disto-medial point of the third trochanter |
| 7 | Most distal point of the lesser trochanter |
| 8 | Most caudal point of the medial epicondyle |
| 9 | Most lateral point of the lateral epicondyle |
| 10 | Most proximal point of the lateral lip of the trochlea |
| 11 | Most proximal point of the medial lip of the trochlea |
| 12 | Most distal point of the medial lip of the trochlea |
| 13 | Most distal point of the lateral lip of the trochlea |
| 14 | Distal maximum of curvature of the trochlear groove |
| 15 | Most proximal point of the medial condyle |
| 16 | Most proximal point of the lateral condyle |

**Table S5:** Designation of anatomical landmarks on the tibia.

| LM | Designation |
| --- | --- |
| 1 | Most proximal point of the medial intercondylar tubercle |
| 2 | Most proximal point of the lateral intercondylar tubercle |
| 3 | Maximum of curvature of the medial border of the medial epicondyle |
| 4 | Maximum of curvature of the caudo-lateral border of the lateral condyle |
| 5 | Most cranial point of the cranial part of the medial condyle |
| 6 | Maximum of concavity of the cranial side of the epiphyseal line |
| 7 | Most cranial point of the tibial tuberosity |
| 8 | Most cranial point of the cranial border of the tibia |
| 9 | Most lateral point of the articular surface for the fibula |
| 10 | Most caudal point of the caudal side of the medial epicondyle |
| 11 | Most caudo-medial point of the malleolar sulcus |
| 12 | Most lateral point of the diaphysis |
| 13 | Most cranio-medial point of the border of the cochlea |
| 14 | Most caudo-medial point of the border of the cochlea |
| 15 | Most caudal point of the limit between the cochlea and the fibular notch |
| 16 | Most cranial point of the limit between the cochlea and the fibular notch |
| 17 | Most lateral point of the fibular notch |
| 18 | Most distal point of the malleolus |

**Table S6:** Designation of anatomical landmarks on the fibula.

| LM | Designation |
| --- | --- |
| 1 | Most cranio-medial point of the head |
| 2 | Most disto-cranial point of the articular facet of the malleolus |
| 3 | Most proximal point of the head |
| 4 | Most medial point of the head |
| 5 | Proximo-lateral limit of the articular facet for the talus and calcaneus |
| 6 | Most medial point of the articular facet for the talus and calcaneus |
| 7 | Most disto-medial point of the articular facet for the talus and calcaneus |
| 8 | Most lateral point of the articular facet for the talus and calcaneus |
| 9 | Proximo-lateral limit of the articular facet for the talus and calcaneus |
| 10 | Most proximal point of the epiphyseal line |
